# Stereodivergent Synthesis of Alkaloid (±)-223A and (±)-6-*epi*-223A via Rh-Catalyzed Hydroformylation Double Cyclization

**DOI:** 10.1021/acs.joc.3c02366

**Published:** 2024-03-08

**Authors:** Wen-Wei Huang, Jui-Teng Cheng, Wei-Ting Hsiao, Wen-Hua Chiou

**Affiliations:** Department of Chemistry, National Chung-Hsing University, Taichung 402202, Taiwan, R.O.C.

## Abstract

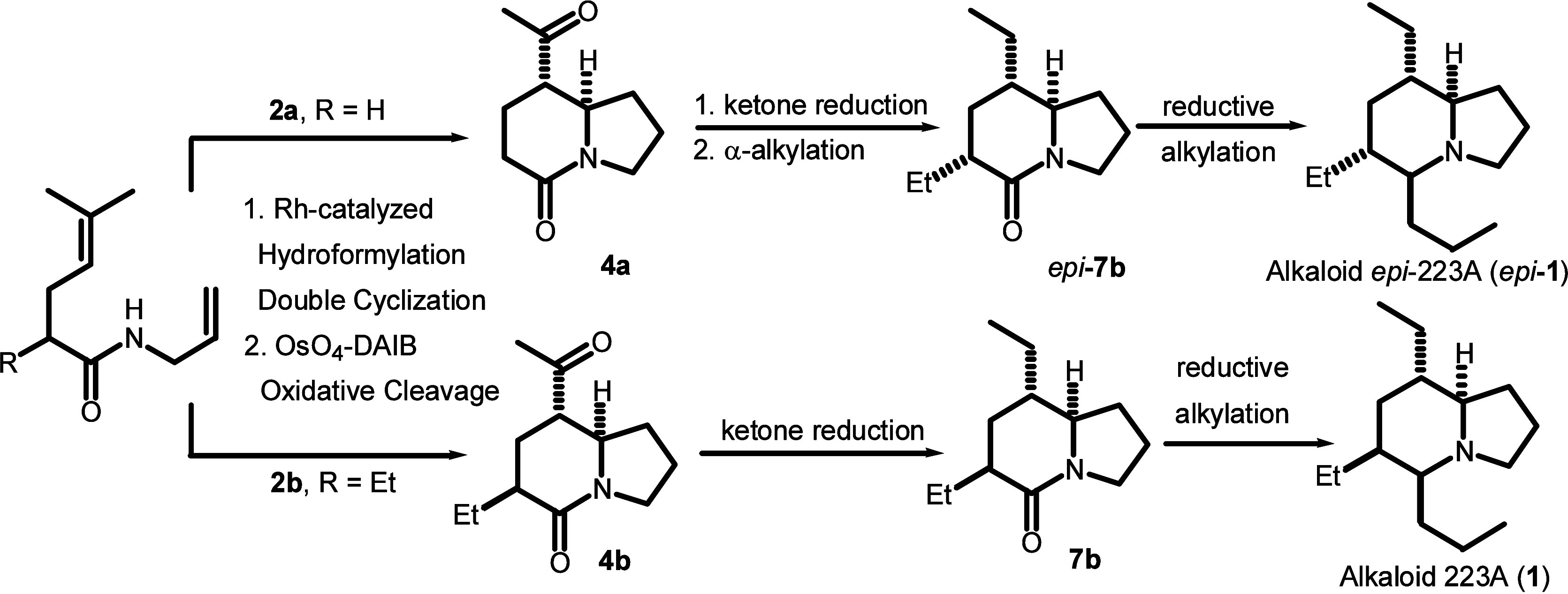

A stereodivergent approach toward total syntheses of *Dendrobatid* alkaloids 223A and 6-*epi*-223A is described. The approach features a concise construction of an indolizidine skeleton by Rh-catalyzed domino hydroformylation double cyclization and sequential stereocontrolled transformations such as reductive alkylation or *anti*-selective α-alkylation of the 5-oxoindolizidine. These stereoselective reactions afford the desired stereochemistry in the targets.

In 1997, Daly et al. reported the isolation of the first trisubstituted indolizidine alkaloid 223A (**1**), from a dendrobatid frog *Dendrobates pumilio Schmidt*, in Panama.^1^ The strucure of alkaloid 223A was originally proposed as the structure of 6-*epi*-223A (6-*epi*-**1**) shown in Figure 1 and was revised as structure **1** with a *cis* configuration between H-6 and bridged proton H-8a, through total synthesis by Toyooka and co-workers in 2002. These dart frog poisons display blocking effects on nicotinic acetylcholine receptors,^2^ and their use has pharmaceutical potential for neurological disorders.^3^ The correct introduction of the stereocenters represents the main challenge in the synthesis of the indolizidine system. Thus, various intellectual and elegant strategies were developed: Michael-type conjugate addition on a 2-piperideine system,^4,5^ via an aza-Achmatowicz oxidation of a furfuryl system,^6^ amine-mediated Michael addition to ynone followed by cyclization,^7,8^ trialkylsilyltin-mediated cyclization of allene,^9^ intramolecular Mannich reaction of a β-aminobutanone derivative,^10^ intramolecular cyclization of a homoprolinate derivative,^11,12^ intramolecular Schmidt reaction followed by ring opening metathesis,^13^ and elaboration of a versatile tricyclic lactone precursor.^14^ These approaches can be divided into two categories: formation of the piperidine ring prior to formation of the pyrrolidine ring and vice versa. Here we describe a one-step domino approach to these two alkaloids.

**Figure 1:**
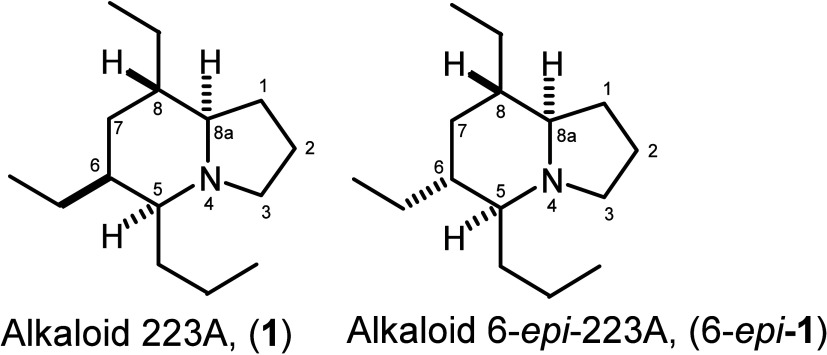
Revised structures of alkaloids 223A (**1**) and 6-*epi*-223A (6-*epi*-**1**).

Recently, we reported a domino Rh-catalyzed hydroformylation double cyclization process, which provides a versatile route to various azabicyclic compounds, including indolizidines and quinolizidines.^15^ Treatment of *N*-allyl 5-methyl-4-hexenamide (**2a**) with Rh(acac)(CO)_2_ (1 mol %), BIPHEPHOS (2 mol %), and pTSA (1.0 equiv) under an atmosphere of CO and H_2_ (1:1) in acetic acid at 60 °C yielded a bicyclized product as a major product, as well as a monocyclic intermediate. Subsequent treatment of the crude product with BF_3_·OEt_2_ in CH_2_Cl_2_ overnight furnished the complete formation of indolizidine **3a**. The procedure was performed on a 15 mmol scale with the pressure adjusted to 20 atm to afford indolizidine **3a** in 72% isolated yield as a 1.4:1 inseparable *cis*/*trans* mixture. The isopropenyl portion of **3a** was converted into an acetyl group using Nicolaou’s oxidative cleavage procedure^16^ to produce a separable mixture of *trans*- and *cis*-ketone, which could be epimerized to the more stable *trans*-ketone upon treatment within DBU in CH_2_Cl_2_. Thus, direct treatment of the oxidative cleavage product with DBU resulted in the clean formation of *trans*-ketone **4a** in 76% yield. With the optimized conditions in hand, treatment of 2-ethyl-substituted allylamide **2b** under the bicyclization conditions afforded an inseparable diastereomeric indolizidine mixture **3b1** in 47% yield (dr ∼2:1) and a single product **3b2** in 43% isolated yield. Subjecting indolizidine **3b2** to the oxidative cleavage protocol as stated above afforded a 55% yield of ketone **4b**, which was condensed with TsNHNH_2_ to crystalline product tosylhydrazone **5b**. The structure of **5b** was confirmed unequivocally by X-ray analysis [CCDC 1939866 (Scheme 1)], which discloses the *syn* relationship between H-6 and bridge H-8a. The result also substantiates the *syn* configuration of indolizidine **3b2**. Moreover, it was pleasing to learn that treatment of diastereomeric indolizidine mixture **3b1** under the oxidative cleavage conditions also gave the same ketone **4b** in 50% isolated yield, presumably because of the epimerization to the most stable isomer.

**Scheme 1:**
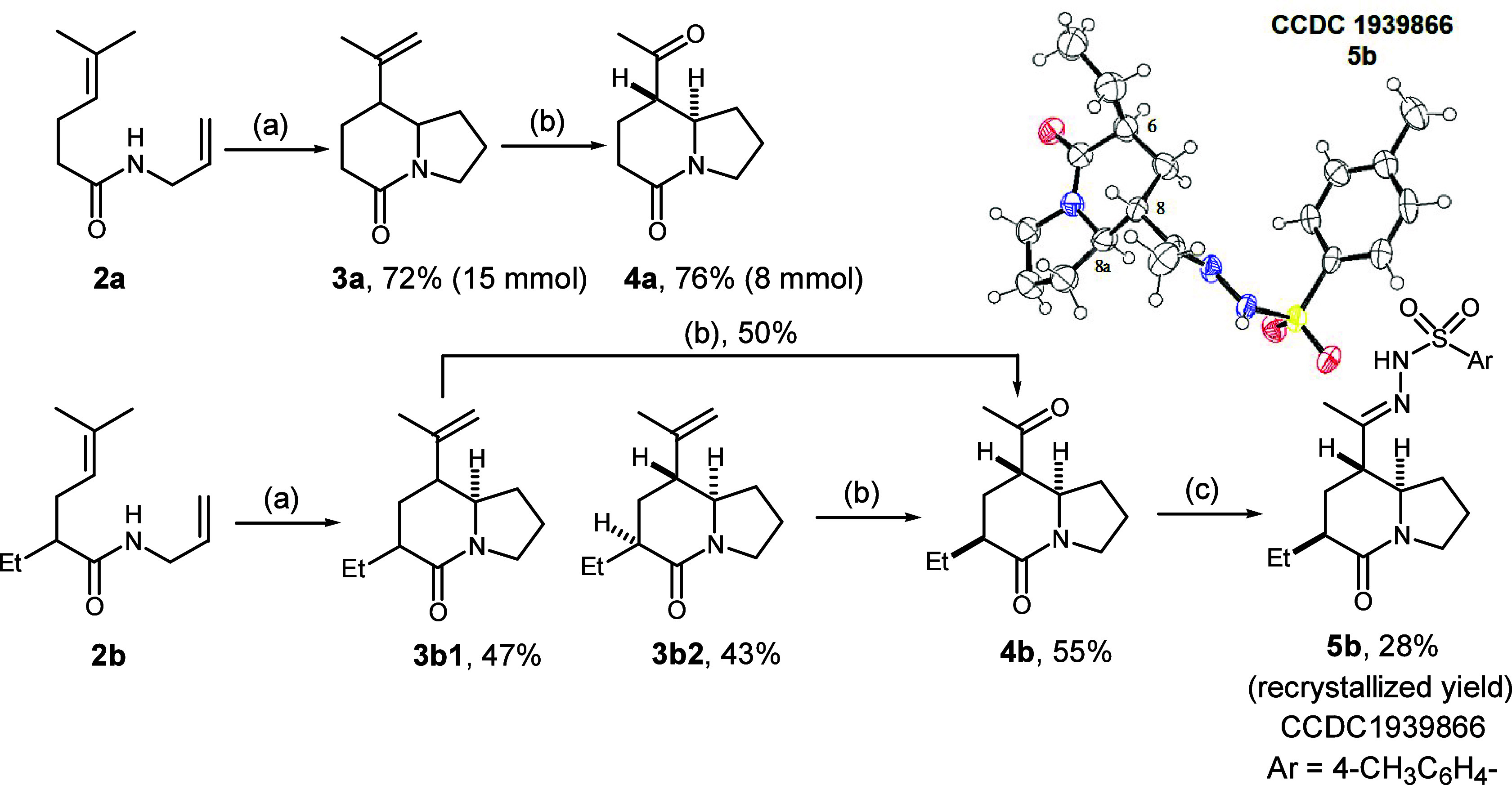
Synthesis of 8-Acetyl-5-oxoindolizidine **4** and X-ray Structure of Tosylhydrazone **5b**

A thioketal formation–reductive desulfurization sequence emerged as a facile procedure for converting the acetyl group into the desired ethyl group at position C-8. Several combinations of an acid (e.g., ZnCl_2_, BF_3_·OEt_2_, or pTSA) and a dithiol (ethylene or propylene) were examined, and ZnCl_2_-mediated 1,2-dithiolane formation was found to give the best result [95% yield (Scheme 2)]. Reduction of the 1,2-dithiolane moiety to the methylene group was easily accomplished by treating **6a** with Raney Ni in methanol, which afforded product **7a** in 95% isolated yield. However, we were finding low and unstable yields using the conditions for larger scale operations. Eventually, nickel boride,^17^ simply by treatment of NiCl_2_ with NaBH_4_ in ethanol, was found to be effective in this desulfurization to give a 97% yield of product **7a** on a 4.5 mmol scale. Using the synthesis shown above, dithiolane **6b** was obtained in 76% yield, and nickel boride reduction of **6b** gave a 65% yield of 6,8-diethyl product **7b**.

**Scheme 2:**
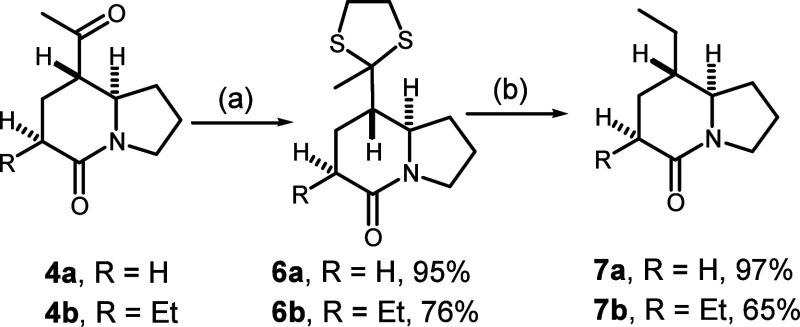
Synthesis of 8-Ethyl-5-oxoindolizidine

With a quite rigid 5-oxoindolizidine **7a** in hand, we investigated the base-mediated α-alkylation conditions for the introduction of the ethyl group at position C-6, and the results are summarized in Table 1.

**Table 1:**
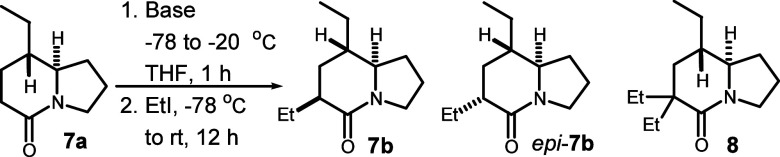
Base-Mediated α-Alkylation of 5-Oxoindolizidine **7a**

			yield (%)			
entry	EtI (equiv)	base	**7a**	**7b**	*epi***-7b**	**8**
1	1.5	*n*BuLi (1.1 equiv), DIPA (1.2 equiv)	97	ND^a^	ND^a^	ND^a^
2	2.0	*n*BuLi (2.0 equiv), DIPA (2.1 equiv)	33	36	17	ND^a^
3	2.0	LiHMDS (2.0 equiv)	36	38	23	ND^a^
4	2.5	*n*BuLi (2.0 equiv), DIPA (2.1 equiv)	ND^a^	29	12	34
5	2.1	*t*BuLi (2.0 equiv)	ND^a^	ND^a^	92	ND^a^

a Not detected.

Simple treatment with a slight excess of LDA (1.1 equiv) followed by addition of EtI (1.5 equiv) led primarily to recovered starting material **7a** (entry 1). The addition of more LDA (2.0 equiv) and EtI (2.0 equiv) resulted in the formation of two diastereomeric monoalkylated products, **7b** and *epi*-**7b**,^18^ as well as the recovery of starting material **7a** (entry 2). The switch to LiHMDS (2.0 equiv) gave results similar to those of entry 2 (entry 3). The addition of more EtI (2.5 equiv) yielded two monoalkylated products, accompanied by dialkylated product **8** in 34% yield (entry 4). Fortunately, lactam **7a** was successfully alkylated as a single product, *epi*-**7b**, in 92% isolated yield upon treatment with *tert*-BuLi (entry 5),^19^ probably due to a strong base-mediated irreversible deprotonation. The ^13^C NMR data of *epi*-**7b** were significantly different from those of **7b** but were in agreement with those reported in the literature^13^ (see the Supporting Information for comparison). The stereoselective strong base-mediated α-alkylation not only introduced a substituent at position C-6 but also provided a stereodivergent strategy for the construction of the diastereomeric 6-substituted 5-oxoindolizidine frameworks, such as alkaloid *epi*-223A.

To complete the synthesis of the targets, we focused on introduction of the propyl group at position C-5 by the amide activation–reductive alkylation strategy. Attempts to activate *epi*-**7b** with Lawesson’s reagent to form thiolactam were unsuccessful, because the conditions led to significant epimerization at position C-6. Instead, we found that Huang’s one-pot protocol for reductive alkylation of lactams,^20^ i.e., treatment within the Tf_2_O/DTBMP system followed by addition of *n*-PrMgCl and then LiAlH_4_ reduction, was effective for the conversion of indolizidine **7b** into final target alkaloid 223A (**1**) in 48% overall yield (Scheme 3). With the same procedure, *epi*-**7b** was converted into alkaloid 6-*epi*-223A (6-*epi*-**1**) in 53% yield. We were pleased to find that the reactions provided complete stereoselectivity for *syn* addition, i.e., the *cis* configuration between H-5 and bridged H-8a, in both reductions. The spectroscopic data of our synthetic samples 223A (**1**) and 6-*epi*-**1** were in agreement with those reported in the literature^4,6^ (see the Supporting Information for ^13^C NMR comparison).

**Scheme 3:**
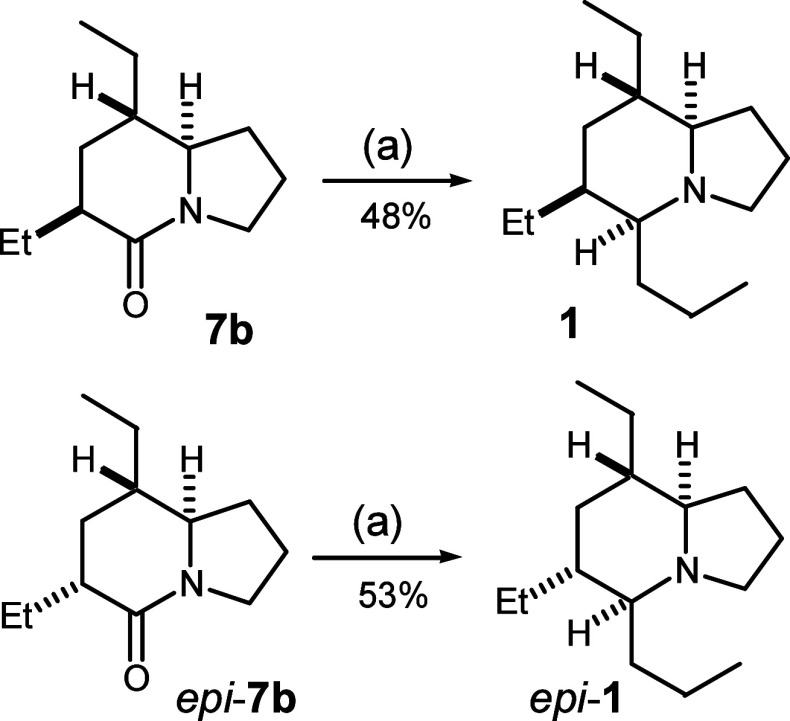
Synthesis of Alkaloid 223A and Alkaloid 6-*epi*-223A

To explain the observed *syn* selectivity in both diastereomeric products, we performed density functional theory (DFT) calculations to determine all four TS geometries in the last step during the reductive alkylation of lactams, i.e., LiAlH_4_ reduction of the corresponding iminium, which determined the C-5 configuration. The substituents at positions 5, 6, and 8 were replaced with a methyl group to simplify conformation issues and minimize the calculation cost. The four TS geometries are shown in Figure 2. The results show that TS_1_ is favored over TS_2_ by 3.4 kcal/mol, indicating the hydride addition prefers to proceed via “*syn* addition” to yield the *cis* configuration between H-5 and H-8a, found in alkaloid 223A. The “*syn* addition” constitutes an axial attack involving a chairlike TS_1_ and TS_3_, while the “*anti* addition” an equatorial attack with a boat-like TS_2_ and TS_4_. In addition, TS_3_ is favored over TS_4_ by 1.1 kcal/mol, suggesting the *syn* addition was preferred to give the same configuration in 6-*epi*-**1**.

**Figure 2:**
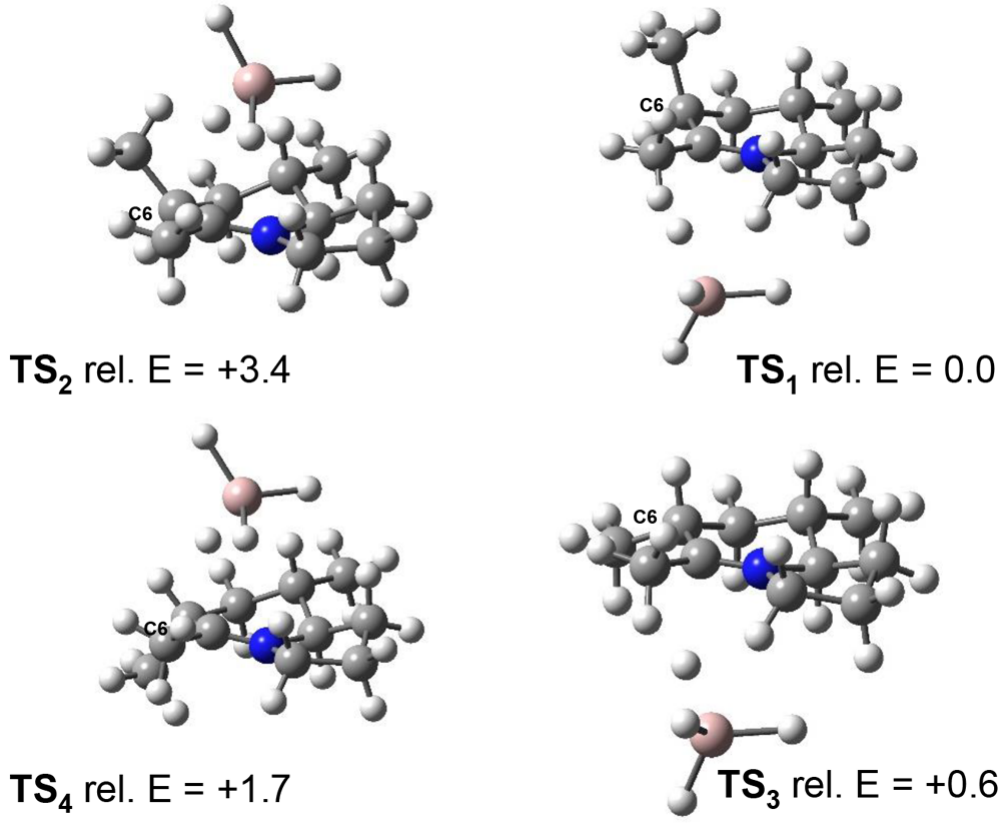
Four TS geometries in the reductive alkylation (units of kilocalories per mole).

In conclusion, we present a concise, divergent, and protecting group free syntheses of *Dendrobatid* alkaloid 223A (**1**) and 6-*epi*-223A (6-*epi*-**1**). The central step in these syntheses is the Rh-catalyzed hydroformylation double cyclization, which constructs the indolizidine ring system in one step from much simpler allylamide precursors. The relative rigid indolizidine framework constitutes the origin of the observed diastereoselectivity. The DFT calculations reveal that the addition of the *syn*-hydride in the Tf_2_O-activated reductive alkylation of the lactam involves the more stable TS, which may explain the observed *cis* configuration between H-5 and H-8a, in the formation of both 223A (**1**) and 6-*epi*-223A (*epi*-**1**). We are currently applying the methodology to indolizidine alkaloids, and the results will be disclosed in due course.

## Experimental Section

All reactions were performed under an argon atmosphere and in an anhydrous solvent, unless otherwise stated. An oil bath was used as the heat source. The solvents and reagents have been dried or refined according to the literature procedures. The reaction flasks were dried in a 110 °C oven, allowed to cool to room temperature in a desiccator with drying agents, and assembled under an argon atmosphere. Thin layer chromatography (TLC) was performed with ultraviolet light, an iodine chamber, 10% sulfuric acid, or a 10% PMA solution. The crude product were purified by flash column chromatography on silica gel to give the isolated yield. Infrared (IR) spectra were recorded on an ATR-FTIR apparatus. Melting points were recorded on a melting point apparatus. Single-crystal X-ray analysis was carried out with an X-ray diffractometer (Bruker D8 VENTURE), and the results have been reported to the Cambridge Crystallographic Data Centre to obtain the corresponding CCDC number. All NMR spectra, i.e., ^1^H, ^13^C, DEPT, gCOSY, gHSQC, and gHMBC, were recorded on a 400 or 600 MHz NMR spectrometer, which provided all necessary data for the full assignment of each compound. Chemical shifts (δ) are reported in parts per million using a residual undeuterated solvent as an internal standard. Coupling constants are in hertz. Mass spectra were recorded on a mass spectrometer with a magnetic sector as the mass analyzer, using electrospray ionization (ESI) or fast atom bombardment (FAB).

### General Procedure for the Reductive Alkylation of Lactams

To a dichloromethane (15 mL) solution of lactam **7b** (152 mg, 0.77 mmol, 1.0 equiv) and 2,6-di-*tert*-butyl-4-methylpyridine (DTBMP, 207 mg, 1.01 mmol, 1.3 equiv) in a 100 mL flask at −78 °C under argon was added triflic anhydride (0.17 mL, 1.01 mmol, 1.3 equiv). After the addition was completed, the cooling bath was removed so that the resulting solution was naturally warmed to 0 °C. After staying at the temperature for 30 min, the flask was again cooled to −78 °C. The *n*-PrMgCl solution (0.94 mL, 1 M in 2-MeTHF, 0.94 mmol, 1.2 equiv) was added via a syringe. The reaction mixture was allowed to be naturally warmed to room temperature for 16 h. LiAlH_4_ (86 mg, 2.3 mmol, 3.0 equiv) was added, and the solution was stirred for 3 h. Upon completion of the reaction as determined by TLC analysis, a NaOH solution (20%) was slowly added until a white gel formed. The reaction mixture was subjected to filtration with a short Celite column to give a filtrate. The filtrate was concentrated to the crude product. Purification of the crude product by flash chromatography on silica gel with an EtOAc/*n*-hex eluant gave the title product.

#### *rel-*(5*S,*6*S,*8*S,*8a*R*)-6,8-Diethyl-5-(1-propyl)indolizidine (**1**, alkaloid 223A)

83 mg, 48% yield; light yellow oil; *R*_*f*_ = 0.47 (1:3 EtOAc/*n*-hex); ^1^H NMR (400 MHz, 25 °C, CDCl_3_) δ 3.16–3.22 (m, 1H, H-3), 2.02–2.12 (m, 1H, H-8a), 1.84–1.98 (m, 3H, H-1, H-3, and H-7), 1.74–1.84 (m, 1H, H-2), 1.56–1.66 (m, 2H, H-2 and H-5), 1.36–1.54 (m, 7H, H-1, H-8, H-9, H-13 × 2, and H-14 × 2), 1.24–1.34 (m, 2H, H-6 and H-11), 1.16–1.24 (m, 1H, H-11), 0.96–1.08 (m, 1H, H-9), 0.80–0.92 (m, 10H, H-7, H-10 × 3, H-12 × 3, and H-15 × 3); ^13^C{^1^H} NMR (101 MHz, CDCl_3_) δ 71.4 (d), 66.8 (d), 52.0 (t), 37.7 (d), 37.2 (d), 33.4 (t), 32.4 (t), 29.2 (t), 25.9 (t), 20.4 (t), 19.1 (t), 18.3 (t), 14.5 (q), 12.4 (q), 11.0 (q); EI-HRMS (*m*/*z*) [M]^+^ calcd for C_15_H_29_N^+^ 223.2300, found 223.2301 (Δ = 0.4 ppm).

#### *rel-*(5*S,*6*R,*8*S,*8a*R*)-6,8-Diethyl-5-(1-propyl)indolizidine (*epi*-**1**)

99 mg, 53% yield; light yellow oil; *R*_*f*_ = 0.47 (1:3 EtOAc/*n*-hex); ^1^H NMR (400 MHz, 25 °C, CDCl_3_) δ 3.17 (brs, 1H, H-3), 1.82–1.98 (m, 3H), 1.62–1.78 (m, 4H), 1.30–1.58 (m, 8H), 1.18–1.28 (m, 1H, H-6), 0.96–1.10 (m, 2H), 0.82–0.92 (m, 9H), 0.59 (q, *J* = 12.0 Hz, 1H); ^13^C{^1^H} NMR (101 MHz, CDCl_3_) δ 69.9 (d), 67.4 (d), 52.0 (t), 42.4 (d), 40.0 (d), 35.3 (t), 33.0 (t), 28.9 (t), 26.0 (t), 24.7 (t), 20.8 (t), 17.9 (t), 14.8 (q), 11.1 (q, 2C); EI-HRMS (*m*/*z*) [M]^+^ calcd for C_15_H_29_N^+^ 223.2300, found 223.2308 (Δ = 3.6 ppm).

### General Procedure for the Preparation of *N*-Allylamide Substrates

To a mixture of the corresponding acid (10.0 mmol, 1.0 equiv), EDC (2.107 g, 11.0 mmol, 1.1 equiv), and HOBt·H_2_O (1.991 g, 13.0 mmol, 1.3 equiv) in CH_2_Cl_2_ (5.0 mL) in an ice bath was added the corresponding amine (10.0 mmol, 1.0 equiv) via a syringe. The reaction mixture was allowed to stir overnight (∼21 h) under argon. The reaction mixture was partitioned with CH_2_Cl_2_ (20 mL) and a saturated NaHCO_3_ solution (10 mL). The organic layer was washed with a saturated NH_4_Cl solution (10 mL). The aqueous solution was again extracted with CH_2_Cl_2_ (2 × 20 mL). The combined organic layers were washed with brine (10 mL), dried over anhydrous Na_2_SO_4_, and then concentrated under reduced pressure to give the crude product. Purification of the crude product by flash chromatography on silica gel, using an EtOAc/*n*-hexane eluant, afforded the corresponding amide in high yield.

#### *N*-Allyl-5-methyl-4-hexenamide (**2a**)

1.622 g, 97% yield; colorless oil; *R*_*f*_ = 0.60 (1:1 EtOAc/*n*-hex); IR (cm^–1^, film) ν̅_max_ 3317, 3084, 2926, 2858, 1646, 1541, 1435, 1376, 1266, 988, 919, 699; ^1^H NMR (400 MHz, CDCl_3_) δ 6.05 (brs, 1H), 5.73–5.82 (m, 1H), 5.08–5.14 (m, 3H), 3.81 (q, *J* = 6.0 Hz, 2H), 2.27 (q, *J* = 7.2 Hz, 2H), 2.16–2.20 (m, 2H), 1.63 (s, 3H), 1.57 (s, 3H); ^13^C{^1^H} NMR (101 MHz, CDCl_3_) δ 172.7 (s), 134.2 (d), 132.9 (s), 112.7 (d), 115.9 (t), 41.7 (t), 36.5 (t), 25.5 (q), 24.2 (t), 17.6 (q); EI-HRMS (*m*/*z*) [M]^+^ calcd for C_10_H_17_NO^+^ 167.1310, found 167.1314 (Δ = 2.4 ppm). The spectral data are in agreement with those reported in the literature.^15^

#### *N*-Allyl-2-ethyl-5-methyl-4-hexenamide (**2b**)

1.776 g, 91% yield; colorless oil,; *R*_*f*_ = 0.60 (1:1 EtOAc/*n*-Hex); IR (cm^–1^, film) ν̅_max_ 3290, 3083, 2964, 1657, 1552, 1456, 1380, 1259, 1229, 987, 917, 827, 698; ^1^H NMR (400 MHz, CDCl_3_) δ 5.75–5.84 (m, 1H), 5.72 (brs, 1H), 5.03–5.17 (m, 3H), 3.80–3.91 (m, 2H), 2.20–2.26 (m, 1H), 2.09–2.16 (m, 1H), 1.91–1.98 (m, 1H), 1.65 (s, 3H), 1.54–1.64 (m, 1H), 1.57 (s, 3H), 1.43–1.49 (m, 1H), 0.87 (t, *J* = 7.6 Hz, 3H); ^13^C{^1^H} NMR (101 MHz, CDCl_3_) δ 175.3 (s), 134.5 (d), 133.4 (s), 121.7 (d), 115.9 (t), 49.8 (d), 41.6 (t), 31.2 (t), 25.7 (q), 25.4 (t), 17.7 (q), 12.1 (q); EI-HRMS (*m*/*z*) [M]^+^ calcd for C_12_H_21_NO^+^ 195.1623, found 195.1622 (Δ = −0.5 ppm).

### General Procedure for Domino Double Cyclization

Rh(acac)(CO)_2_ (2.6 mg, 1.0 mol %) and BIPHEPHOS (15.8 mg, 2.0 mol %) were dissolved in AcOH (1 mL) under argon. The resulting catalyst solution was degassed by a freezing–thawing procedure at least three times. *N*-Allyl amide (**2**, 1 mmol, 1.0 equiv) and pTSA (1.0 equiv) were placed in a 50 mL flask. The catalyst solution was transferred to the reaction flask containing the substrate by a pipet, and the total volume was adjusted to 20 mL with AcOH (0.05 M). The reaction flask was placed in a 300 mL stainless steel autoclave and pressurized with CO (2.2 atm) followed by H_2_ (2.2 atm). The pressure for CO and H_2_ was increased to 10 atm each as the 15 mmol substrate was used. Such an operation guaranteed the completion of the reaction. The reaction mixture was stirred at 60 °C for 16–20 h. Upon completion of the reaction as determined by TLC analysis, the gas was carefully released in a good ventilated hood and the reaction mixture was concentrated under reduced pressure to give the crude residue.

The residue was diluted with CH_2_Cl_2_ (∼0.2 M) and then added with BF_3_·OEt_2_ (2.5 equiv) to an ice bath. The solution was then stirred at room temperature for 16–20 h. Upon completion of the reaction, saturated sodium bicarbonate (amount approximately equal to that of CH_2_Cl_2_) was added. After separation of the organic layer, the aqueous layer was again extracted with CH_2_Cl_2_ (four times). The combined organic layers were dried over anhydrous Na_2_SO_4_, filtered, and then concentrated under reduced pressure to give the crude product. The crude product was purified by flash chromatography on silica gel using a MeOH/CH_2_Cl_2_ or EtOAc/*n*-hexane eluant to give the product.

#### 8-(Propen-2-yl)-5-oxoindolizidine (**3a**)

129 mg, 72% yield (1.4:1 *cis*:*trans*); yellow oil; *R*_*f*_ = 0.24 (1:20 MeOH/CH_2_Cl_2_); ^1^H NMR (400 MHz, 25 °C, CDCl_3_) δ 4.57–4.89 (m, 4H), 3.66 (ddd, *J* = 6.0, 6.0, 10.8, Hz, 1H), 3.44–3.60 (m, 4H), 3.33 (ddd, *J* = 4.8, 10.2, 10.2 Hz, 1H), 2.59 (q, *J* = 4.8 Hz, 1H), 2.48–2.52 (m, 1H), 2.31–2.43 (m, 3H), 1.84–2.09 (m, 6H), 1.71–1.81 (m, 12H), 1.34–1.39 (m, 1H); ^13^C{^1^H} NMR (101 MHz, CDCl_3_) δ 169.3 (s), 168.7 (s), 145.0 (s), 143.1 (s), 113.0 (t), 112.1 (t), 61.8 (d), 60.5 (d), 48.4 (d), 45.2 (t), 44.7 (t), 41.0 (d), 32.3 (t), 31.3 (t), 29.1 (t), 28.3 (t), 27.2 (t), 24.8 (t), 23.6 (q), 21.9 (t), 21.6 (t), 20.0 (q). The spectral data are in agreement with those reported in the literature.^15^

#### 6-Ethyl-8-(2-propenyl)-5-oxoindolizidine (**3b1**)

97 mg, 47% yield (dr 2:1); yellow oil; *R*_*f*_ = 0.55 (pure EtOAc); IR (cm^–1^, film) ν̅_max_ 2967, 2967, 2936, 2876, 1631, 1379, 1334, 1275, 1180, 749; ^1^H NMR (400 MHz, 25 °C, CDCl_3_) δ 4.80 (s, 1H), 4.68 (s, 1H), 3.80 (dd, *J* = 11.7, 8.2 Hz, 1H), 3.62–3.74 (m, 1H), 3.49–3.61 (m, 1H), 3.38 (dd, *J* = 14.7, 5.8 Hz, 1H), 3.24 (d, *J* = 10.1 Hz, 1H), 3.01–3.22 (m, 1H), 2.74 (dd, *J* = 14.4, 7.1 Hz, 1H), 1.90–2.18 (m, 2H), 1.73–1.90 (m, 2H), 1.59–1.68 (m, 3H), 1.29–1.59 (m, 2H), 0.81–0.99 (m, 3H); ^13^C{^1^H} NMR (101 MHz, CDCl_3_) δ 172.3 (s, 2C), 145.5 (s, 2C), 112.7 (t, 2C), 59.9 (d), 58.4 (d), 44.2 (t), 43.2 (t), 43.1 (d), 42.7 (d), 42.2 (d), 41.6 (d), 29.2 (t), 28.1 (t), 26.3 (t), 25.6 (t), 22.9 (t), 22.7 (t), 22.6 (t), 21.9 (t), 21.6 (q), 21.4 (q), 11.9 (q), 11.8 (q); EI-HRMS (*m*/*z*) [M]^+^ calcd for C_13_H_21_NO^+^ 207.1623, found 207.1621 (Δ = −0.9 ppm).

#### *rel-*(6*S,*8*R,*8a*R*)-6-Ethyl-8-(2-propenyl)-5-oxoindolizidine (**3b2**)

89 mg, 43% yield; yellow oil; *R*_*f*_ = 0.25 (pure EtOAc); IR (cm^–1^, film) ν̅_max_ 3439, 2966, 2936, 2877, 1729, 1621, 1459, 1375, 1250, 1057, 893, 522; ^1^H NMR (400 MHz, 25 °C, CDCl_3_) δ 4.83 (s, 1H), 4.81 (s, 1H), 3.53–3.60 (m, 1H), 3.44–3.50 (m, 1H), 3.60 (ddd, *J* = 5.2, 10.4, 10.4 Hz, 1H), 2.26–2.34 (m, 1H), 2.01–2.07 (m, 2H), 1.82–1.98 (m, 3H), 1.68 (s, 3H), 1.63–1.77 (m), 1.34–1.52 (m, 2H), 0.98 (s, *J* = 7.6 Hz, 3H); ^13^C{^1^H} NMR (101 MHz, CDCl_3_) δ 171.8 (s), 145.1 (s), 112.0 (t), 61.4 (d), 45.2 (d), 45.1 (t), 41.7 (d), 32.2 (t), 30.7 (t), 25.4 (t), 22.1 (t), 19.9 (q), 12.3 (q); EI-HRMS (*m*/*z*) [M]^+^ calcd for C_13_H_21_NO^+^ 207.1623, found 207.1621 (Δ = −0.9 ppm).

### General Procedure for Oxidative Cleavage

To the mixed solution (0.1 M, 18 mL) of acetone and water (9:1) of the alkene substrate (**3a**, 323 mg, 1.80 mmol, 1.0 equiv) under argon were added 2,6-dimethylpyridine (0.63 mL, 3.0 equiv), an *N*-methyl morpholine oxide aqueous solution (50%, 2 mL, 5 equiv), and OsO_4_ (4.6 mg, 1 mol %). The solution was allowed to stir overnight (∼16 h) at 60 °C. The reaction was monitored by TLC in which the *R*_*f*_ value of the starting material alkene is ∼0.24 (1:20 MeOH/CH_2_Cl_2_), while the *R*_*f*_ value of the product diol is ∼0.10 in the same eluent. Upon completion of the reaction, PhI(OAc)_2_ (696 mg, 1.2 equiv) was added, the mixture stirred overnight (∼20 h) at 60 °C. TLC analysis was used to follow the reaction. After the volatiles had evaporated, the resulting reaction mixture was diluted with CH_2_Cl_2_ (20 mL) and washed with a saturated Na_2_S_2_O_4_ solution (30 mL). The aqueous layer was extracted three times with CH_2_Cl_2_ (20 mL each). The organic layer was washed with brine (10 mL), dried over anhydrous Na_2_SO_4_, and then concentrated under reduced pressure to give the crude product.

To the dichloromethane solution of the crude product (∼0.1 M) in an ice bath was added DBU (0.30 mL, 1.1 equiv). The resulting solution was stirred overnight (∼16 h) and then washed with a saturated NH_4_Cl solution. After separation from the organic layer, the resulting aqueous solution was extracted three times with CH_2_Cl_2_. The organic layer was washed with brine (10 mL), dried over anhydrous Na_2_SO_4_, and then concentrated under reduced pressure to give the crude product. Purification of the crude product by flash chromatography on silica gel, using ethyl acetate and *n*-hexane as the eluant, gave the two title products.

#### *trans*-8-Acetyl-5-oxoindolizidine (**4a**)

248 mg, 76% yield; yellow oil; *R*_*f*_ = 0.37 (1:20 MeOH/CH_2_Cl_2_); IR (cm^–1^, film) ν̅_max_ 3349, 2927, 1632, 1456, 1261, 1056, 749, 700; ^1^H NMR (400 MHz, 25 °C, CDCl_3_) δ 3.50–3.58 (m, 2H), 3.42 (t, *J* = 11.6 Hz, 1H), 2.28–2.56 (m, 3H), 2.22 (s, 3H), 2.04–2.18 (m, 2H), 1.90–1.98 (m, 1H), 1.71–1.79 (m, 2H), 1.29–1.38 (m, 1H); ^13^C{^1^H} NMR (101 MHz, CDCl_3_) δ 208.3 (s), 167.8 (s), 59.5 (d), 53.1 (d), 44.7 (t), 32.0 (t), 30.7 (t), 29.4 (q), 24.9 (t), 22.0 (t); EI-HRMS (*m*/*z*) [M]^+^ calcd for C_10_H_15_NO_2_^+^ 181.1103, found 181.1100 (Δ = −1.7 ppm). The spectral data are in agreement with those reported in the literature.^15^

#### *rel-*(6*S,*8*S,*8a*R*)-6-Ethyl-8-acetyl-5-oxoindolizidine (**4b**)

111 mg, 50% yield; yellow oil; *R*_*f*_ = 0.25 (pure EtOAc); IR (cm^–1^, film) ν̅_max_ 3448, 2930, 2877, 1709, 1625, 1459, 1174, 732, 600; ^1^H NMR (400 MHz, 25 °C, CDCl_3_) δ 3.55 (ddd, *J* = 4.8, 11.2, 11.2 Hz, 1H), 3.32–3.48 (m, 2H), 2.46 (ddd, *J* = 4.8, 11.2, 11.2 Hz, 1H), 2.23–2.32 (m, 1H), 2.16 (s, 3H), 2.10–2.18 (m, 1H), 1.80–1.98 (m, 4H), 1.66–1.80 (m, 1H), 1.28–1.50 (m, 2H), 0.96 (t, *J* = 7.4 Hz, 3H); ^13^C{^1^H} NMR (101 MHz, CDCl_3_) δ 208.5 (s), 171.0 (s), 58.8 (d), 50.7 (d), 44.6 (t), 41.1 (d), 32.2 (t), 29.3 (q), 28.5 (t), 25.0 (t), 22.3 (t), 12.1 (q); EI-HRMS (*m*/*z*) [M]^+^ calcd for C_12_H_19_NO_2_^+^ 209.1416, found 209.1413 (Δ = −1.4 ppm).

### Tosylhydrazone Formation

#### *rel-*(6*S,*8*S,*8a*R*)-6-Ethyl-8-acetyl-5-oxoindolizidine Tosylhydrazone (**5b**, CCDC 1939886)

To a methanol solution (4 mL) of methylketone **4b** (228 mg, 1.09 mmol, 1.0 equiv) under argon was added tosylhydrazide (223 mg, 1.19 mmol, 1.1 equiv). The solution was allowed to stir under reflux for 1 h. Upon completion of the reaction, the mixture was allowed to stand to cool slowly to room temperature. A large quantity of white crystals separated during that time. The resulting white crystal was isolated by being carefully washed with cold methanol and dried under vacuum. The crystal was used for X-ray analysis (131 mg, 0.31 mmol, 28%): mp 214–216 °C; *R*_*f*_ = 0.20 (pure EtOAc); ^1^H NMR (400 MHz, 25 °C, CDCl_3_) δ 7.81 (d, *J* = 8.0 Hz, 2H), 7.55 (brs, 1H, NH), 7.31 (d, *J* = 8.4 Hz, 2H), 3.28–3.85 (m, 3H), 2.43 (s, 3H), 2.24–2.29 (m, 1H), 2.20 (ddd, *J* = 4.8, 10.8, 10.8 Hz, 1H), 1.91–1.99 (m, 1H), 1.81 (s, 3H), 1.71–1.87 (m, 4H), 1.60–1.70 (m, 1H), 1.23–1.41 (m, 1H), 1.18 (dddd, *J* = 3.6, 8.4, 8.4, 8.4 Hz, 1H), 0.95 (t, *J* = 7.2 Hz, 3H); ^13^C{^1^H} NMR (101 MHz, CDCl_3_) δ 171.3 (s), 156.4 (s), 144.2 (s), 135.2 (s), 129.4 (d), 128.1 (d), 60.0 (d), 46.1 (d), 45.0 (t), 41.4 (d), 32.0 (t), 29.6 (t), 25.2 (t), 22.3 (t), 21.5 (q), 15.1 (q), 12.2 (q); EI-HRMS (*m*/*z*) [M]^+^ calcd for [C_19_H_27_N_3_O_3_S·Na]^+^ 400.1671, found 400.1664 (Δ = −1.7 ppm).

### General Procedure for the Formation of 1,3-Dithiolane

To a toluene (30 mL, 0.2 M) solution of methyl ketone **4a** (989 mg, 5.45 mmol, 1.00 equiv) and anhydrous ZnCl_2_ powder (1.111 g, 8.15 mmol, 1.5 equiv) in a 100 mL flask under argon was added 1,2-ethanedithiol (1.37 mL, 16.3 mmol, 3.0 equiv) via a syringe. After the flask had been fitted with a Dean-Stark trap with a water-cooled condenser, the solution was heated under reflux to remove water. Upon completion of the reaction as monitored by TLC analysis, the mixture was cooled, followed by the addition of a saturated NH_4_Cl solution (20 mL). The solution was stirred for 1 h. The addition of a small quantity of water might dissolve the precipitate. After separation of the organic layer, the aqueous layer was extracted with EtOAc (4 × 20 mL). The combined organic extracts were dried over Na_2_SO_4_. After removal of the solid dehydrating agent, the organic layer was concentrated under reduced pressure to give the crude product. Purification of the crude product by flash chromatography on silica gel, with an EtOAc/*n*-hex eluant, gave the title products.

#### *trans-*8-(2-Methyl-1,3-dithiolan-2-yl)-5-oxoindolizidine (**6a**)

1.335 g, 95% yield; yellow oil; *R*_*f*_ = 0.25 (pure EtOAc); IR (cm^–1^, film) ν̅_max_ 2962, 2929, 2874, 1633, 1457, 1378, 1245, 1108, 1050, 749, 701, 609; ^1^H NMR (400 MHz, 25 °C, CDCl_3_) δ 3.40–3.52 (m, 5H), 3.21–3.29 (m, 2H), 2.38–2.43 (m, 1H), 2.23–2.36 (m, 2H), 2.13–2.19 (m, 2H), 1.86–1.92 (m, 2H), 1.64 (s, 3H), 1.59–1.72 (m, 2H); ^13^C{^1^H} NMR (101 MHz, CDCl_3_) δ 168.5 (s), 69.1 (s), 62.1 (d), 51.6 (d), 44.4 (t), 39.9 (t), 38.1 (t), 33.9 (t), 31.4 (t), 29.9 (q), 27.4 (t), 22.3 (t); EI-HRMS (*m*/*z*) [M]^+^ calcd for C_12_H_19_NOS_2_^+^ 257.0908, found 257.0913 (Δ = 1.9 ppm).

#### *rel-*(6*S,*8*S,*8a*R*)-6-Ethyl-8-(2-methyl-1,3-dithiolan-2-yl)-5-oxoindolizidine (**6b**)

993 mg, 76% yield; light yellow oil; *R*_*f*_ = 0.37 (pure EtOAc); IR (cm^–1^, film) ν̅_max_ 3449, 2962, 2962, 2929, 2874, 1633, 1457, 1378, 1245, 1108, 1050, 749, 701, 609; ^1^H NMR (400 MHz, 25 °C, CDCl_3_) δ 3.30–3.42 (m, 2H), 3.24–3.36 (m, 3H), 3.13–3.24 (m, 2H), 2.30–2.36 (m, 1H), 2.22–2.28 (m, 1H), 2.02 (ddd, *J* = 4.0, 10.0, 10.0 Hz, 1H), 1.92–2.01 (m, 1H), 1.77–1.92 (m, 3H), 1.69 (s, 3H), 1.55–1.68 (m, 2H), 1.27–1.39 (m, 1H), 0.93 (t, *J* = 7.6 Hz, 3H); ^13^C{^1^H} NMR (101 MHz, CDCl_3_) δ 171.9 (s), 69.6 (s), 61.4 (d), 48.4 (d), 44.3 (t), 41.7 (d, C-6), 39.7 (t), 38.2 (t), 34.4 (t), 31.1 (t), 30.4 (q), 24.3 (t), 22.6 (t), 12.0 (q); EI-HRMS (*m*/*z*) [M]^+^ calcd for C_14_H_23_NOS_2_^+^ 285.1221, found 285.1226 (Δ = 1.7 ppm).

### General Procedure for Nickel Boride Reduction of 1,3-Dithiolane

To an ethanol (50 mL) solution of dithiolane **6a** (849 mg, 3.29 mmol, 1.00 equiv) and NiCl_2_·6H_2_O powder (7.82 g, 32.9 mmol, 10.0 equiv) in a 100 mL flask at −50 °C under argon was added NaBH_4_ (2.49 g, 65.8 mmol, 20.0 equiv) in several portions. This addition produced a large volume of gas, and the solution turned gray. After the addition was completed, the reaction mixture was stirred at room temperature. Upon completion of the reaction as monitored by TLC analysis, the mixture was subjected to filtration with a Büchner funnel to remove the dark gray solid. The filtrate was concentrated to the crude residue that was partitioned with water (30 mL) and EtOAc (30 mL). After separation of the organic layer, the aqueous layer was extracted with EtOAc (3 × 30 mL). The combined organic extracts were dried over Na_2_SO_4_. After removal of the solid dehydrating agent, the organic layer was concentrated under reduced pressure to give the crude product. Purification of the crude product by flash chromatography on silica gel, with an EtOAc/*n*-hex eluant, gave the title products.

#### *trans*-8-Ethyl-5-oxoindolizidine (**7a**)

534 mg, 97% yield; yellow oil; *R*_*f*_ = 0.22 (pure EtOAc); IR (cm^–1^, film) ν̅_max_ 3417, 2963, 2933, 2877, 1599, 1493, 1459, 1345, 1324, 1051, 700; ^1^H NMR (400 MHz, 25 °C, CDCl_3_) δ 3.48–3.53 (m, 1H), 3.38–3.44 (m, 1H), 3.04 (ddd, *J* = 4.8, 10.0, 10.0 Hz, 1H), 2.44 (dd, *J* = 6.4, 18.0 Hz, 1H), 2.26 (ddd, *J* = 6.8, 11.6, 18.4 Hz, 1H), 2.13 (ddd, *J* = 6.0, 6.0, 11.6 Hz, 1H), 1.86–1.96 (m, 2H), 1.63–1.74 (m, 1H), 1.51–1.59 (m, 1H), 1.26–1.40 (m, 2H), 1.12–1.24 (m, 2H), 0.91 (t, *J* = 7.2 Hz, 3H); ^13^C{^1^H} NMR (101 MHz, CDCl_3_) δ 169.3 (s), 63.9 (d), 45.1 (t), 41.8 (d), 32.3 (t), 31.4 (t), 26.3 (t), 25.2 (t), 22.2 (t), 11.0 (q); EI-HRMS (*m*/*z*) [M]^+^ calcd for C_10_H_17_NO^+^ 167.1310, found 167.1303 (Δ = −4.2 ppm).

#### *rel-*(6*S,*8*S,*8a*R*)-6,8-Diethyl-5-oxoindolizidine (**7b**)

442 mg, 65% yield; light yellow oil; *R*_*f*_ = 0.40 (pure EtOAc); IR (cm^–1^, film) ν̅_max_ 3417, 2963, 2931, 2876, 1711, 1634, 1458, 1379, 1304, 1258, 1124, 749; ^1^H NMR (400 MHz, 25 °C, CDCl_3_) δ 3.38–3.52 (m, 2H), 3.03 (ddd, *J* = 5.2, 10.0, 10.0 Hz, 1H), 2.19–2.28 (m, 1H), 2.18–2.26 (m, 1H), 1.78–1.94 (m, 3H, H-2), 1.62–1.76 (m, 1H), 1.48–1.60 (m, 1H), 1.22–1.48 (m, 4H), 1.11–1.22 (m, 1H, H-9), 0.94 (t, *J* = 7.2 Hz, 3H), 0.92 (t, *J* = 7.2 Hz, 3H); ^13^C{^1^H} NMR (101 MHz, CDCl_3_) δ 172.3 (s), 63.6 (d), 45.0 (t), 41.7 (d), 38.6 (d), 32.4 (t), 30.0 (t), 25.5 (t), 25.3 (t), 22.3 (t), 12.3 (q), 11.1 (q); EI-HRMS (*m*/*z*) [M]^+^ calcd for C_12_H_21_NO^+^ 195.1623, found 195.1632 (Δ = 4.6 ppm).

### *tert*-BuLi-Mediated Alkylation

#### *rel-*(6*R,*8*S,*8a*R*)-6,8-Diethyl-5-oxoindolizidine (*epi*-**7b**)

To a THF solution (50 mL) of lactam **7a** (272 mg, 1.63 mmol, 1.0 equiv) at −78 °C was slowly added a *tert*-BuLi solution (1.8 M in pentane, 1.78 mL, 3.26 mmol, 2.0 equiv) via a syringe. After the addition of the base had been completed, the bath was removed, and the solution was warmed to 0 °C and then stirred in an ice bath for 30 min. The reaction mixture was again cooled to −78 °C, and EtI (0.275 mL, 3.42 mmol, 2.1 equiv) was added via a syringe. The reaction mixture was warmed gradually to room temperature in 16 h. The reaction was quenched by the addition of a saturated NH_4_Cl (10 mL) solution and EtOAc (30 mL). After separation of the organic layer, the aqueous layer was extracted with EtOAc (3 × 20 mL). The combined organic layers were dried over Na_2_SO_4_. After removal of the solid dehydrating agent, the organic layer was concentrated under reduced pressure to give the crude product. Purification of the crude product by flash chromatography on silica gel, with an EtOAc/*n*-hex eluant, gave a colorless oil as the title product (291 mg, 1.49 mmol, 92%): *R*_*f*_ = 0.34 (pure EtOAc); IR (cm^–1^, film) ν̅_max_ 3483, 2969, 2939, 2879, 2579, 1714, 1609, 1460, 1382, 1192, 736; ^1^H NMR (400 MHz, 25 °C, CDCl_3_) δ 3.50–3.58 (m, 1H), 3.41 (t, *J* = 10.4 Hz, 1H), 3.06 (ddd, *J* = 5.2, 10.4, 10.4 Hz, 1H), 2.16–2.22 (m, 1H), 2.09–2.14 (m, 1H), 1.89–2.05 (m, 3H), 1.64–1.73 (m, 1H), 1.45–1.60 (m, 2H), 1.35 (dddd, *J* = 7.0, 12.0, 12.0, 12.0 Hz, 1H), 1.12–1.27 (m, 2H), 1.05 (q, *J* = 12.0 Hz, 1H), 0.94 (t, *J* = 6.8 Hz, 3H), 0.90 (t, *J* = 7.2 Hz, 3H); ^13^C{^1^H} NMR (101 MHz, CDCl_3_) δ 171.4 (s), 63.7 (d), 45.1 (t), 42.4 (d), 41.3 (d), 32.1 (t), 31.7 (t), 25.1 (t), 24.9 (t), 22.1 (t), 10.8 (q), 10.7 (q); ESI-HRMS (*m*/*z*) [M + H]^+^ calcd for [C_12_H_21_NO·H]^+^ 196.1701, found 196.1693 (Δ = −4.0 ppm).

#### *rel-*(8*S,*8a*R*)-6,6,8-Triethyl-5-oxoindolizidine (**8**)

Colorless oil; *R*_*f*_ = 0.44 (pure EtOAc); ^1^H NMR (400 MHz, 25 °C, CDCl_3_) δ 3.38–3.53 (m, 2H), 3.01 (ddd, *J* = 5.2, 10.8, 10.8 Hz, 1H), 2.11 (quintet, *J* = 6.0 Hz, 1H), 1.87–1.93 (m, 1H), 1.61–1.78 (m, 4H), 1.44–1.58 (m, 3H), 1.24–1.38 (m, 3H), 1.06–1.16 (m, 1H), 0.98 (t, *J* = 7.2 Hz, 3H), 0.80 (t, *J* = 7.6 Hz, 3H), 0.78 (t, *J* = 7.6 Hz, 3H); ^13^C{^1^H} NMR (101 MHz, CDCl_3_) δ 174.0 (s), 64.0 (d), 42.7 (s), 45.3 (t), 39.7 (d, C-8), 34.7 (t), 32.6 (t), 32.5 (t), 31.7 (t), 25.3 (t), 22.4 (t), 11.0 (q), 9.2 (q), 8.8 (q); EI-HRMS (*m*/*z*) [M]^+^ calcd for C_14_H_25_NO^+^ 223.1936, found 223.1941 (Δ = 2.2 ppm).

## Data Availability

The data underlying this study are available in the published article and its Supporting Information.

## Supporting Information Available

(^1^H and ^13^C NMR spectra of all compounds and all calculated coordinates of the TS geometries (PDF)
