# Supplementary material for: Stereodivergent Synthesis of Alkaloid (±)-223A and (±)-6-epi-223A via Rh-Catalyzed Hydroformylation Double Cyclization
Source: J Org Chem. 2024 Mar 8;89(7):5091–7. doi: 10.1021/acs.joc.3c02366 (PMC11002921; doi:10.1021/acs.joc.3c02366)
Supplement: Supplementary file 1 — jo3c02366_si_001.pdf [file jo3c02366_si_001.pdf]

Supporting Information

Stereodivergent Synthesis of Alkaloid (±)-223A and (±)-6-*epi*-223A via Rh-catalyzed hydroformylation Double Cyclization

Wen-Wei Huang, Jui-Teng Cheng,[#] Wei-Ting Hsiao[#] and Wen-Hua Chiou*

Department of Chemistry, National Chung Hsing University, Taichung 402202, Taiwan, R.O.C.

e-mail: wchiou@dragon.nchu.edu.tw

Table of Contents

^{13}C-NMR comparison of alkaloid 223A (**1**) and alkaloid 6-*epi*-223A (6-*epi*-**1**) with the literature SI-1~4

^{13}C-NMR comparison of lactam *epi*-**7b** with the literature SI-5~7

Crystal data and structure refinement and ORTEP Drawing of compound **5b** SI-8~9

Computational Details and the TS geometry SI-10~14

^{1}H-NMR and ^{13}C-NMR spectra of all compounds SI-15~59

References SI-60

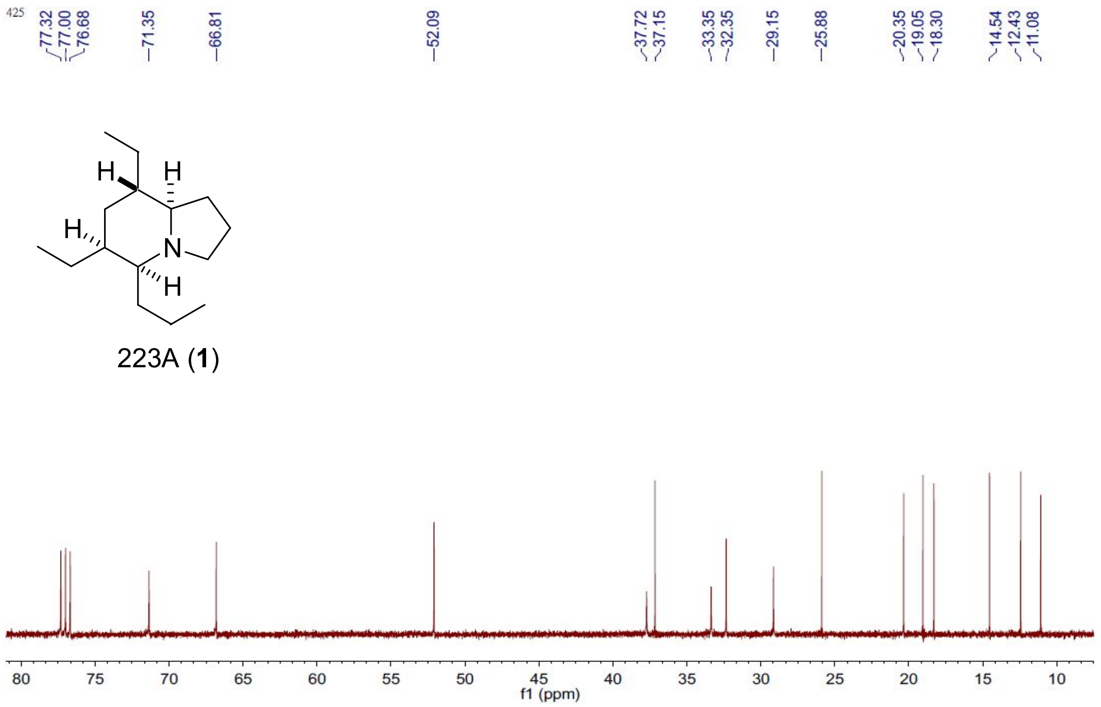

INDEX	FREQUENCY	PPM	HEIGHT
1	5841.541	77.421	53.0
2	5809.800	77.000	52.1
3	5778.060	76.579	49.7
4	5382.528	71.337	17.6
5	5041.931	66.823	23.9
6	3933.465	52.132	18.6
7	2856.739	37.862	19.6
8	2812.791	37.279	15.5
9	2528.350	33.509	18.5
10	2451.441	32.490	17.8
11	2209.727	29.287	14.5
12	1961.909	26.002	18.3
13	1545.624	20.485	15.7
14	1446.741	19.174	21.5
15	1391.806	18.446	15.5
16	1107.365	14.676	18.6
17	947.443	12.557	18.6
18	847.339	11.230	17.2

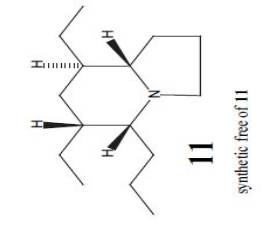

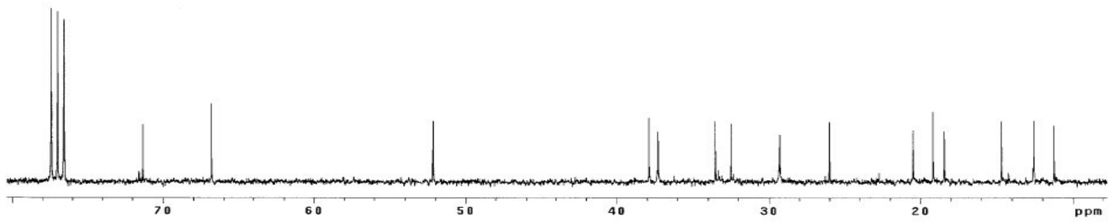

Figure S1. Comparison of the ^{13}C-NMR spectra of 223A (**1**) in $CDCl_3$ with the literature spectrum (lower, Toyooka et al. *Org. Lett.* **2002**, *11*, 1715-1717).[1]

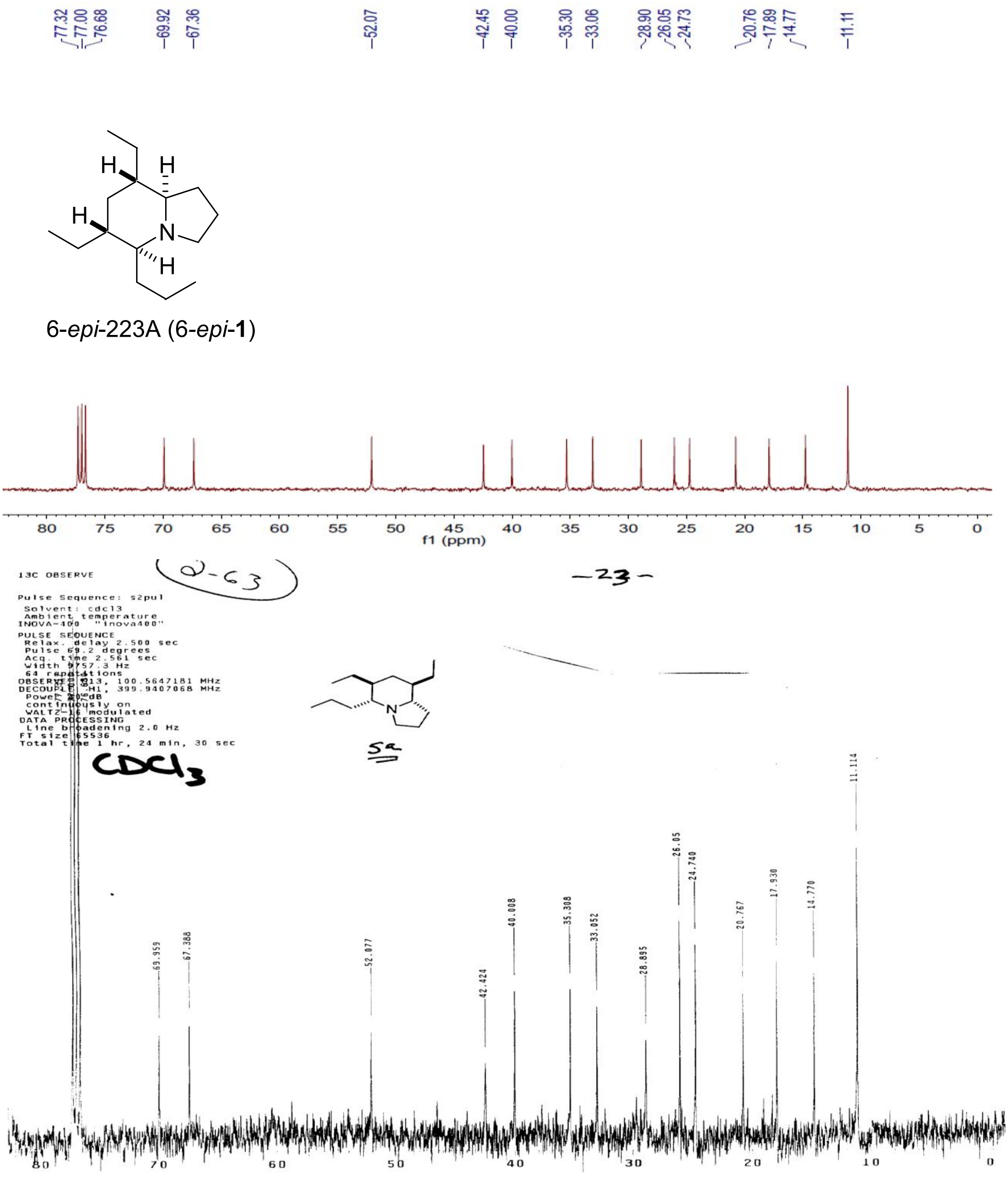

Figure S2. Comparison of the ^{13}C-NMR spectra of 6-*epi*-223A in $CDCl_3$ with the literature spectrum (lower, Padwa et al. *J. Org. Chem.* **2003,** *68*, 4371-4381). [2]

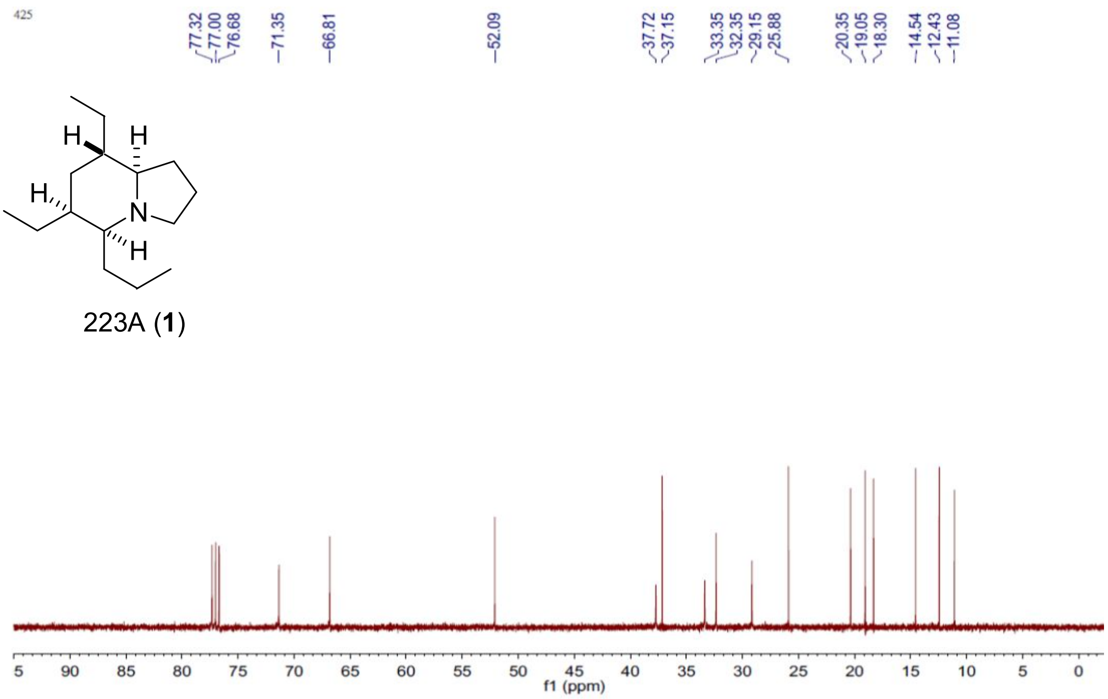

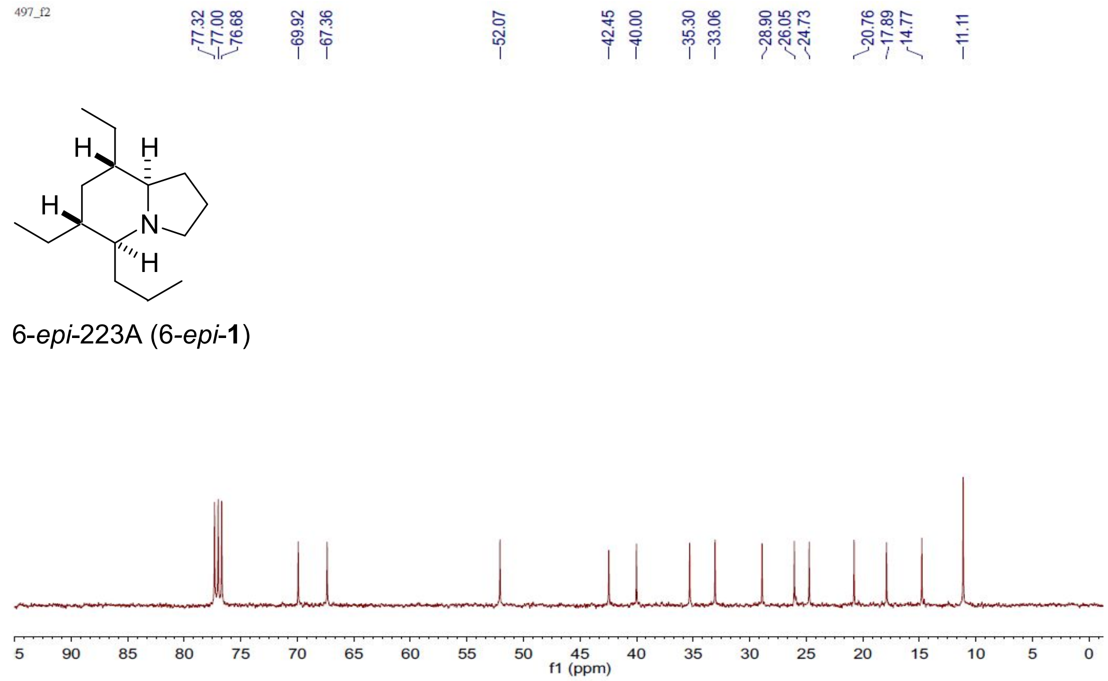

Figure S3. Comparison of the ^{13}C-NMR spectra of our synthetic samples 223A (**1**) and 6-*epi*-223A (6-*epi*-**1**).

Table S1: Comparison of ^{13}C NMR peaks of 223A (**1**) and 6-*epi*-223A (6-*epi*-**1**) with literature values:

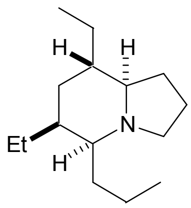

223A, (**1**)

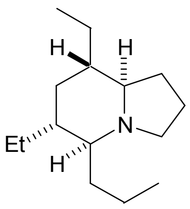

6-*epi*-223A, 6-*epi*-(**1**)

223A, (**1**)		6-*epi*-223A, 6-*epi*-(**1**)	
Synthetic	Lit. value[1]	Synthetic	Lit. value[2]
71.4 (CH)	71.3	69.9 (CH)	70.0
66.8 (CH)	66.8	67.4 (CH)	67.4
52.0 (CH_2)	52.1	52.0 (CH_2)	52.1
37.7 (CH)	37.9	42.4 (CH)	42.4
37.2 (CH)	37.3	40.0 (CH)	40.0
33.4 (CH_2)	33.5	35.3 (CH_2)	35.3
32.4 (CH_2)	32.5	33.0 (CH_2)	33.1
29.2 (CH_2)	29.3	28.9 (CH_2)	28.9
25.9 (CH_2)	26.0	26.0 (CH_2)	26.1
20.4 (CH_2)	20.5	24.7 (CH_2)	24.7
19.1 (CH_2)	19.2	20.8 (CH_2)	20.8
18.3 (CH_2)	18.4	17.9 (CH_2)	17.9
14.5 (CH_3)	14.7	14.8 (CH_3)	14.8
12.4 (CH_3)	12.6	11.1 (CH_3)	11.1
11.0 (CH_3)	11.2	11.1 (CH_3)	11.1

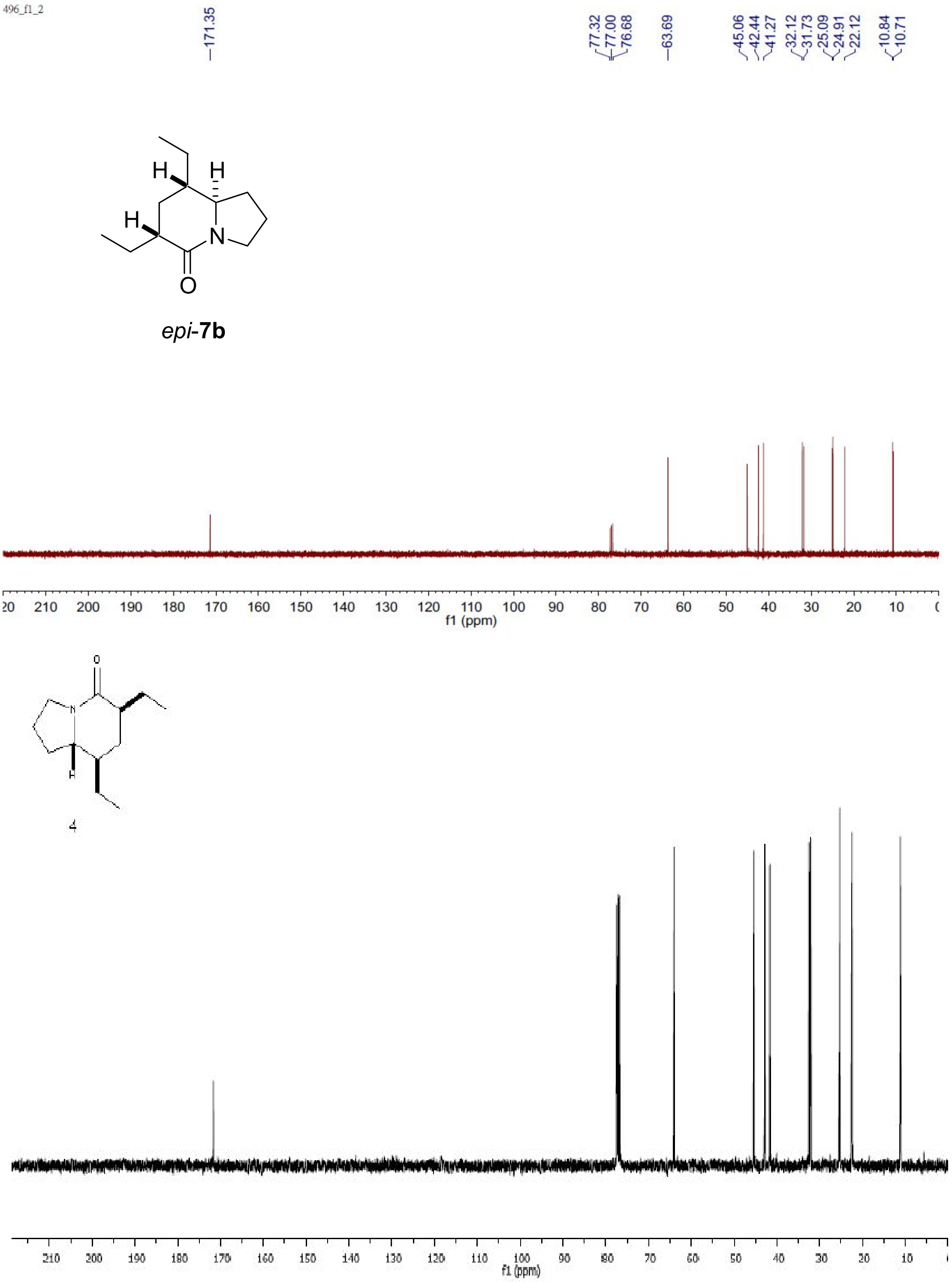

Figure S4. Comparison of the ^{13}C-NMR spectra of *epi*-**7b** with the literature spectrum (lower, Aube et al. *Org. Lett.* **2009**, *11*, 4140-4142. The compound was named as **4** in the Aube's paper).[3]

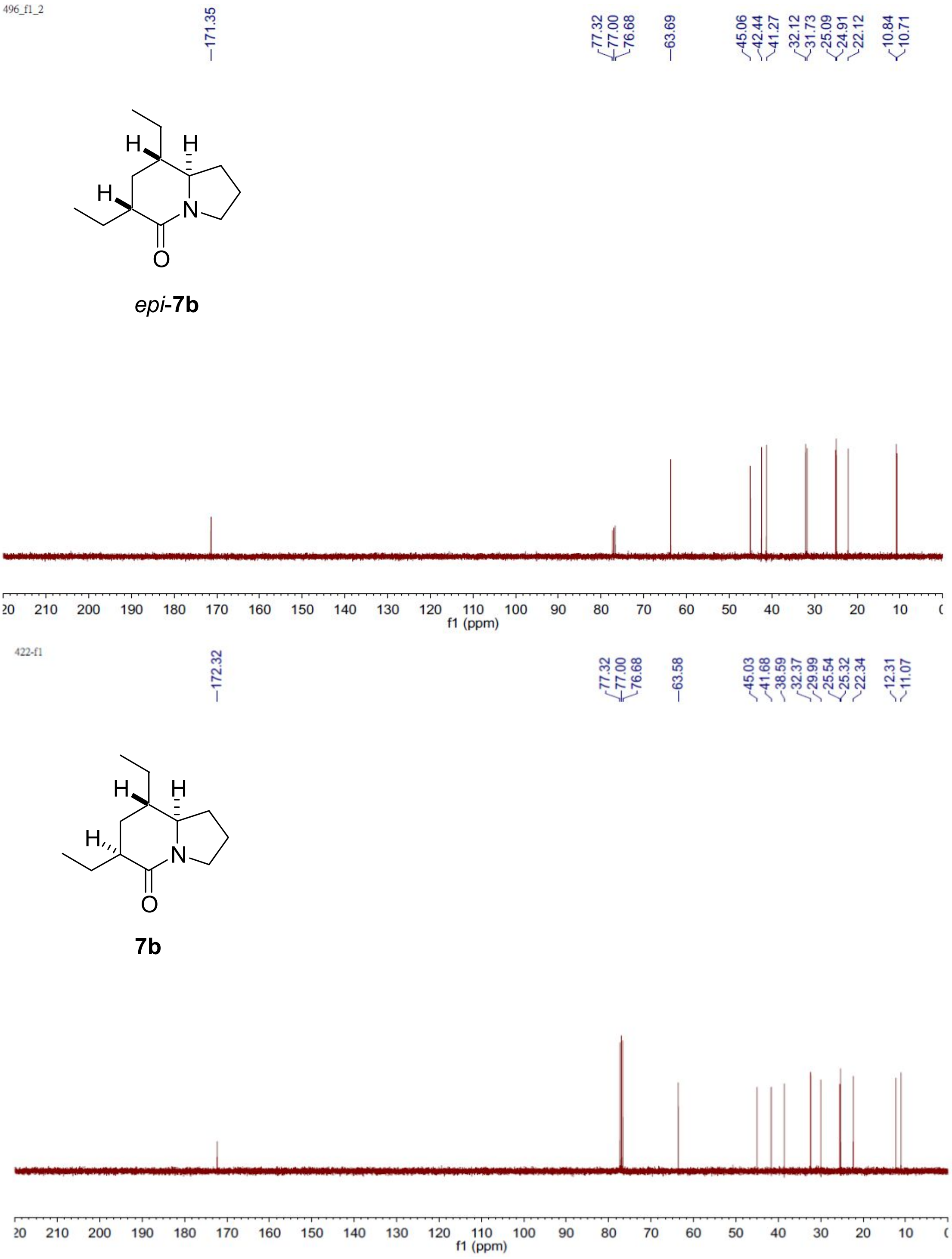

Figure S5. Comparison of the ^{13}C-NMR spectra of our synthetic samples *epi*-**7b** and **7b**.

Table S2: Comparison of ^{13}C NMR peaks of **7b** and *epi*-**7b** with the literature value:

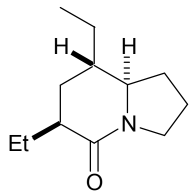

7b

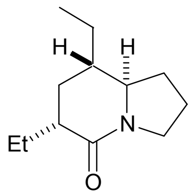

epi-**7b**

**7b**	*epi*-**7b**	
Synthetic	Synthetic	Lit. value[3]
172.3 (C)	171.4 (C)	171.7
63.6 (CH)	63.7 (CH)	64.0
45.0 (CH_2)	45.1 (CH_2)	45.4
41.7 (CH)	42.4 (CH)	42.8
38.6 (CH)	41.3 (CH)	41.6
32.4 (CH_2)	32.1 (CH_2)	32.5
30.0 (CH_2)	31.7 (CH_2)	32.1
25.5 (CH_2)	25.1 (CH_2)	25.4
25.3 (CH_2)	24.9 (CH_2)	25.2
22.3 (CH_2)	22.1 (CH_2)	22.4
12.3 (CH_3)	10.8 (CH_3)	11.2
11.1 (CH_3)	10.7 (CH_3)	11.0

Table S3. Crystal data and structure refinement for **5b** (CCDC no. 1939866).

Identification code	**5b**	
Empirical formula	C19 H27 N3 O3 S	
Formula weight	377.49	
Temperature	150(2) K	
Wavelength	0.71075 Å	
Crystal system	Monoclinic	
Space group	Cc	
Unit cell dimensions	a = 14.719(2) Å	α= 90°.
	b = 12.346(2) Å	β= 103.888(7)°.
	c = 10.840(2) Å	γ = 90°.
Volume	1912.3(5) Å^3	
Z	4	
Density (calculated)	1.311 Mg/m^3	
Absorption coefficient	0.193 mm^{-1}	
F(000)	808	
Crystal size	0.87 x 0.68 x 0.57 mm^3	
Theta range for data collection	4.136 to 26.381°.	
Index ranges	-18<=h<=18, -15<=k<=15, -13<=l<=13	
Reflections collected	13340	
Independent reflections	3569 [R(int) = 0.0366]	
Completeness to theta = 25.243°	98.4 %	
Absorption correction	None	
Refinement method	Full-matrix least-squares on F^2	
Data / restraints / parameters	3569 / 2 / 236	
Goodness-of-fit on F^2	1.012	
Final R indices [I>2sigma(I)]	R1 = 0.0598, wR2 = 0.1826	
R indices (all data)	R1 = 0.0728, wR2 = 0.2089	
Absolute structure parameter	0.04(16)	
Extinction coefficient	n/a	
Largest diff. peak and hole	0.513 and -0.264 e.Å^{-3}	

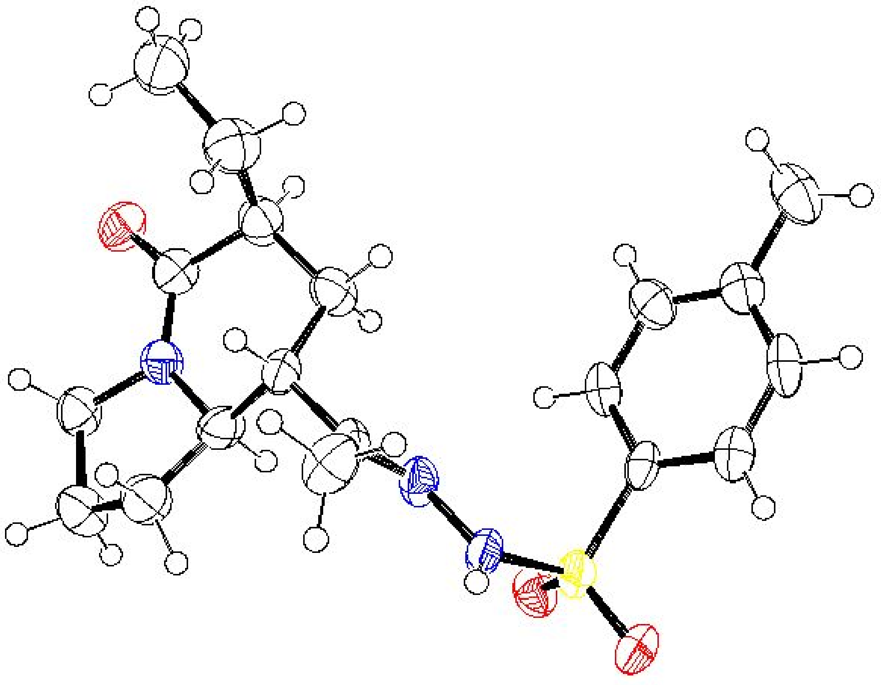

Figure S6. The ORTEP diagram of **5b** with thermal ellipsoids at the 50% contour probability level (CCDC no. 1939866).

Computational Details:

The transition state geometries of the four possible approaches were obtained using *Gausian* 09 [4] at the level of B3LYP[5]/6-31++G**. All geometries have been confirmed as saddle points by vibrational analyses at the same level. Thermal corrections were calculated at 1 atm 298.15 K in the gas phase. The "syn" means the hydride approaches from the same face with H-8a, while the "anti" means the opposite side with H-8a.

1. LAH reduction of the iminium intermediate **7b**.

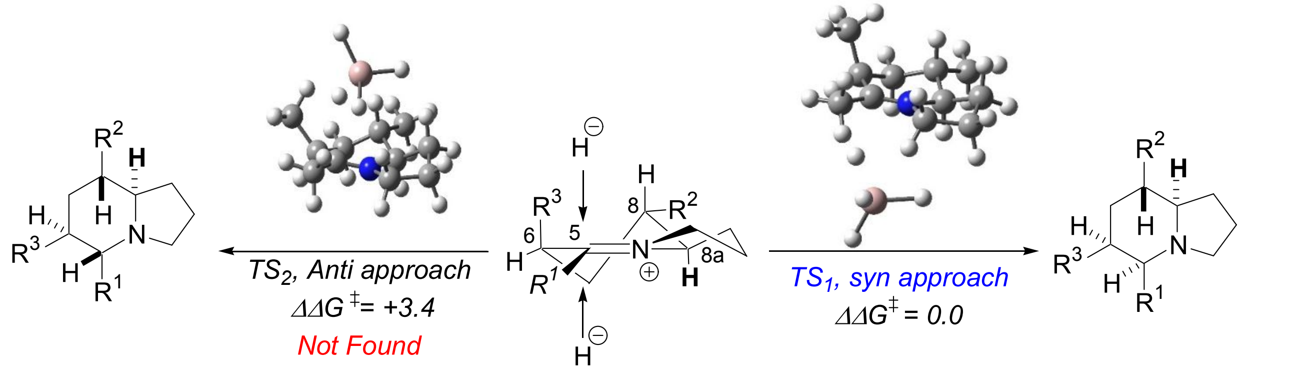

10, $R^1 = R^2 = R^3$ = -CH_3 223A, R^1 = *n*-Pr, $R^2 = R^3$ = Et

2. LAH reduction of the iminium intermediate ***epi*-7b**.

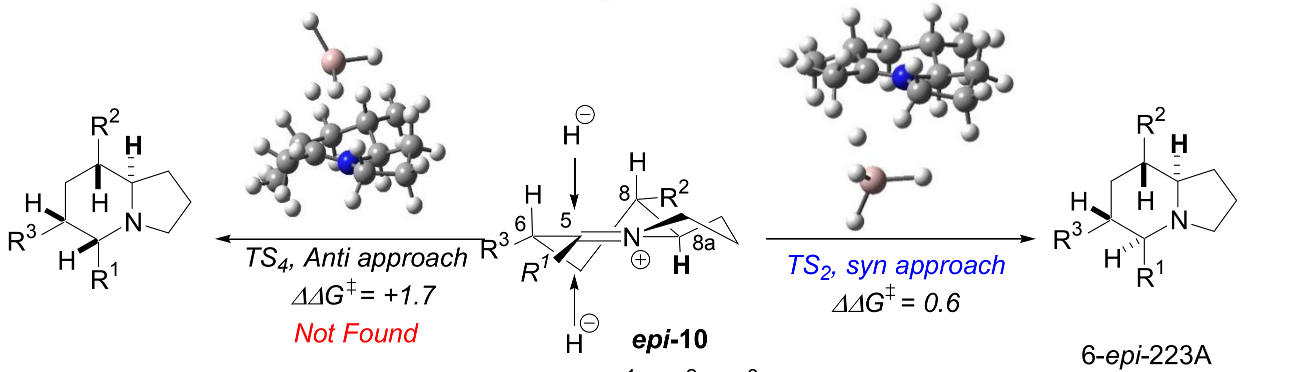

***epi*-10**, $R^1 = R^2 = R^3$ = -CH_3

6-*epi*-223A
R^1 = *n*-Pr, $R^2 = R^3$ = Et

Table S4: Summary of the calculated properties of the 4 possible TSs.

	TS_1, Syn	TS_2, Anti		TS_3, Syn	TS_4, Anti
imaginary freq, (cm^{-1})	-152.23	-123.94		-131.23	-222.39
RB3LYP energies	-730.796560	-730.790890		-730.795713	-730.794056
Zero-point energies	-730.474108	-730.468300		-730.473448	-730.471303
thermal energies	-730.458199	-730.452438		-730.457558	-730.455451
thermal enthalpies	-730.457255	-730.451494		-730.456614	-730.454507
thermal free energies	-730.515900	-730.510416		-730.515020	-730.513262
rel. Energies, (Kcal/mol)	0.0	3.4		0.6	1.7

Computed Cartesian Coordinates of the TS_1 (syn approach to normal 223A)

C	0.435258	1.052953	-0.547156
N	0.292398	-0.237446	-0.663062
H	1.570435	1.164220	1.439204
C	-0.679542	-1.056745	0.125851
H	-0.150154	-1.275890	1.065354
H	3.574111	0.482484	3.110993
H	2.000688	-1.378061	1.881441
H	3.675453	0.062684	0.410018
C	1.521447	1.744223	-1.316798
C	-1.957217	-0.281383	0.435961
C	-0.648498	1.888021	0.104878
H	-0.125491	2.650805	0.688645
C	-1.546872	1.070717	1.043703
H	-2.436676	1.659076	1.295378
C	1.195938	-1.119646	-1.453966
H	2.227213	-0.963865	-1.129077
H	1.100527	-0.844622	-2.510356
C	-0.769000	-2.338868	-0.704323
H	-1.447436	-2.195461	-1.556050
H	-1.137446	-3.179892	-0.112786
H	1.559899	2.800519	-1.049462
H	2.491315	1.293304	-1.086570
H	1.337566	1.659497	-2.396068
H	-1.000994	0.887735	1.976617
H	-2.496726	-0.111037	-0.507449
C	0.683005	-2.541035	-1.173271
H	1.273880	-2.987814	-0.368953
H	0.763418	-3.180165	-2.055971
Al	2.774729	0.060851	1.780943
C	-2.871639	-1.072703	1.380422
H	-3.769264	-0.492102	1.617175
H	-2.360602	-1.298094	2.323441
H	-3.195599	-2.018868	0.935417
C	-1.457161	2.610367	-1.004898
H	-2.175737	3.289366	-0.536088
H	-2.015697	1.905088	-1.628683
H	-0.815247	3.206737	-1.659419

Computed Cartesian Coordinates of the TS_2 (Anti Approach, Not Found):

C	2.010895	1.254639	-0.152210
C	1.772073	-0.030892	0.652313
C	1.095214	-1.027726	-0.283634
C	-0.379934	0.855699	-0.965902
N	-0.110243	-0.420475	-0.932996
C	-1.109197	-1.478532	-1.263667
H	-1.321765	-1.461532	-2.336878
H	-2.027219	-1.254114	-0.710631
C	-1.640825	1.308866	-1.645528
H	-2.519140	0.818768	-1.218890
H	-1.580173	1.060286	-2.714058
H	-1.766100	2.386153	-1.549819
H	-3.591771	-0.369961	0.574294
H	-1.842822	-1.263083	2.385428
H	-3.375746	0.929789	2.961455
H	-1.444758	0.972814	1.098583
H	2.549522	1.988597	0.455974
H	1.075381	0.181328	1.473186
H	1.791958	-1.277711	-1.100120
H	2.662054	1.019687	-1.005582
C	0.689220	1.885246	-0.643410
H	0.868516	2.360319	-1.622249
Al	-2.627672	0.057395	1.832907
C	0.529005	-2.326014	0.300331
H	1.315555	-3.059855	0.492859
H	-0.003204	-2.112856	1.234102
C	-0.461802	-2.785158	-0.782109
H	-1.215603	-3.474302	-0.395290
H	0.066612	-3.284097	-1.602655
C	0.213242	3.005992	0.305708
H	0.953065	3.812858	0.290271
H	0.107668	2.627443	1.323739
H	-0.752999	3.423832	0.016751
C	3.073774	-0.598075	1.229875
H	3.556581	0.141743	1.876364
H	3.783123	-0.859315	0.434283
H	2.892701	-1.493875	1.831470

Computed Cartesian Coordinates of the TS_3 (syn approach to 6-*epi*-223A):

C	0.824836	0.417798	-0.897027
N	-0.060948	-0.516044	-0.685467
H	1.813883	0.263922	1.281428
C	-1.258414	-0.361470	0.193442
H	-0.881712	-0.545926	1.210714
H	3.177765	-1.104279	3.168650
H	0.802383	-1.979924	2.148365
H	2.949620	-1.950245	0.581315
C	2.032427	0.077703	-1.717965
C	-1.828791	1.051561	0.106480
C	0.495970	1.869738	-0.592588
H	0.177977	2.237365	-1.586734
C	-0.683609	2.036407	0.376196
H	-1.048320	3.067896	0.309022
C	0.075888	-1.927438	-1.144824
H	1.043018	-2.320418	-0.824634
H	0.022292	-1.935651	-2.240033
C	-2.139109	-1.537345	-0.235837
H	-2.698855	-1.281586	-1.145657
H	-2.857608	-1.807333	0.541157
H	2.623869	0.962657	-1.942947
H	2.654870	-0.631686	-1.157016
H	1.736391	-0.398459	-2.659905
H	-0.314399	1.889865	1.399094
H	-2.204132	1.207887	-0.917013
C	-1.115203	-2.653638	-0.504249
H	-0.797945	-3.102777	0.440704
H	-1.501961	-3.442858	-1.153733
Al	2.232842	-1.234211	1.873615
C	1.691327	2.734780	-0.156321
H	2.489923	2.763551	-0.901639
H	2.104983	2.357964	0.781672
H	1.345200	3.762845	-0.009785
C	-2.991136	1.247363	1.088448
H	-3.363553	2.275865	1.039485
H	-2.670052	1.056254	2.118903
H	-3.829056	0.579369	0.865328

Computed Cartesian Coordinates of the TS_4 (Anti Approach, Not Found):

C	2.093536	-0.389404	0.709426
C	0.882724	-1.321085	0.847421
C	0.147549	-1.329708	-0.493121
C	0.375119	1.139473	-0.441355
N	-0.215546	0.066960	-0.902207
C	-1.521631	0.087361	-1.620285
H	-1.410400	0.602976	-2.578242
H	-2.238257	0.632239	-0.995830
C	-0.091494	2.492712	-0.899914
H	-1.176801	2.581467	-0.849414
H	0.232792	2.656793	-1.937373
H	0.343534	3.269578	-0.271131
H	-3.101574	1.728723	0.742632
H	-2.260881	-0.348345	2.205762
H	-2.355489	2.150651	3.331407
H	-0.607204	1.477582	1.415644
H	2.627147	-0.321309	1.663581
H	0.192396	-0.915240	1.596119
H	0.815486	-1.727783	-1.273666
H	2.795963	-0.840355	-0.006634
C	1.729458	1.037475	0.237057
Al	-2.162930	1.266594	2.000457
C	-1.201232	-2.056115	-0.561360
H	-1.073728	-3.133653	-0.690374
H	-1.760005	-1.871293	0.362785
C	-1.901400	-1.392141	-1.757294
H	-2.985670	-1.524219	-1.740117
H	-1.525732	-1.803988	-2.701368
C	1.301634	-2.735631	1.264394
H	1.853715	-2.705000	2.209276
H	1.952760	-3.199393	0.512063
H	0.433853	-3.385427	1.412068
C	2.829647	1.604450	-0.693207
H	3.794303	1.543257	-0.179920
H	2.657927	2.652271	-0.953996
H	2.906900	1.023762	-1.619368
H	1.652422	1.697943	1.105034

Alkaloid 223A (1), ^{1}H-NMR (400MHz, $CDCl_3$)

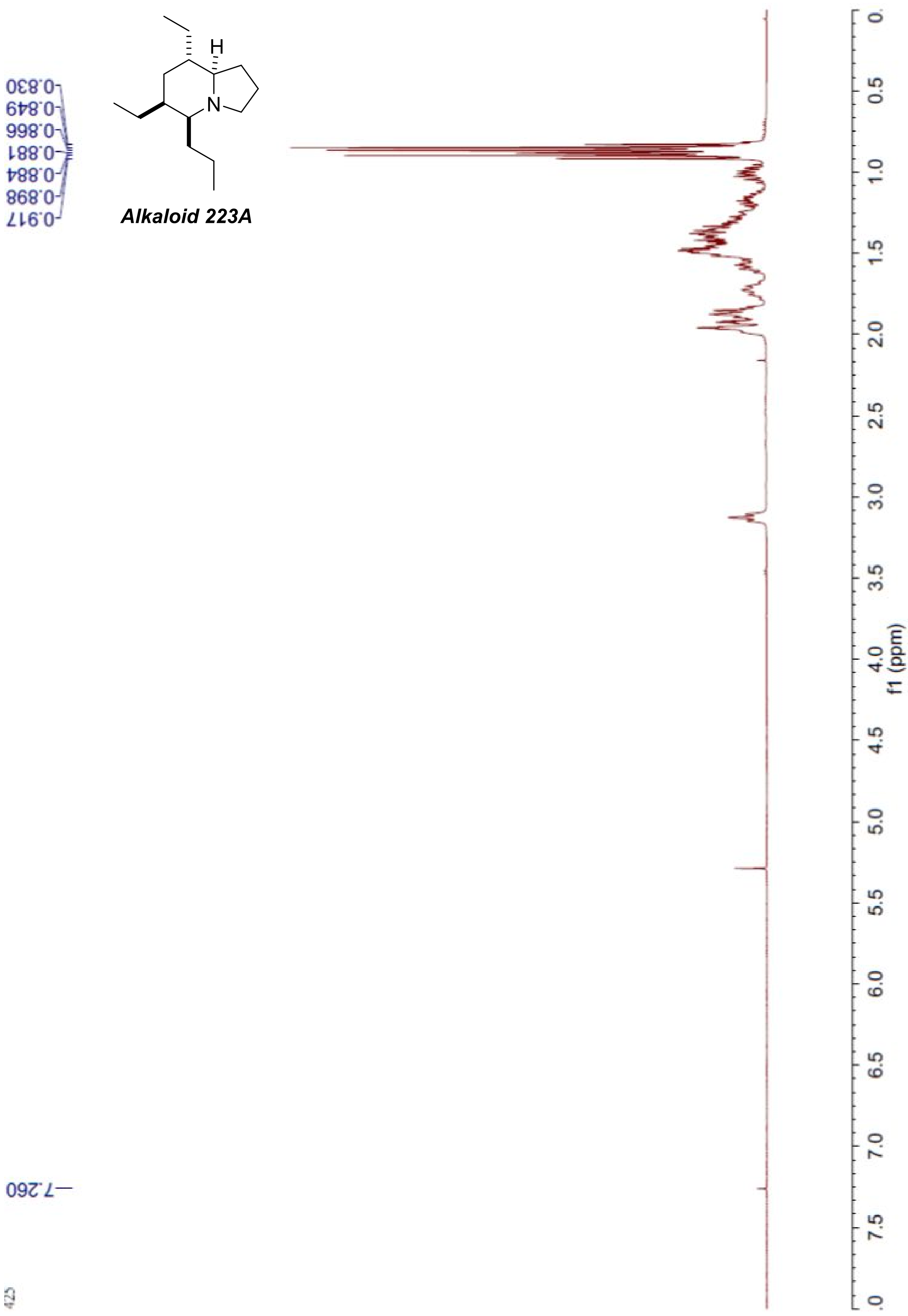

Alkaloid 223A (1), $^{13}C\{^{1}H\}$ NMR (101 MHz, $CDCl_3$)

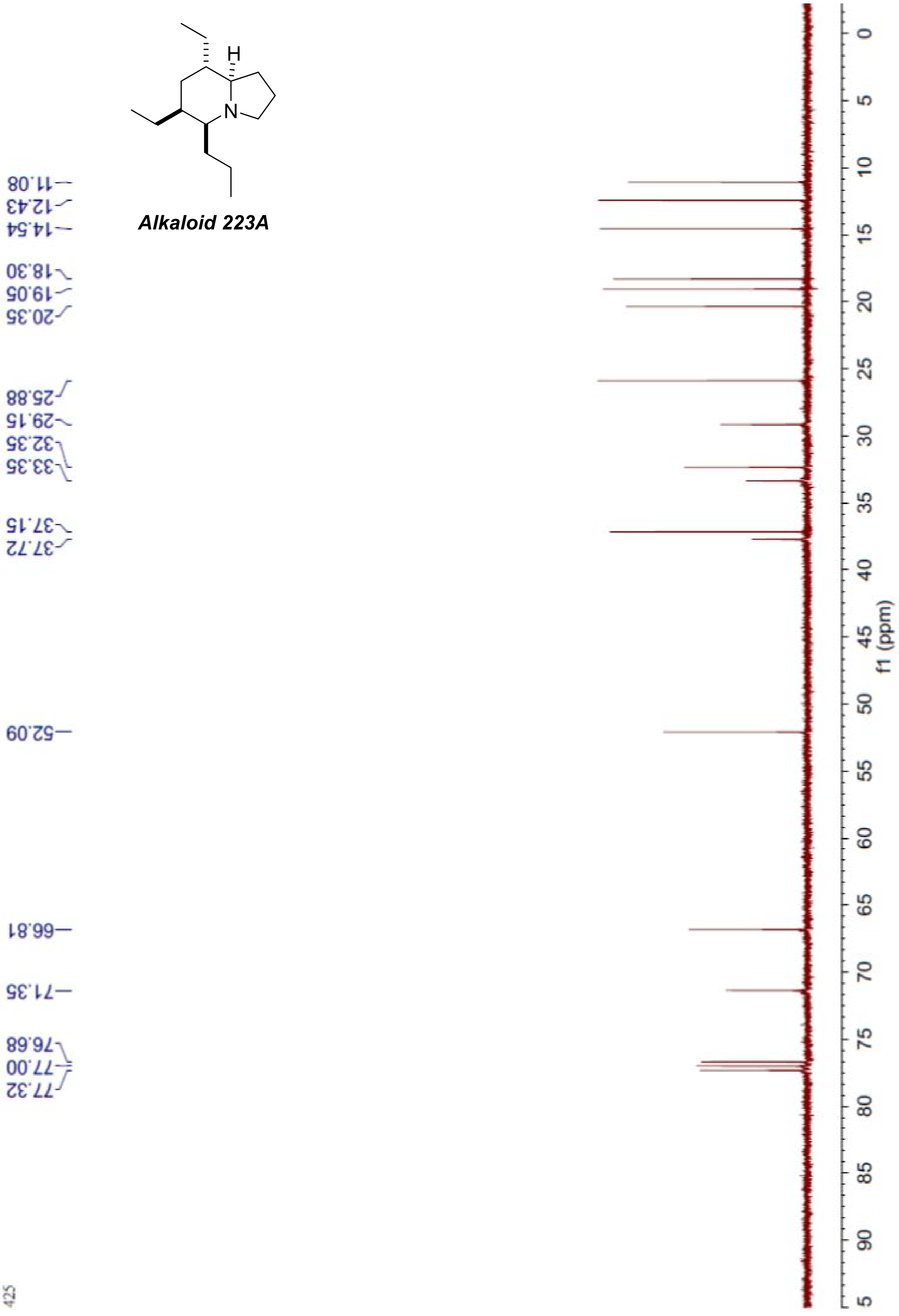

Alkaloid 223A (1), DEPT (101 MHz, $CDCl_3$)

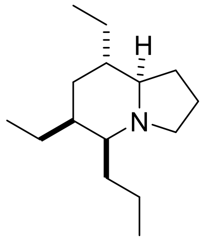

Alkaloid 223A

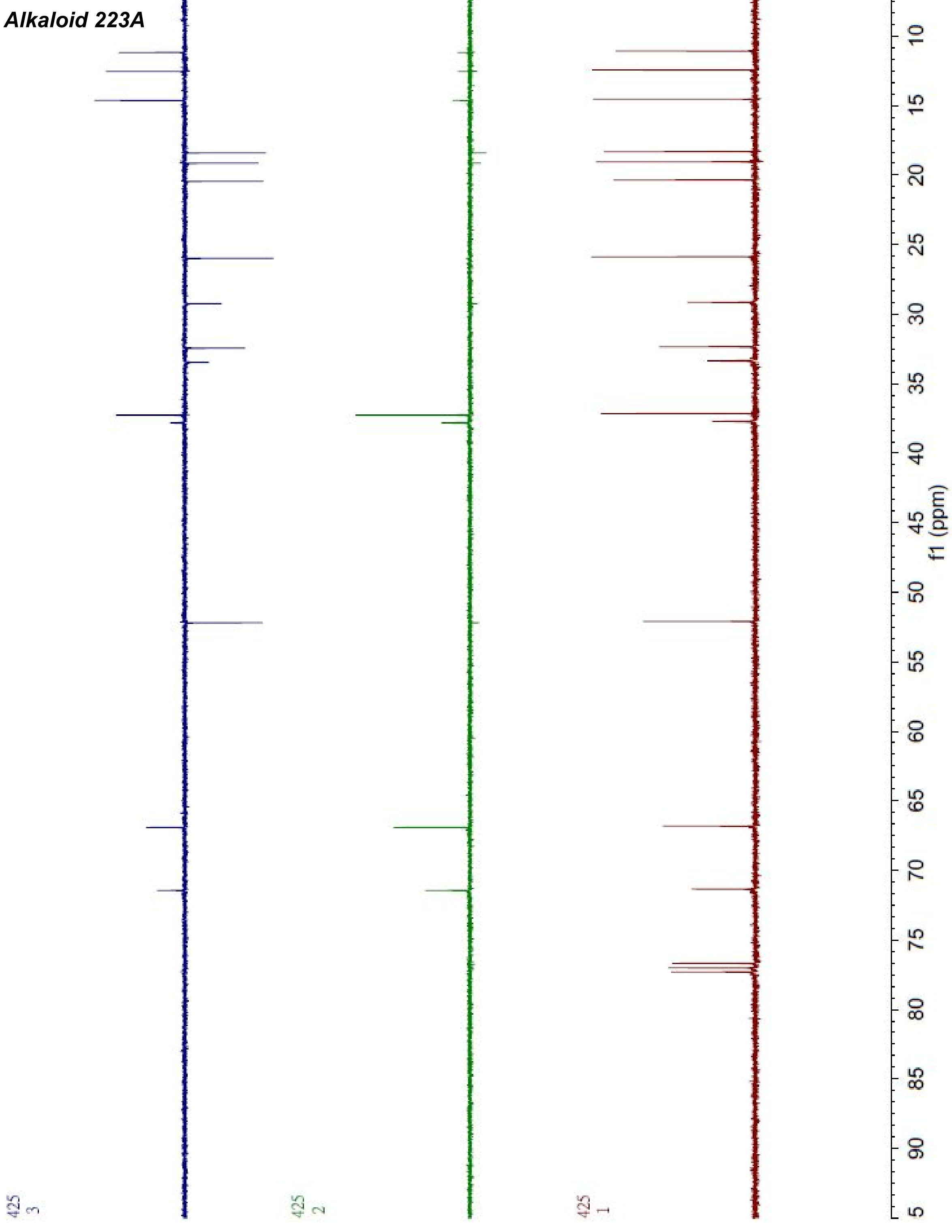

Alkaloid 6-*epi*-223A, 6-*epi*-(1), ^{1}H-NMR (400MHz, $CDCl_3$)

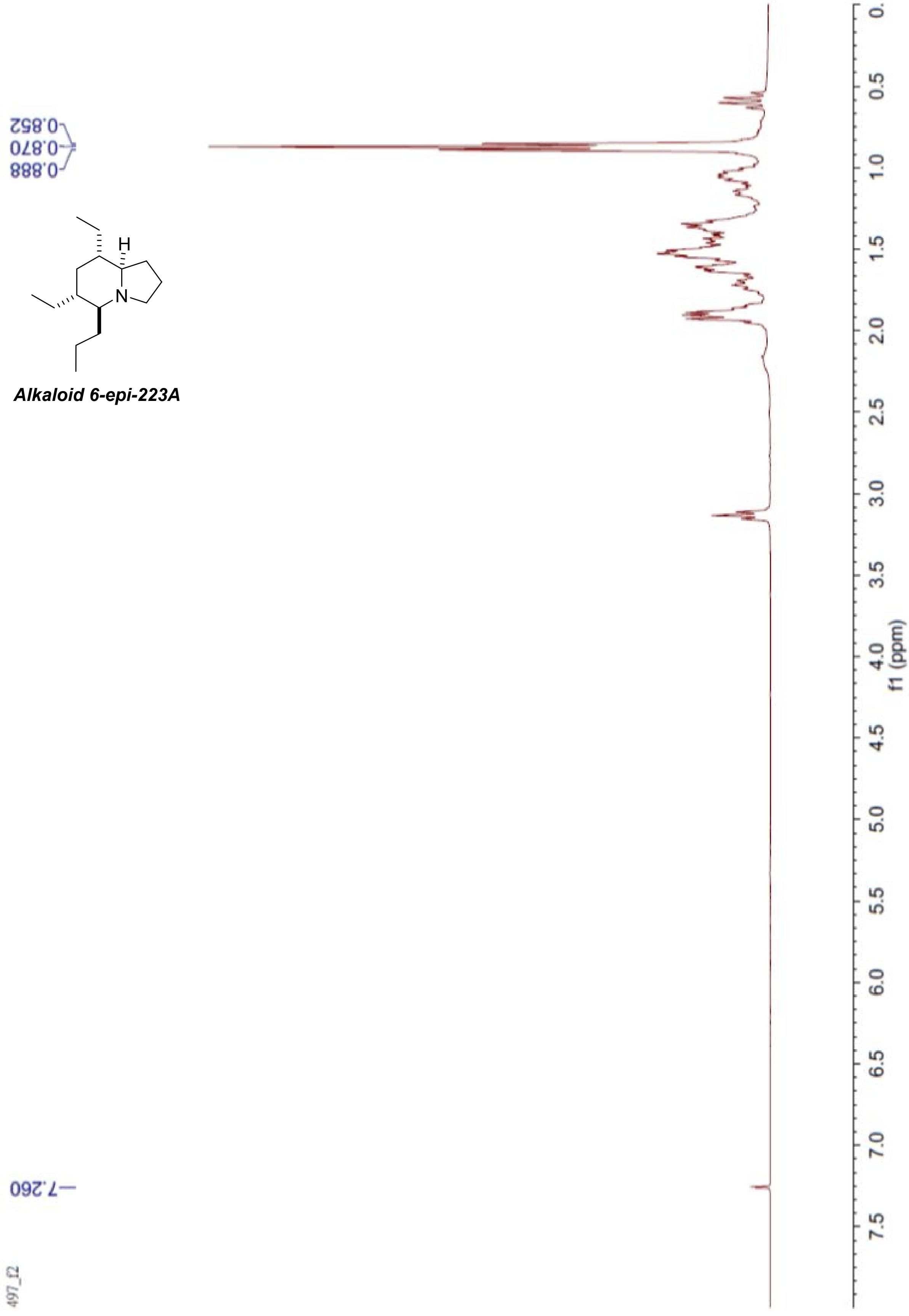

Alkaloid 6-*epi*-223A, 6-*epi*-(1), $^{13}C\{^{1}H\}$ NMR (101 MHz, $CDCl_3$)

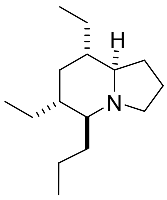

Alkaloid 6-epi-223A

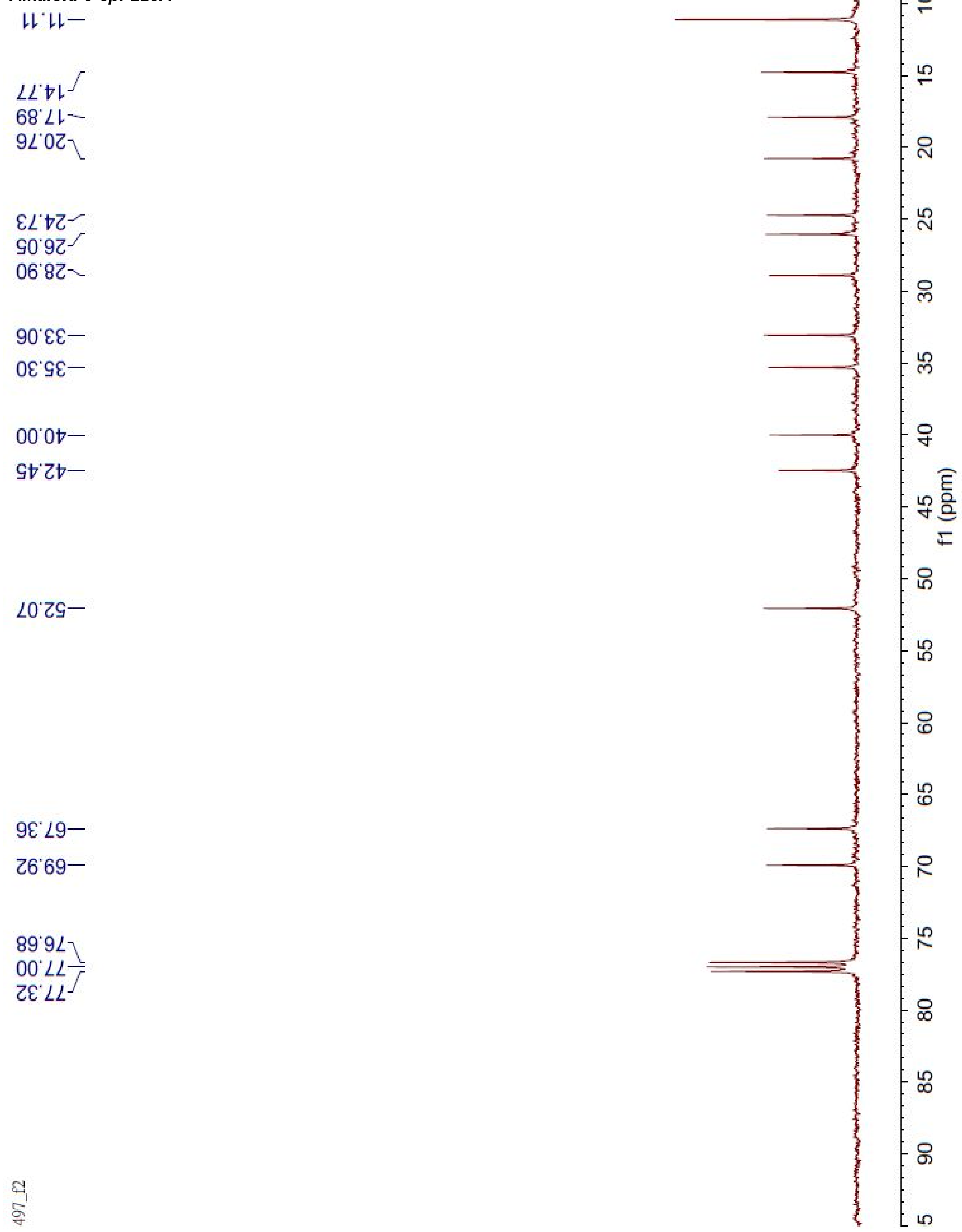

Alkaloid 6-*epi*-223A, 6-*epi*-(1) DEPT, (101 MHz, $CDCl_3$)

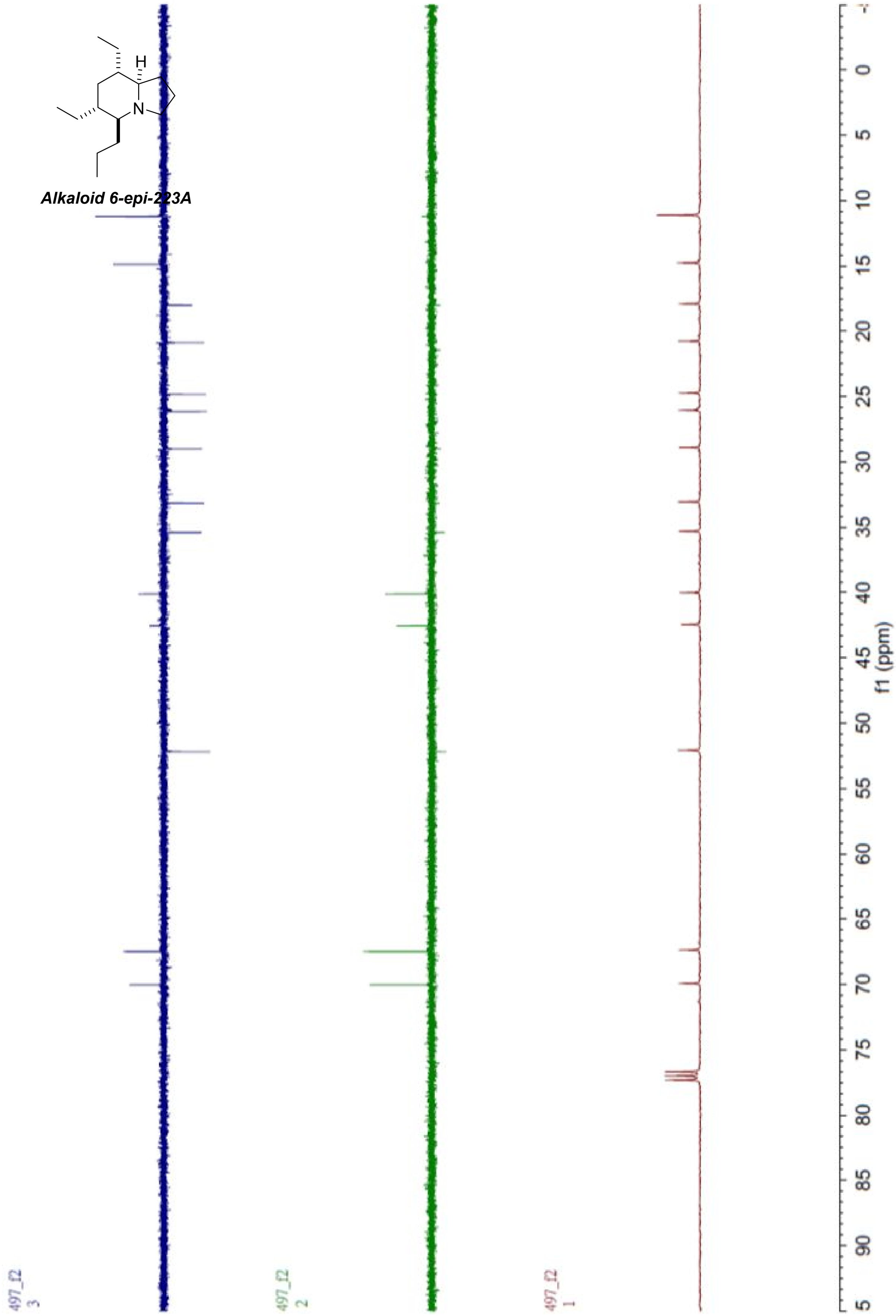

2a, ^{1}H-NMR (400MHz, $CDCl_3$)

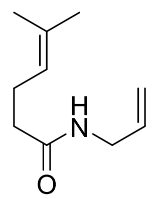

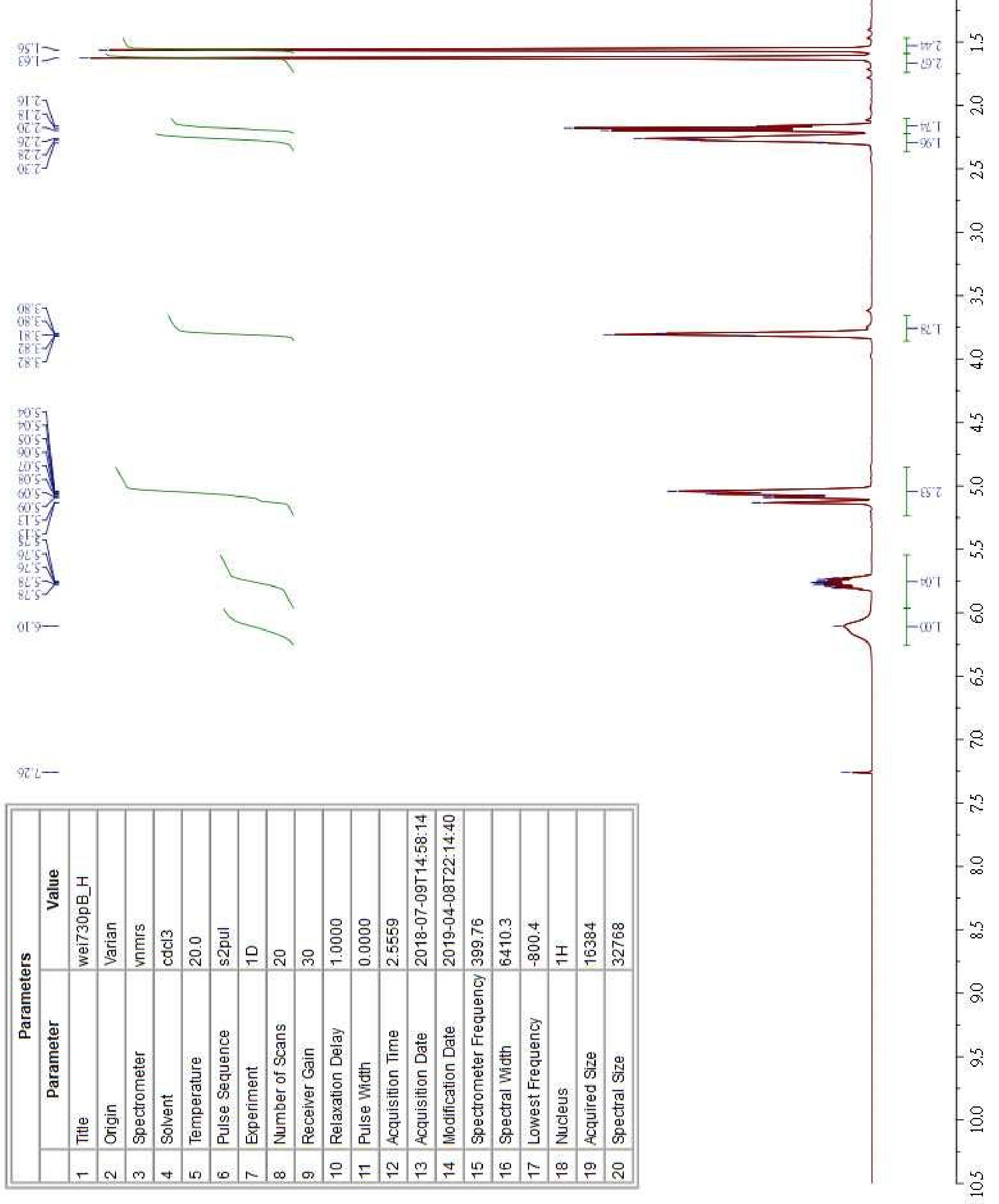

	Parameter	Value
	**Parameters**	
1	Title	wei730pB_H
2	Origin	Varian
3	Spectrometer	vnmrs
4	Solvent	cdcl3
5	Temperature	20.0
6	Pulse Sequence	s2pul
7	Experiment	1D
8	Number of Scans	20
9	Receiver Gain	30
10	Relaxation Delay	1.0000
11	Pulse Width	0.0000
12	Acquisition Time	2.5559
13	Acquisition Date	2018-07-09T14:58:14
14	Modification Date	2019-04-08T22:14:40
15	Spectrometer Frequency	399.76
16	Spectral Width	6410.3
17	Lowest Frequency	-800.4
18	Nucleus	1H
19	Acquired Size	16384
20	Spectral Size	32768

2a, $^{13}C\{^{1}H\}$ NMR (101 MHz, $CDCl_3$)

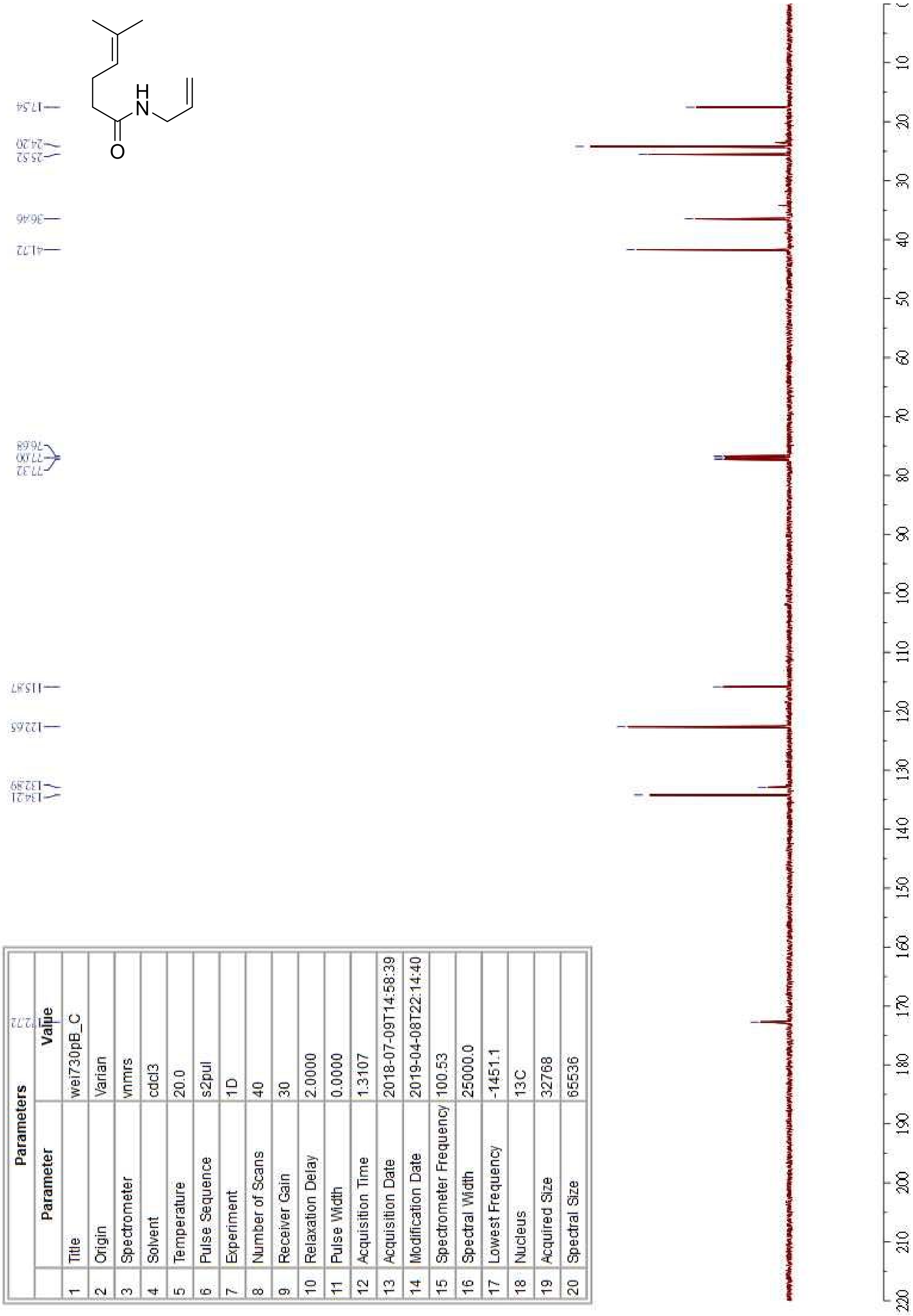

	Parameter	Value
	**Parameters**	
1	Title	wei730pB_C
2	Origin	Varian
3	Spectrometer	vnmrs
4	Solvent	cdcl3
5	Temperature	20.0
6	Pulse Sequence	s2pul
7	Experiment	1D
8	Number of Scans	40
9	Receiver Gain	30
10	Relaxation Delay	2.0000
11	Pulse Width	0.0000
12	Acquisition Time	1.3107
13	Acquisition Date	2018-07-09T14:58:39
14	Modification Date	2019-04-08T22:14:40
15	Spectrometer Frequency	100.53
16	Spectral Width	25000.0
17	Lowest Frequency	-1451.1
18	Nucleus	13C
19	Acquired Size	32768
20	Spectral Size	65536

2b, ^{1}H-NMR (400MHz, $CDCl_3$)

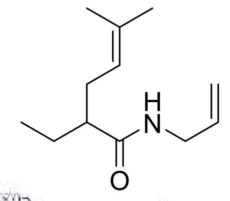

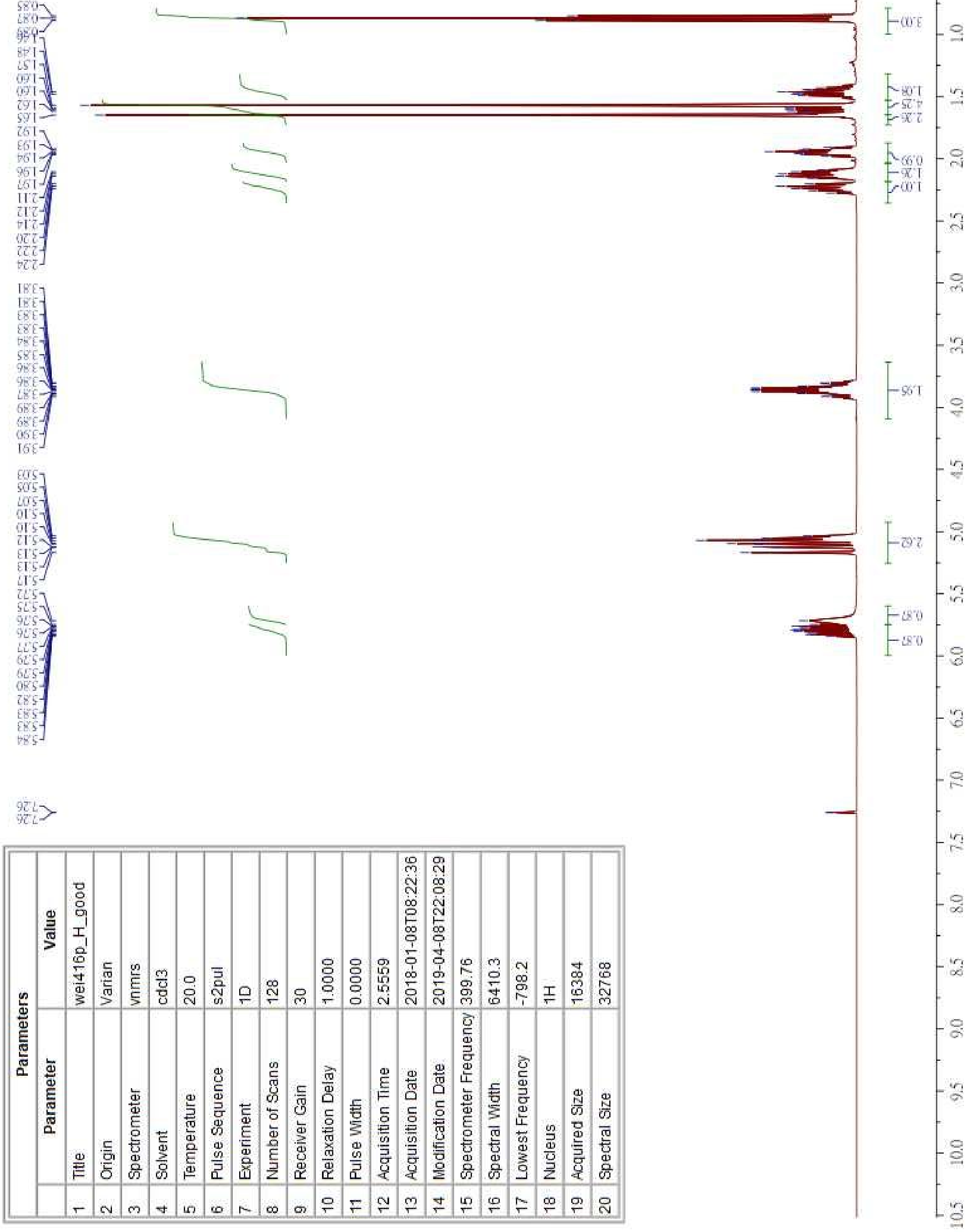

	Parameter	Value
1	Title	wei416p_H_good
2	Origin	Varian
3	Spectrometer	vnmrs
4	Solvent	cdcl3
5	Temperature	20.0
6	Pulse Sequence	s2pul
7	Experiment	1D
8	Number of Scans	128
9	Receiver Gain	30
10	Relaxation Delay	1.0000
11	Pulse Width	0.0000
12	Acquisition Time	2.5559
13	Acquisition Date	2018-01-08T08:22:36
14	Modification Date	2019-04-08T22:08:29
15	Spectrometer Frequency	399.76
16	Spectral Width	6410.3
17	Lowest Frequency	-798.2
18	Nucleus	1H
19	Acquired Size	16384
20	Spectral Size	32768

2b, $^{13}C\{^{1}H\}$ NMR (101 MHz, $CDCl_3$)

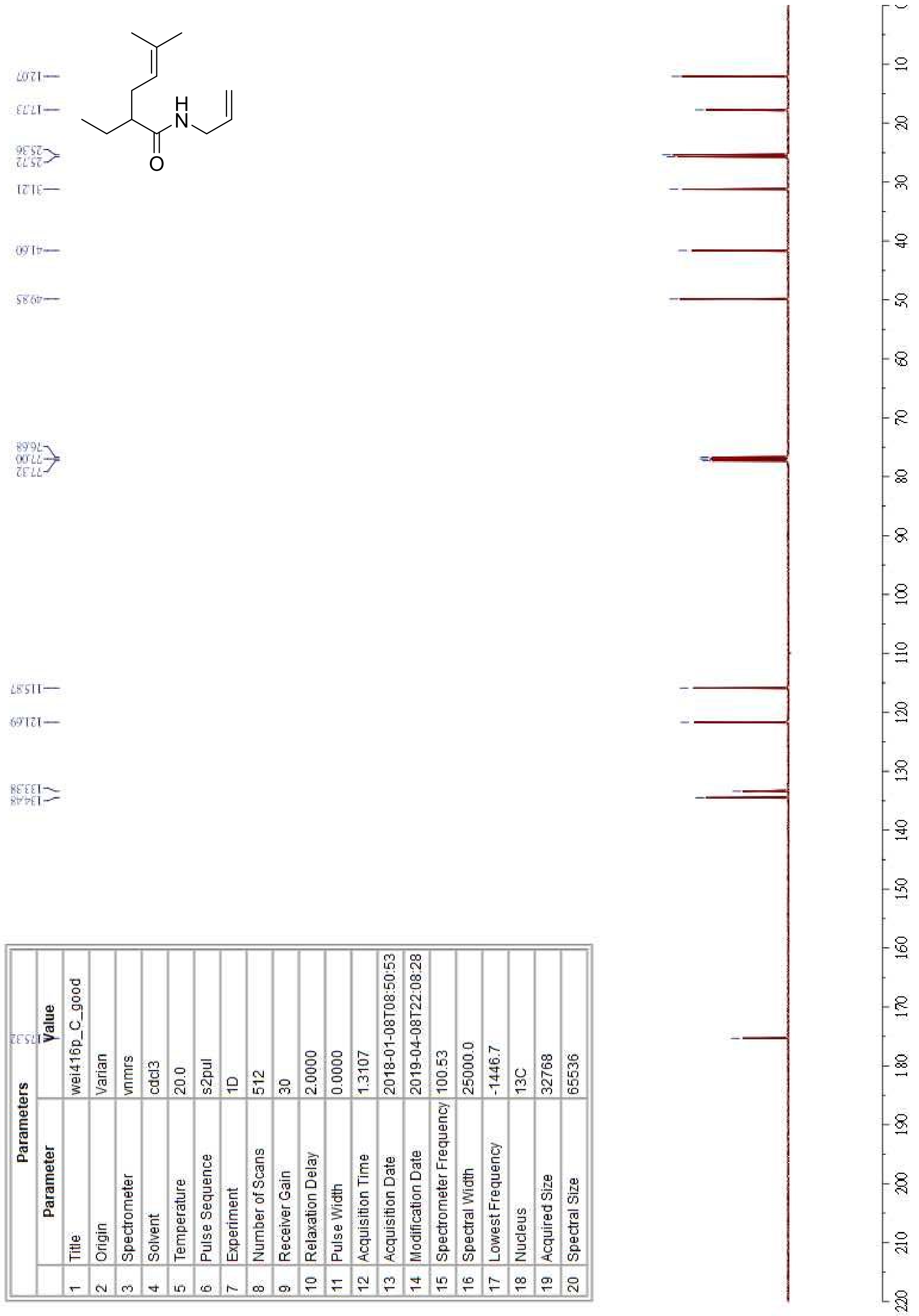

Parameters		
	Parameter	Value
1	Title	wei416p_C_good
2	Origin	Varian
3	Spectrometer	vnmrs
4	Solvent	cdcl3
5	Temperature	20.0
6	Pulse Sequence	s2pul
7	Experiment	1D
8	Number of Scans	512
9	Receiver Gain	30
10	Relaxation Delay	2.0000
11	Pulse Width	0.0000
12	Acquisition Time	1.3107
13	Acquisition Date	2018-01-08T08:50:53
14	Modification Date	2019-04-08T22:08:28
15	Spectrometer Frequency	100.53
16	Spectral Width	25000.0
17	Lowest Frequency	-1446.7
18	Nucleus	13C
19	Acquired Size	32768
20	Spectral Size	65536

2b, DEPT (101 MHz, $CDCl_3$)

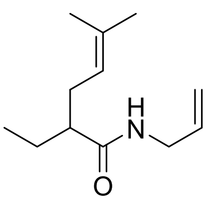

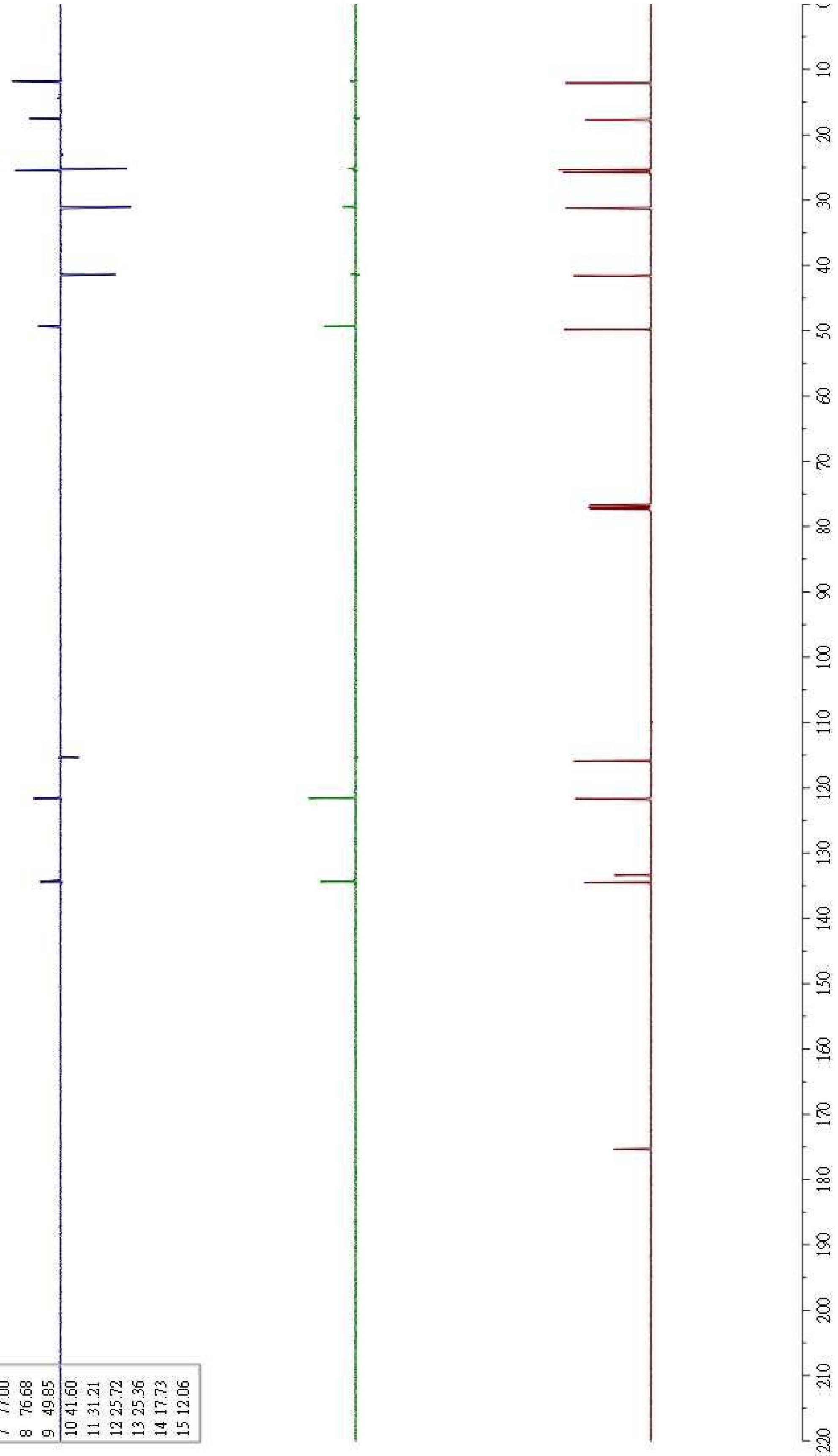

3a, ^{1}H-NMR (400MHz, $CDCl_3$)

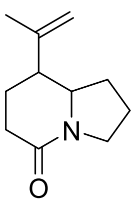

trans/cis

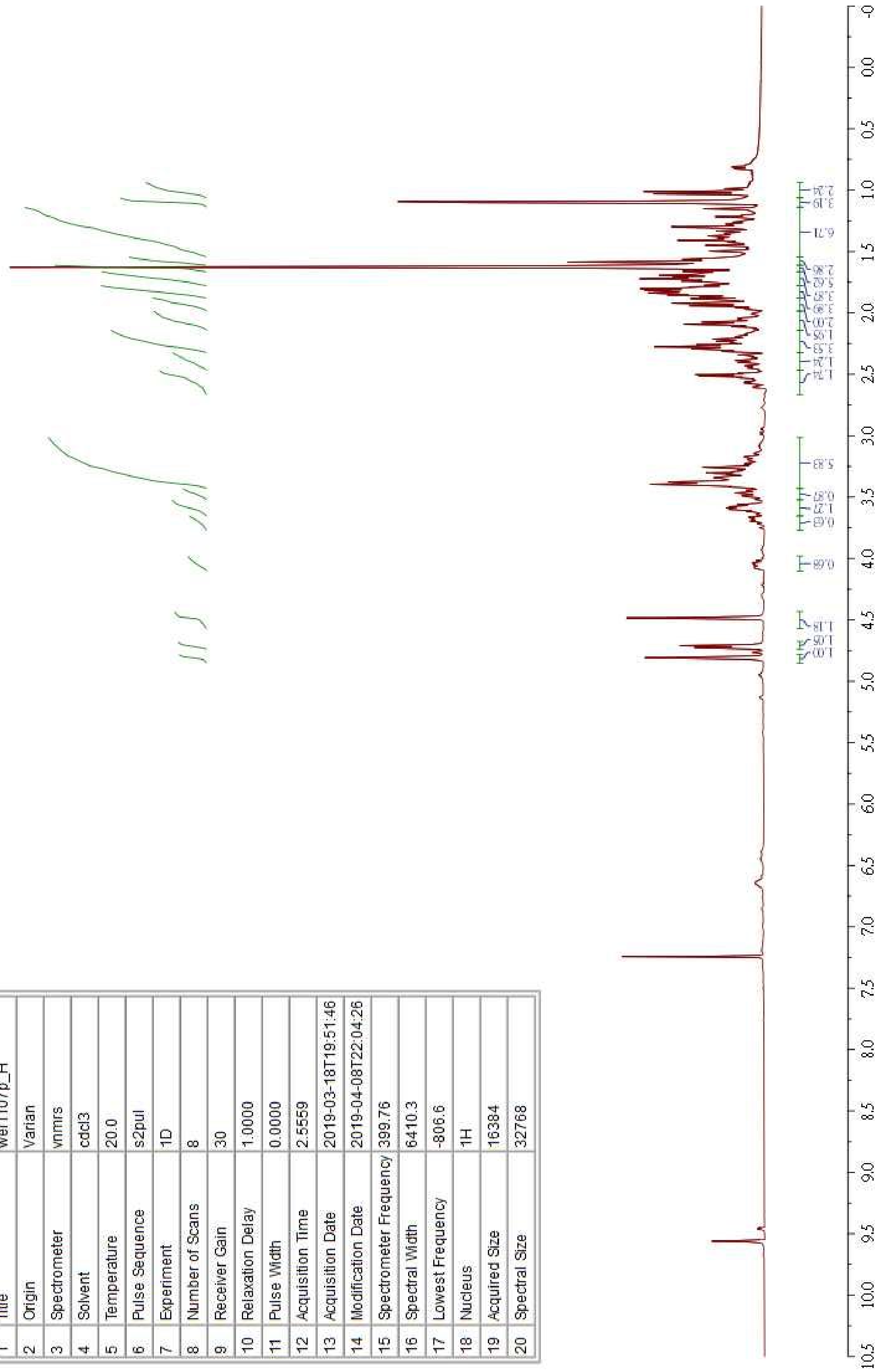

	Parameters	
	Parameter	Value
1	Title	wei1107p_H
2	Origin	Varian
3	Spectrometer	vnmrs
4	Solvent	cdcl3
5	Temperature	20.0
6	Pulse Sequence	s2pul
7	Experiment	1D
8	Number of Scans	8
9	Receiver Gain	30
10	Relaxation Delay	1.0000
11	Pulse Width	0.0000
12	Acquisition Time	2.5559
13	Acquisition Date	2019-03-18T19:51:46
14	Modification Date	2019-04-08T22:04:26
15	Spectrometer Frequency	399.76
16	Spectral Width	6410.3
17	Lowest Frequency	-806.6
18	Nucleus	1H
19	Acquired Size	16384
20	Spectral Size	32768

3a, $^{13}C\{^1H\}$ NMR (101 MHz, $CDCl_3$)

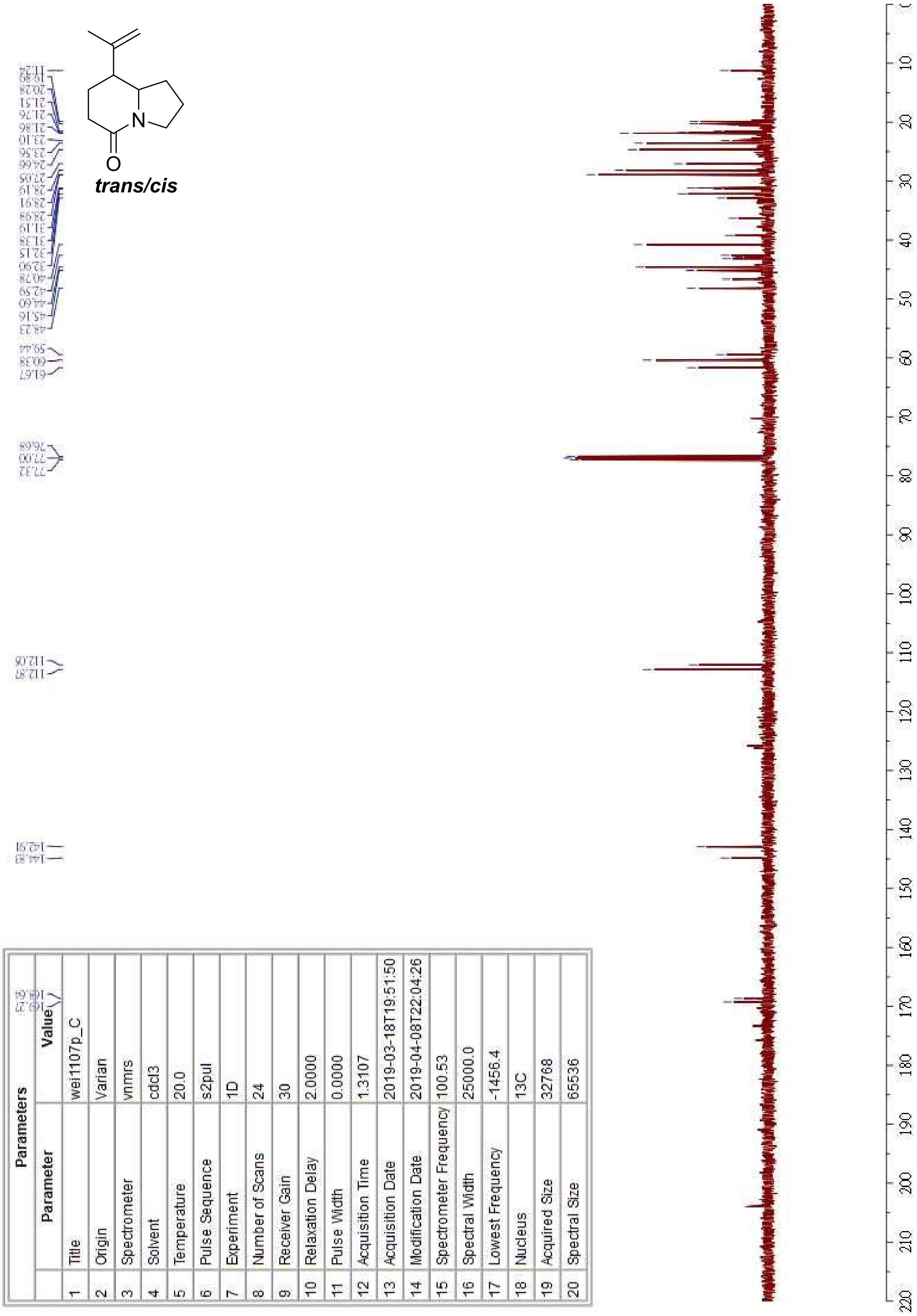

Parameters

	Parameter	Value
1	Title	wei1107p_C
2	Origin	Varian
3	Spectrometer	vnmrs
4	Solvent	cdcl3
5	Temperature	20.0
6	Pulse Sequence	s2pul
7	Experiment	1D
8	Number of Scans	24
9	Receiver Gain	30
10	Relaxation Delay	2.0000
11	Pulse Width	0.0000
12	Acquisition Time	1.3107
13	Acquisition Date	2019-03-18T19:51:50
14	Modification Date	2019-04-08T22:04:26
15	Spectrometer Frequency	100.53
16	Spectral Width	25000.0
17	Lowest Frequency	-1456.4
18	Nucleus	13C
19	Acquired Size	32768
20	Spectral Size	65536

3b$_1$, ^{1}H-NMR (400MHz, $CDCl_3$)

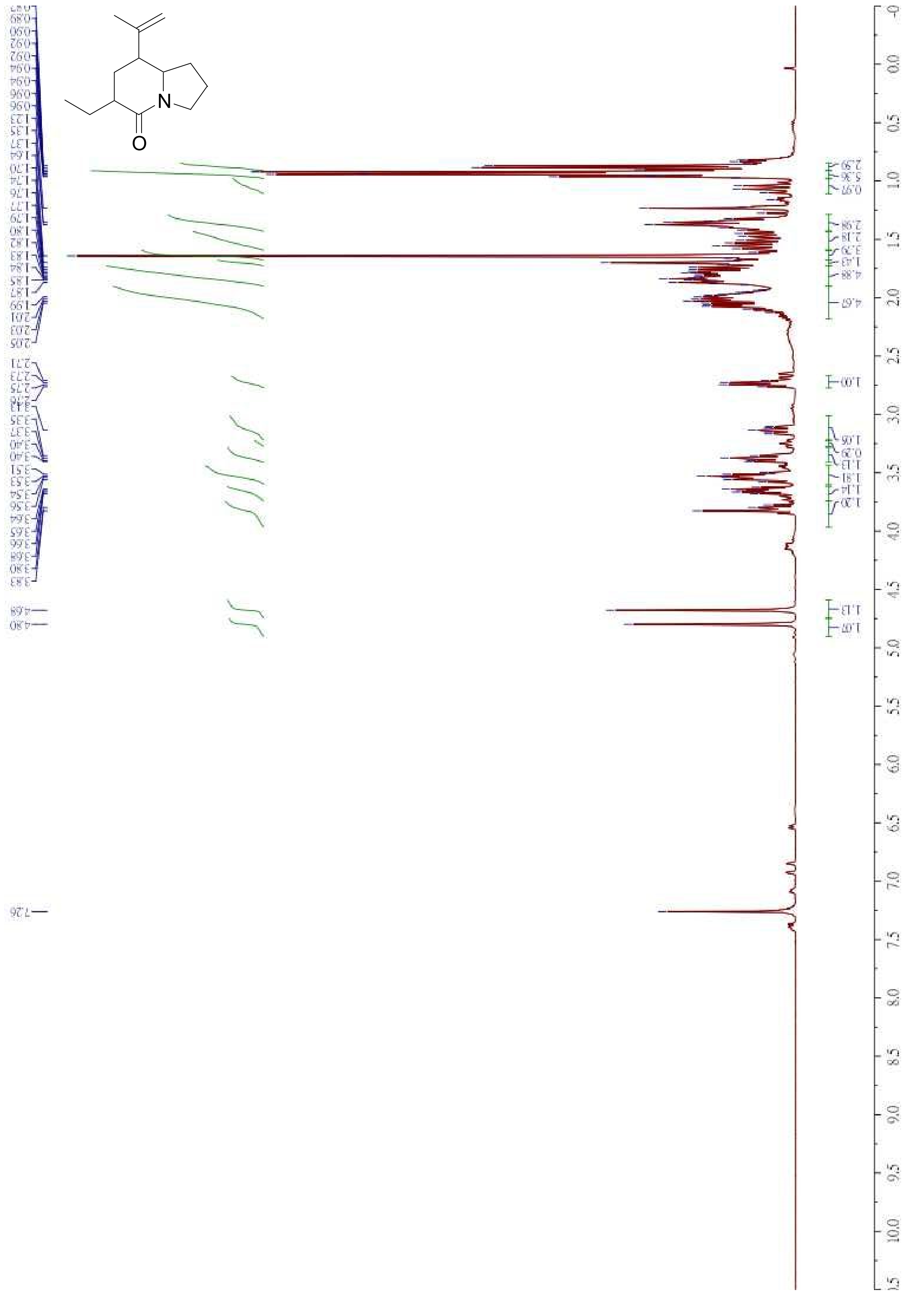

3b₁, $^{13}C\{^{1}H\}$ NMR (101 MHz, $CDCl_3$)

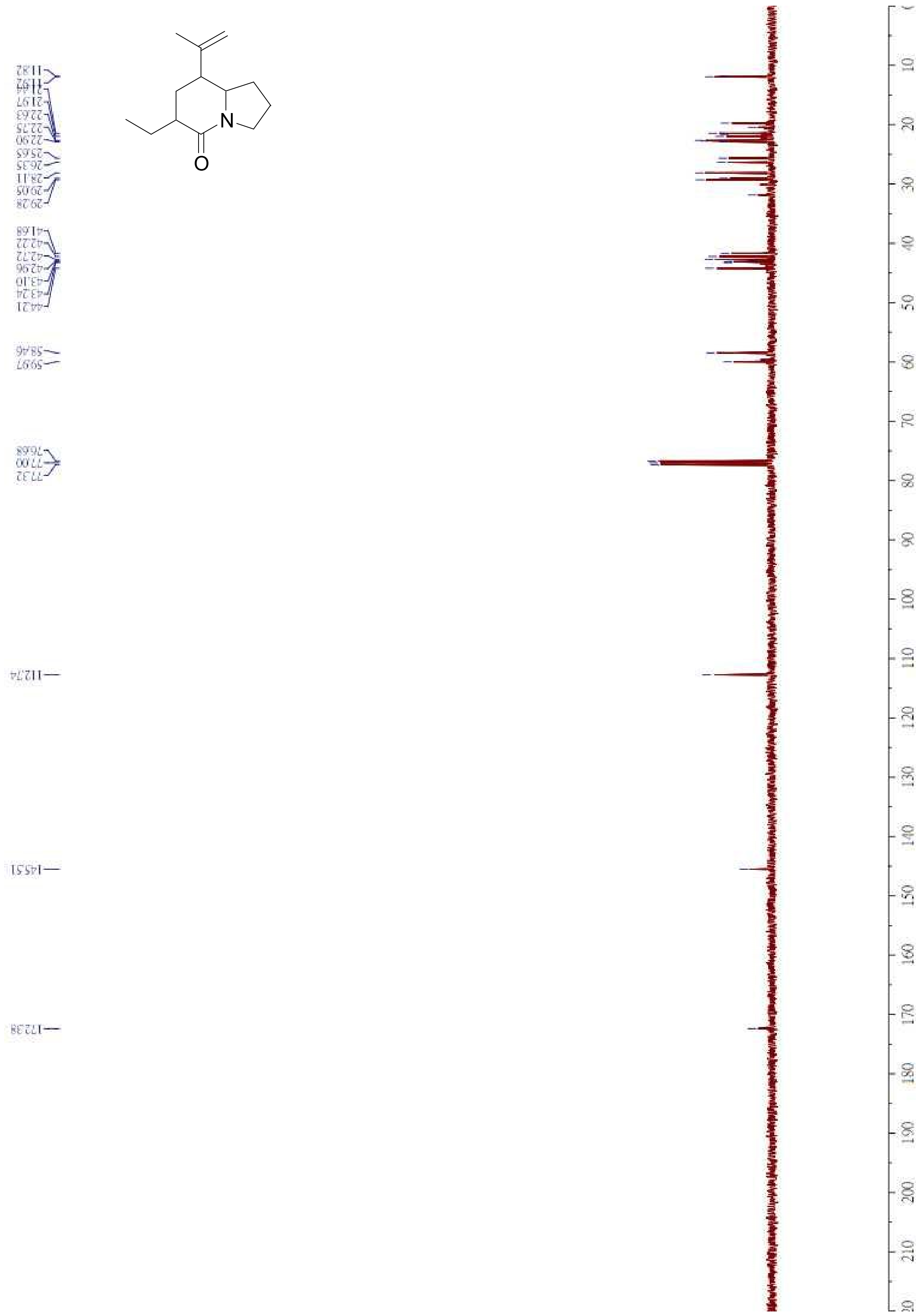

$3b_2$, ^{1}H-NMR (400MHz, $CDCl_3$)

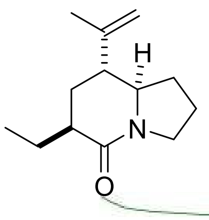

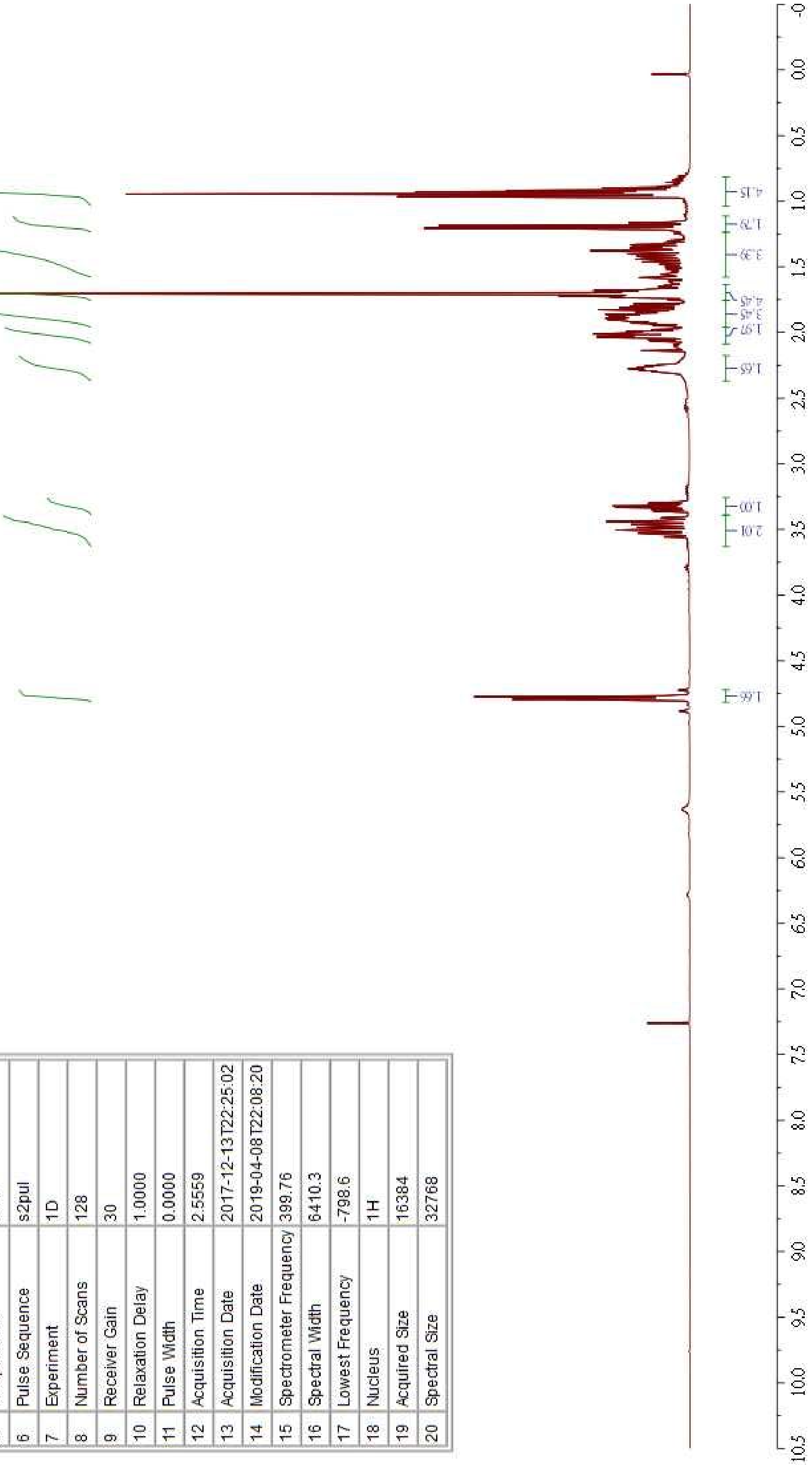

Parameters

	Parameter	Value
1	Title	wei405pf3_H_good
2	Origin	Varian
3	Spectrometer	vnmrs
4	Solvent	cdcl3
5	Temperature	20.0
6	Pulse Sequence	s2pul
7	Experiment	1D
8	Number of Scans	128
9	Receiver Gain	30
10	Relaxation Delay	1.0000
11	Pulse Width	0.0000
12	Acquisition Time	2.5559
13	Acquisition Date	2017-12-13T22:25:02
14	Modification Date	2019-04-08T22:08:20
15	Spectrometer Frequency	399.76
16	Spectral Width	6410.3
17	Lowest Frequency	-798.6
18	Nucleus	1H
19	Acquired Size	16384
20	Spectral Size	32768

3b$_2$, $^{13}C\{^1H\}$ NMR (101 MHz, CDCl$_3$)

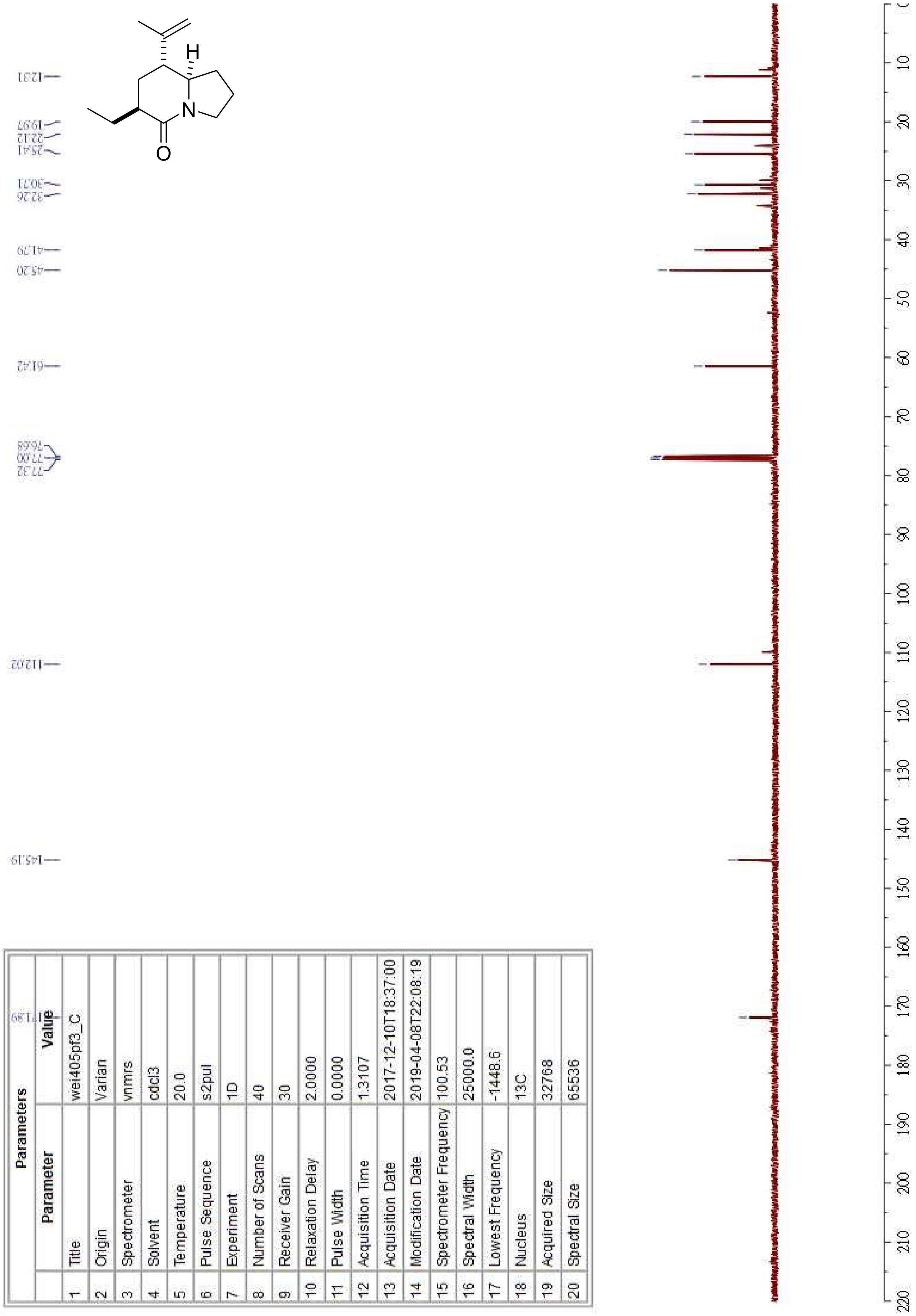

	Parameters	
	Parameter	Value
1	Title	wei405pf3_C
2	Origin	Varian
3	Spectrometer	vnmrs
4	Solvent	cdcl3
5	Temperature	20.0
6	Pulse Sequence	s2pul
7	Experiment	1D
8	Number of Scans	40
9	Receiver Gain	30
10	Relaxation Delay	2.0000
11	Pulse Width	0.0000
12	Acquisition Time	1.3107
13	Acquisition Date	2017-12-10T18:37:00
14	Modification Date	2019-04-08T22:08:19
15	Spectrometer Frequency	100.53
16	Spectral Width	25000.0
17	Lowest Frequency	-1448.6
18	Nucleus	13C
19	Acquired Size	32768
20	Spectral Size	65536

$3b_2$, DEPT (101 MHz, $CDCl_3$)

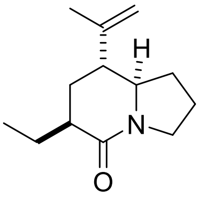

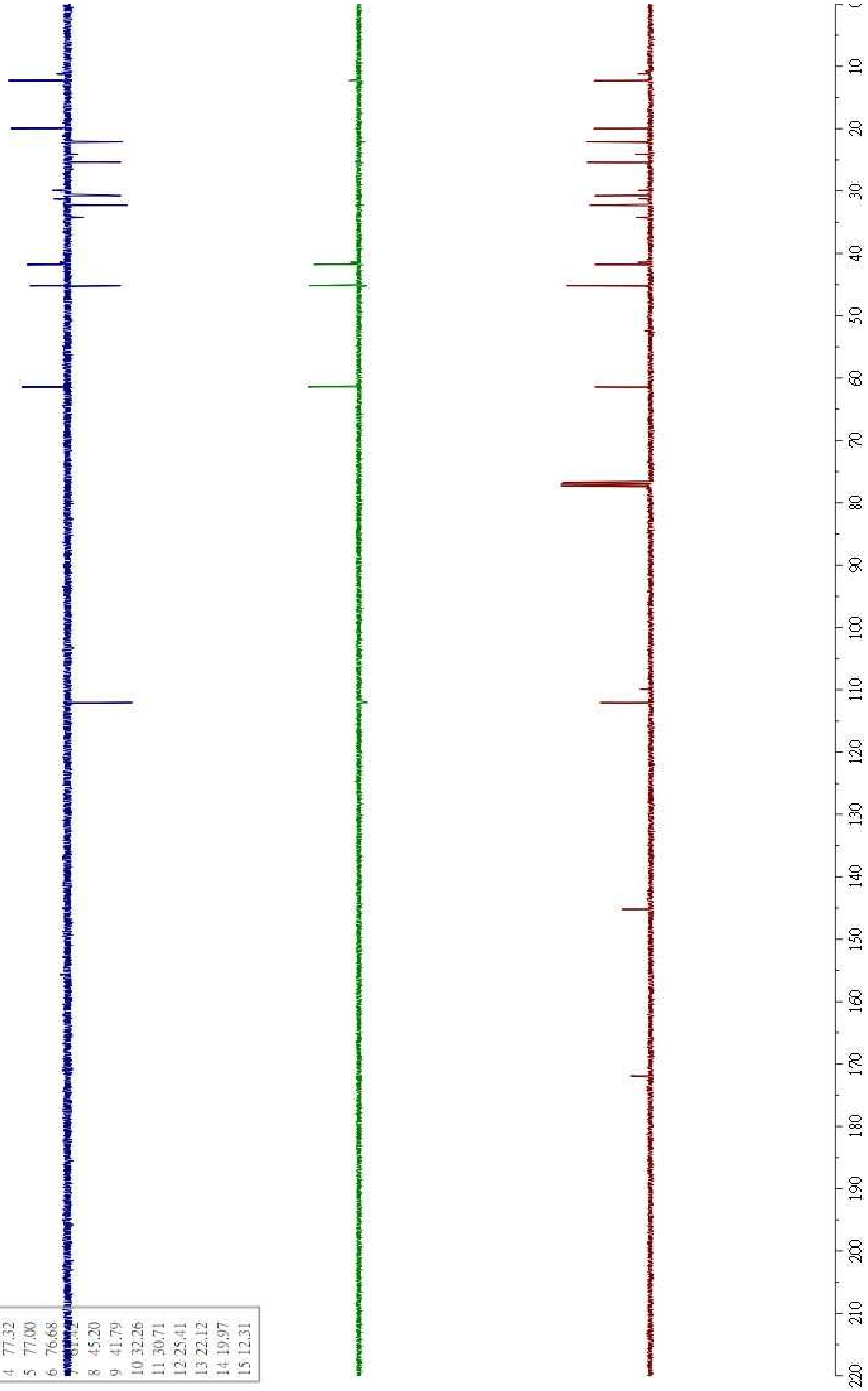

4a, ^{1}H-NMR (400MHz, $CDCl_3$)

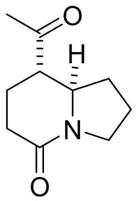

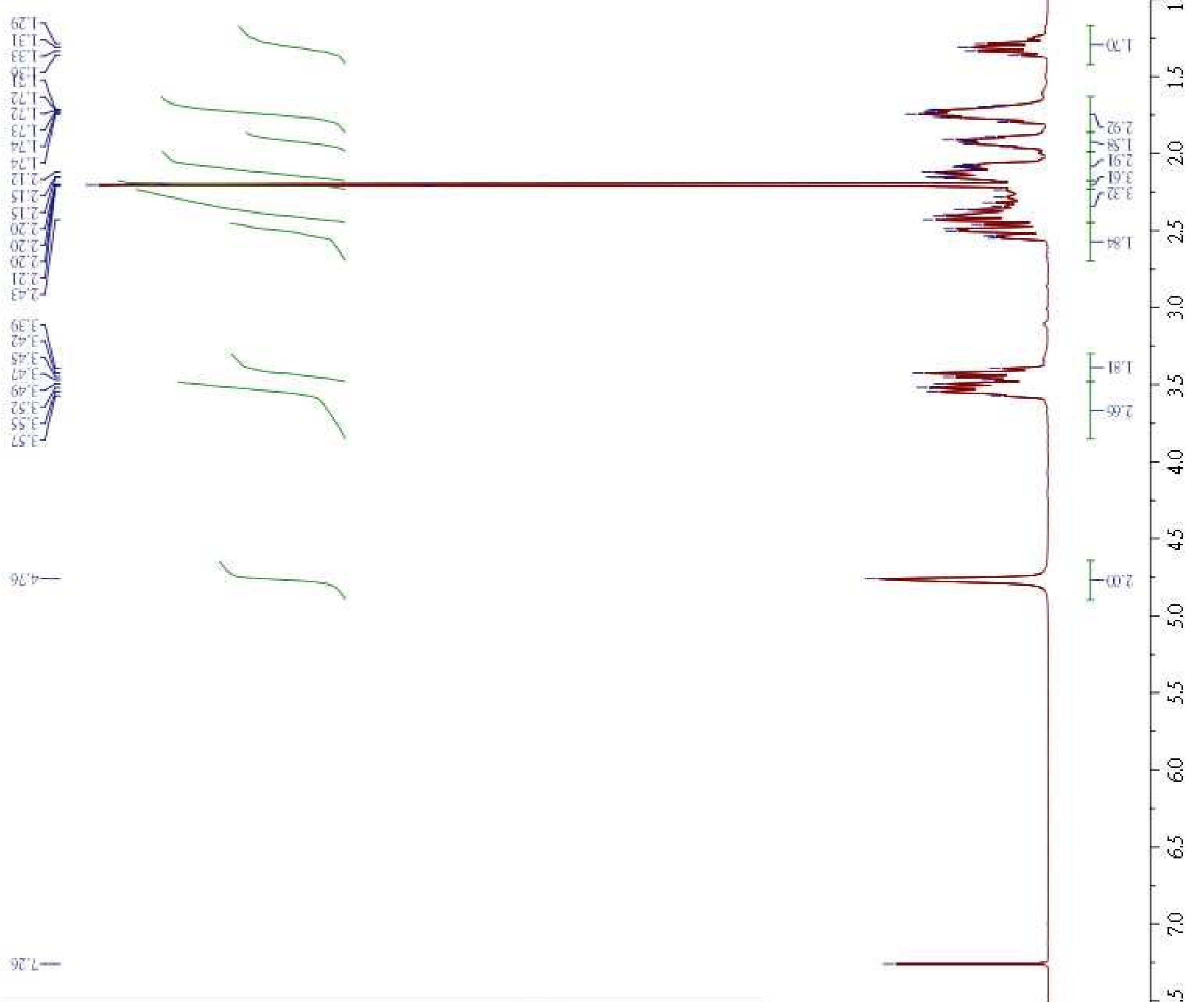

	Parameter	Value
1	Title	wei506pf1_H_good
2	Origin	Varian
3	Spectrometer	vnmrs
4	Solvent	cdcl3
5	Temperature	20.0
6	Pulse Sequence	s2pul
7	Experiment	1D
8	Number of Scans	20
9	Receiver Gain	30
10	Relaxation Delay	1.0000
11	Pulse Width	0.0000
12	Acquisition Time	2.5559
13	Acquisition Date	2018-03-22T00:54:41
14	Modification Date	2019-04-08T22:10:05
15	Spectrometer Frequency	399.76
16	Spectral Width	6410.3
17	Lowest Frequency	-798.1
18	Nucleus	1H
19	Acquired Size	16384
20	Spectral Size	32768

4a, $^{13}C\{^{1}H\}$ NMR (101 MHz, $CDCl_3$)

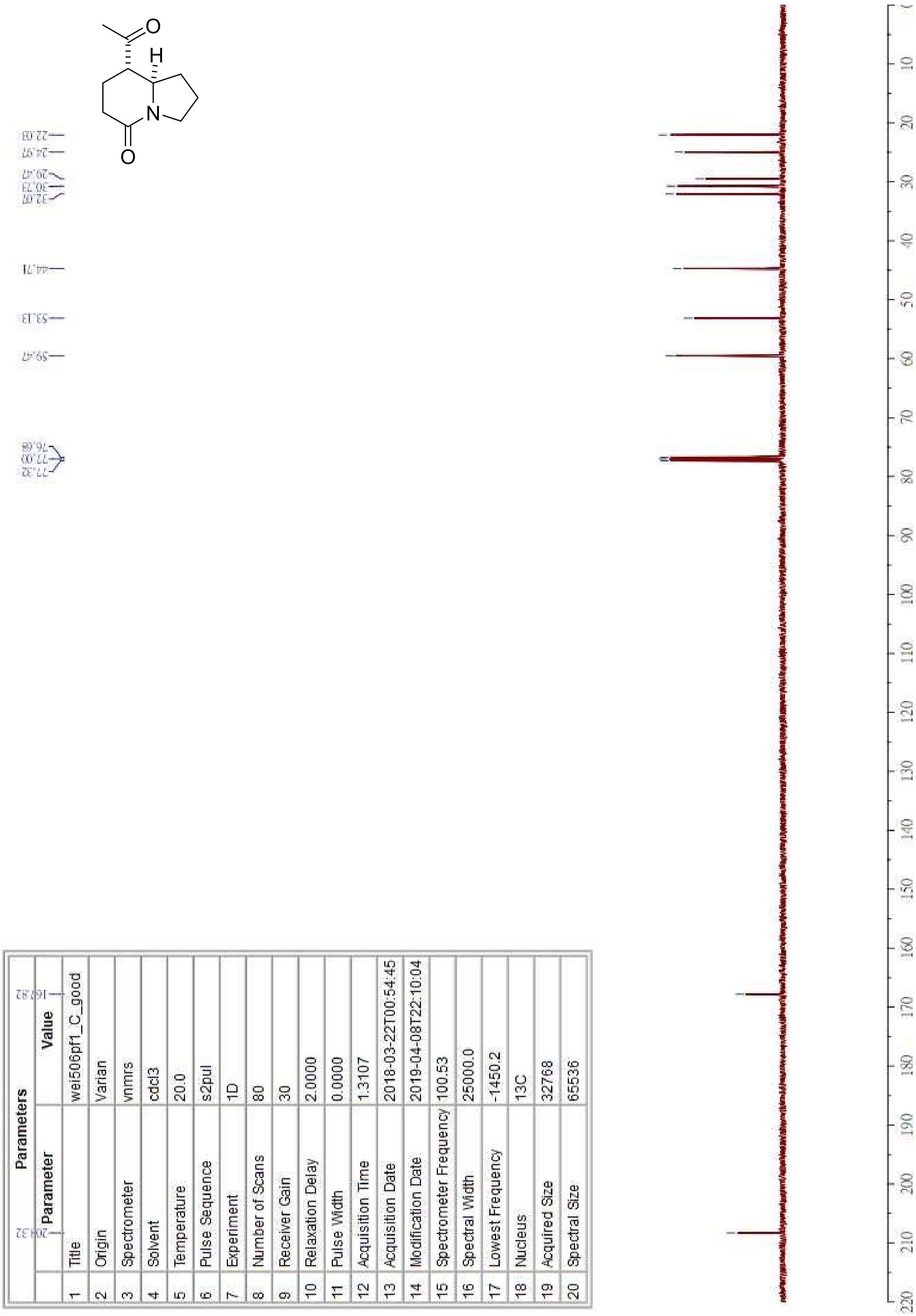

Parameters

	Parameter	Value
1	Title	wei506pf1_C_good
2	Origin	Varian
3	Spectrometer	vnmrs
4	Solvent	cdcl3
5	Temperature	20.0
6	Pulse Sequence	s2pul
7	Experiment	1D
8	Number of Scans	80
9	Receiver Gain	30
10	Relaxation Delay	2.0000
11	Pulse Width	0.0000
12	Acquisition Time	1.3107
13	Acquisition Date	2018-03-22T00:54:45
14	Modification Date	2019-04-08T22:10:04
15	Spectrometer Frequency	100.53
16	Spectral Width	25000.0
17	Lowest Frequency	-1450.2
18	Nucleus	13C
19	Acquired Size	32768
20	Spectral Size	65536

4a, DEPT (101 MHz, $CDCl_3$)

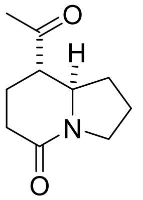

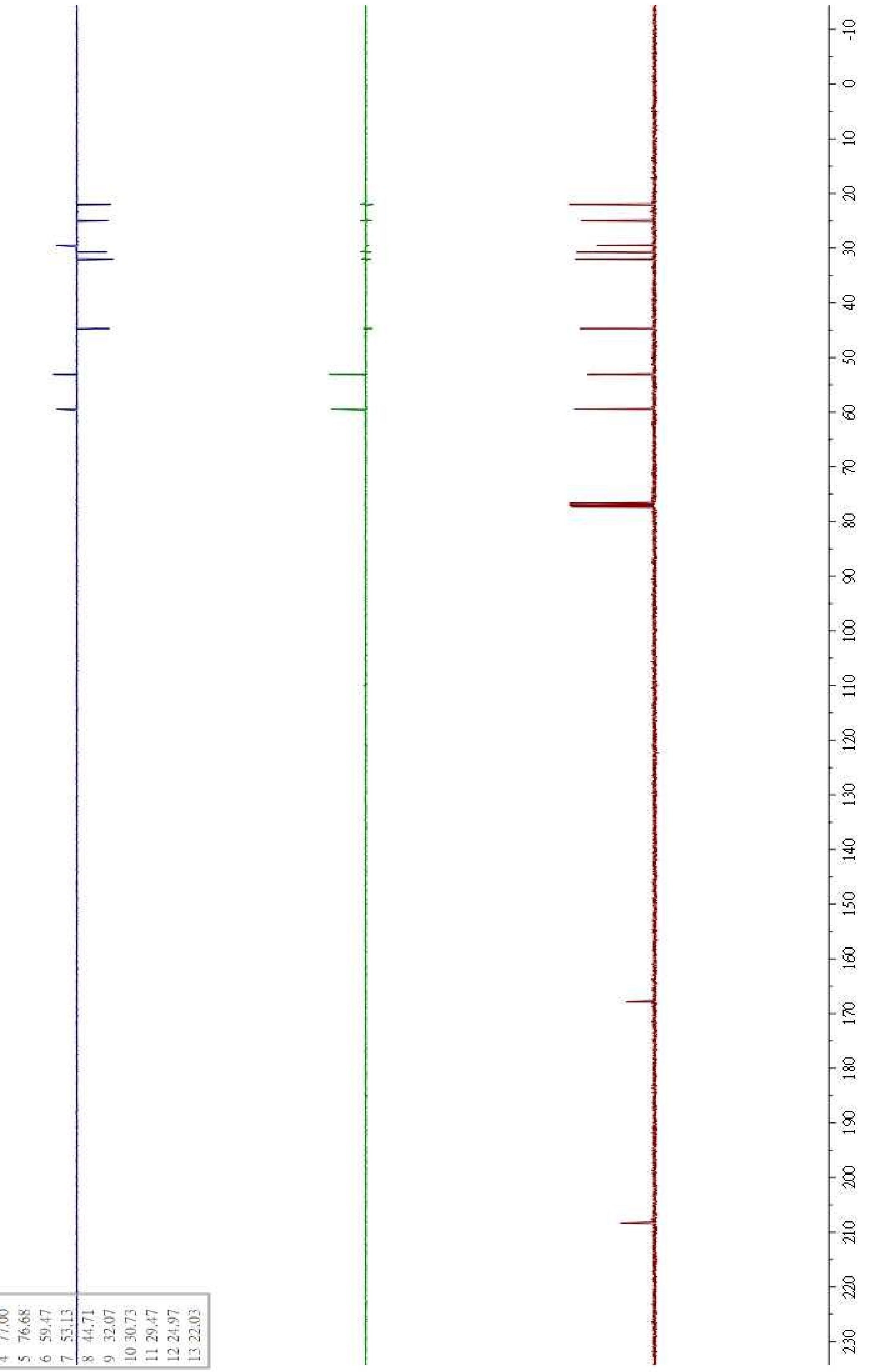

4b, ^{1}H-NMR (400MHz, $CDCl_3$)

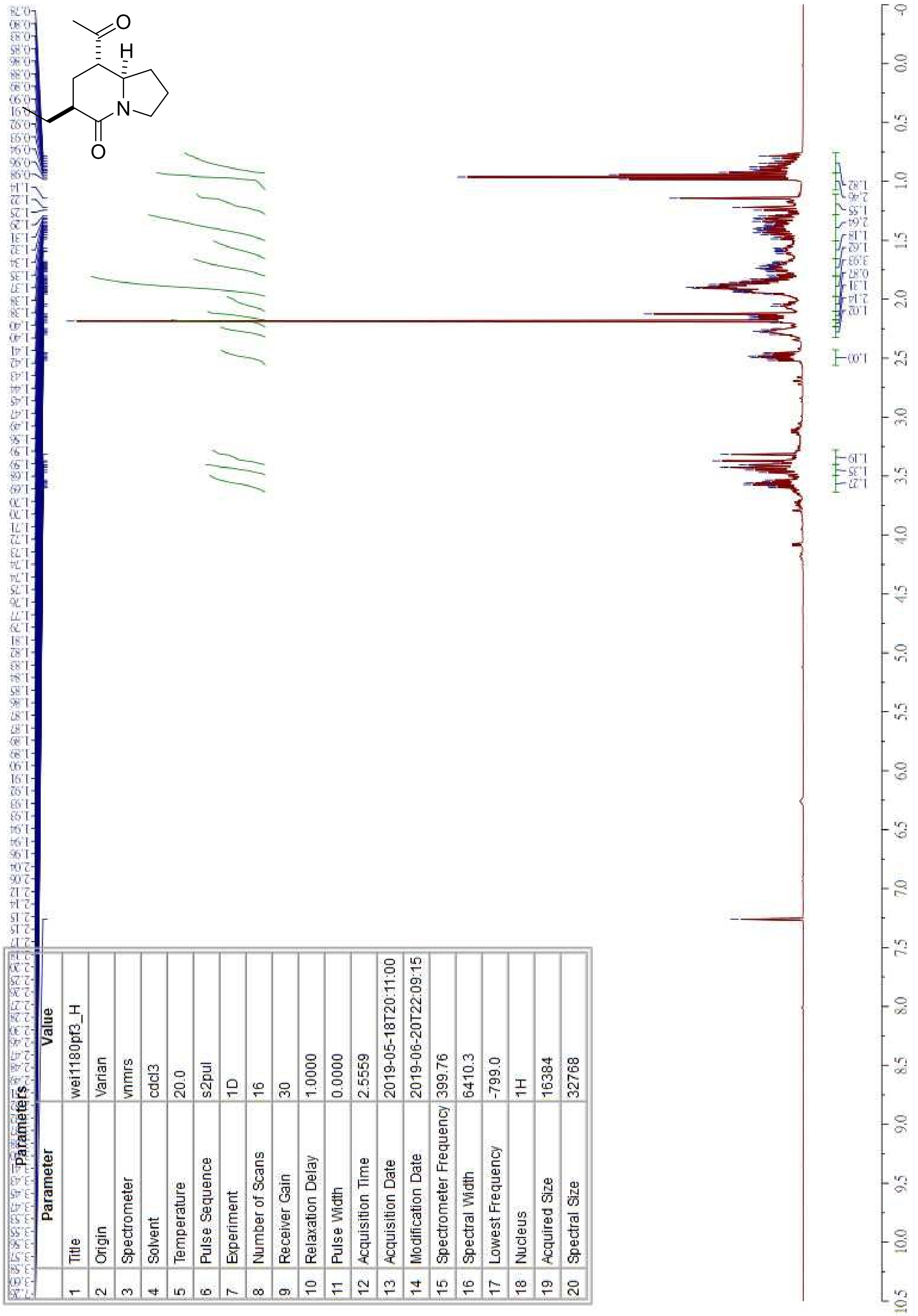

4b, $^{13}C\{^1H\}$ NMR (101 MHz, $CDCl_3$)

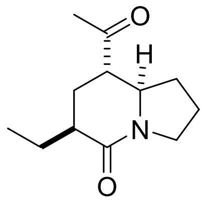

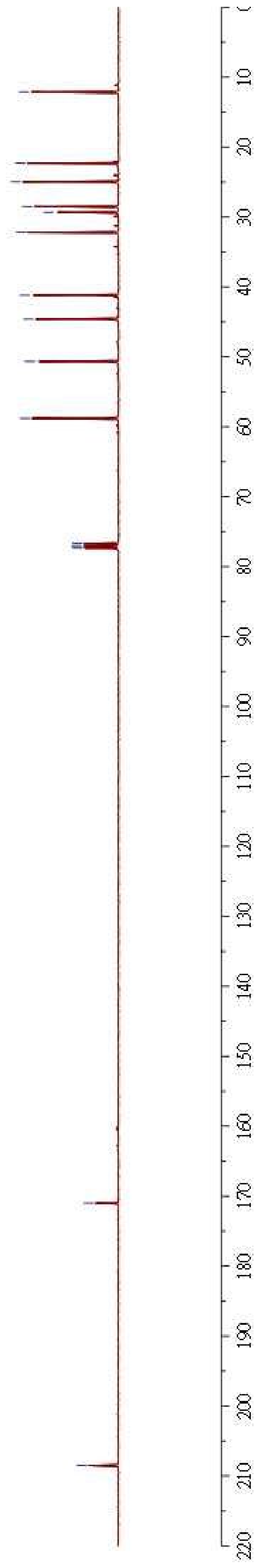

Parameters

	Parameter	Value
1	Title	wei345p_C_20171031
2	Origin	Varian
3	Spectrometer	vnmrs
4	Solvent	cdcl3
5	Temperature	25.0
6	Pulse Sequence	s2pul
7	Experiment	1D
8	Number of Scans	512
9	Receiver Gain	30
10	Relaxation Delay	2.0000
11	Pulse Width	0.0000
12	Acquisition Time	1.3107
13	Acquisition Date	2017-10-31T06:00:34
14	Modification Date	2019-04-08T22:06:36
15	Spectrometer Frequency	100.53
16	Spectral Width	25000.0
17	Lowest Frequency	-1450.9
18	Nucleus	13C
19	Acquired Size	32768
20	Spectral Size	65536

4b, DEPT (101 MHz, $CDCl_3$)

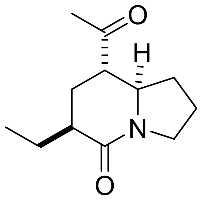

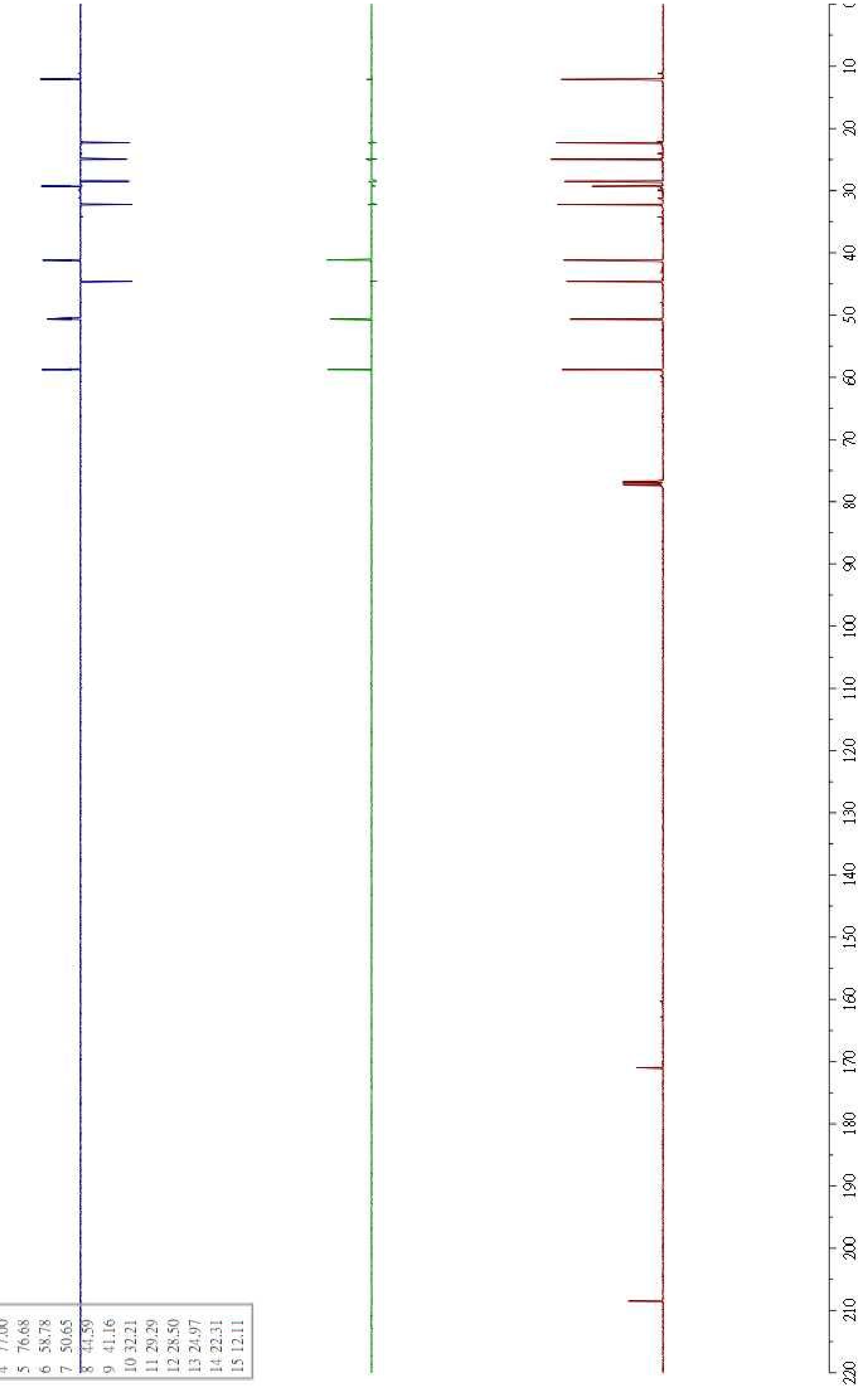

5b, ^{1}H-NMR (400MHz, $CDCl_3$)

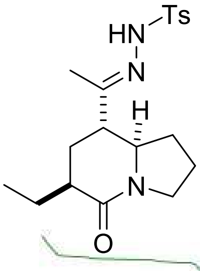

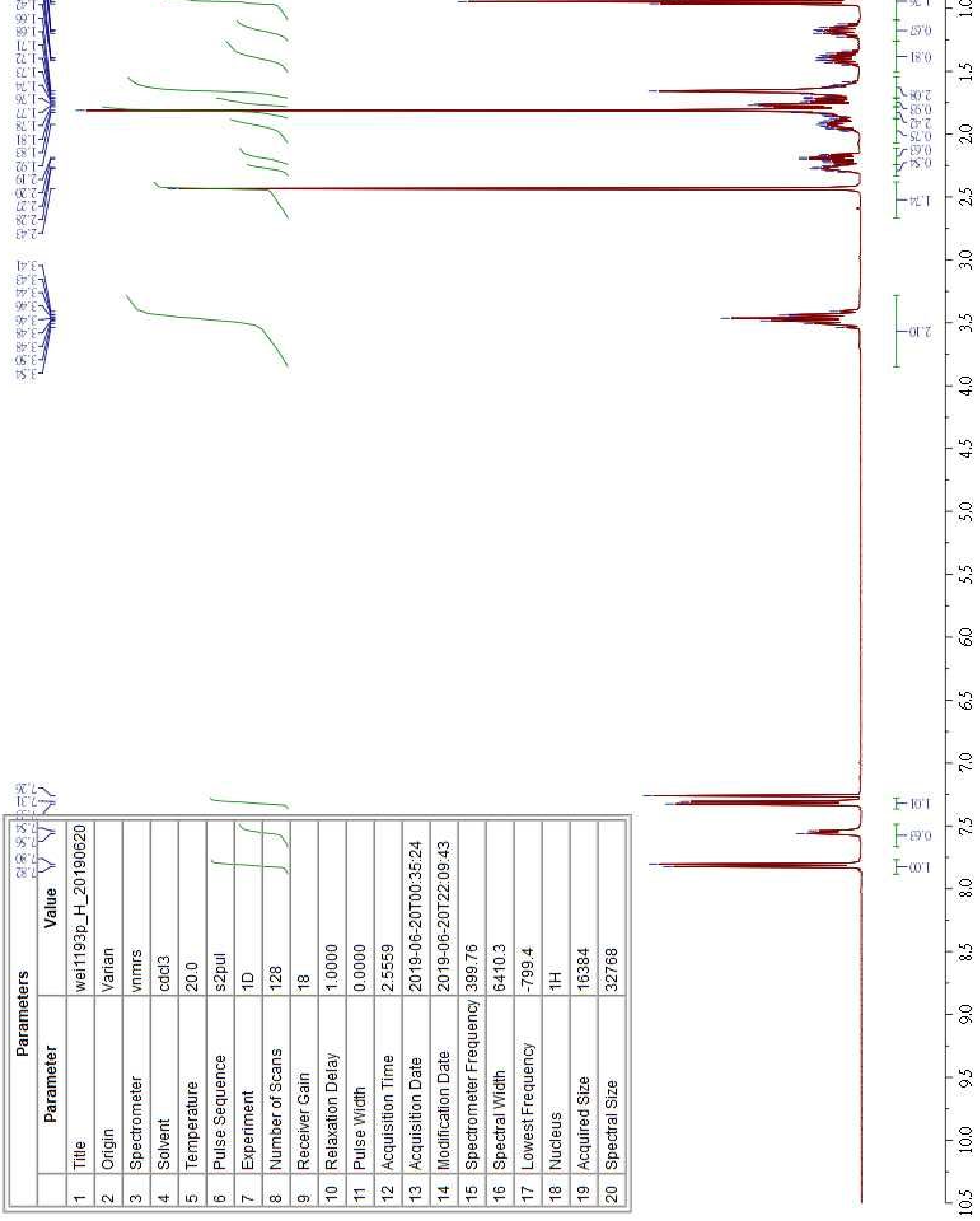

Parameters

	Parameter	Value
1	Title	wei1193p_H_20190620
2	Origin	Varian
3	Spectrometer	vnmrs
4	Solvent	cdcl3
5	Temperature	20.0
6	Pulse Sequence	s2pul
7	Experiment	1D
8	Number of Scans	128
9	Receiver Gain	18
10	Relaxation Delay	1.0000
11	Pulse Width	0.0000
12	Acquisition Time	2.5559
13	Acquisition Date	2019-06-20T00:35:24
14	Modification Date	2019-06-20T22:09:43
15	Spectrometer Frequency	399.76
16	Spectral Width	6410.3
17	Lowest Frequency	-799.4
18	Nucleus	1H
19	Acquired Size	16384
20	Spectral Size	32768

5b, $^{13}C\{^{1}H\}$ NMR (101 MHz, $CDCl_3$)

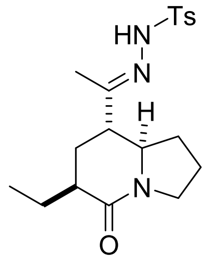

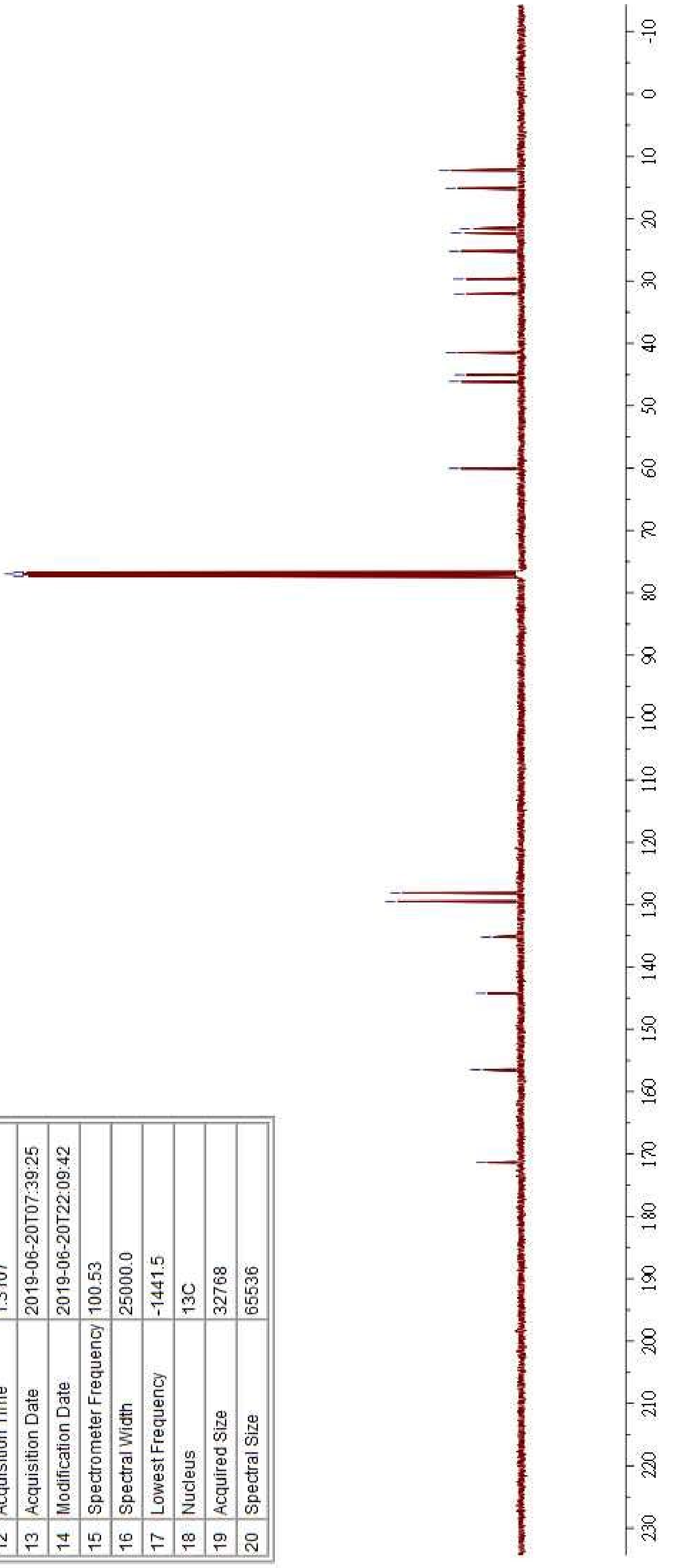

Parameters		
	Parameter	Value
1	Title	wei1193p_C_20190620
2	Origin	Varian
3	Spectrometer	vnmrs
4	Solvent	cdcl3
5	Temperature	20.0
6	Pulse Sequence	s2pul
7	Experiment	1D
8	Number of Scans	1000
9	Receiver Gain	30
10	Relaxation Delay	2.0000
11	Pulse Width	0.0000
12	Acquisition Time	1.3107
13	Acquisition Date	2019-06-20T07:39:25
14	Modification Date	2019-06-20T22:09:42
15	Spectrometer Frequency	100.53
16	Spectral Width	25000.0
17	Lowest Frequency	-1441.5
18	Nucleus	13C
19	Acquired Size	32768
20	Spectral Size	65536

5b, DEPT (101 MHz, $CDCl_3$)

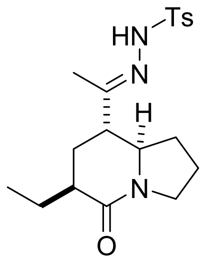

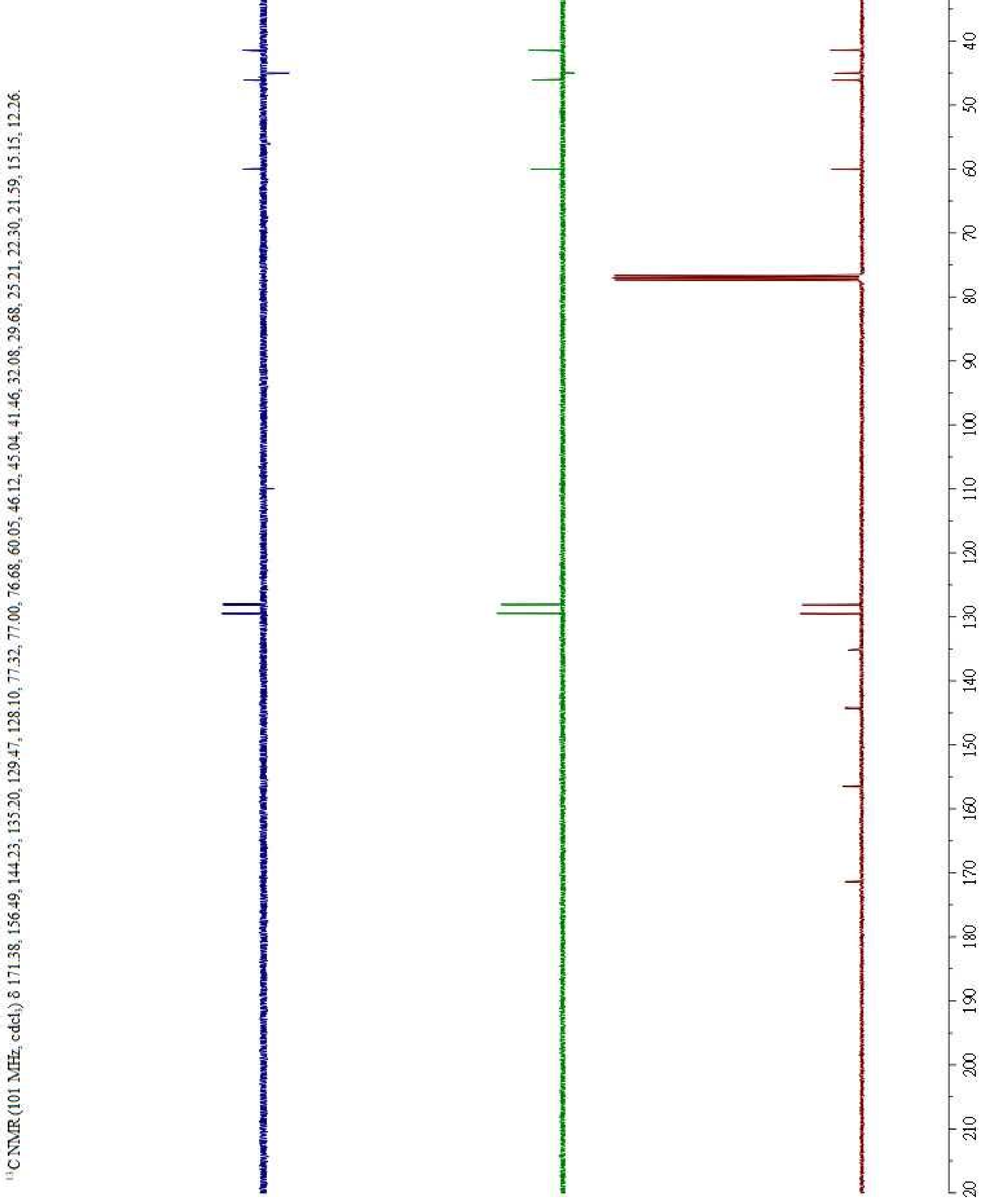

6a, ^{1}H-NMR (400MHz, $CDCl_3$)

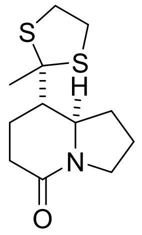

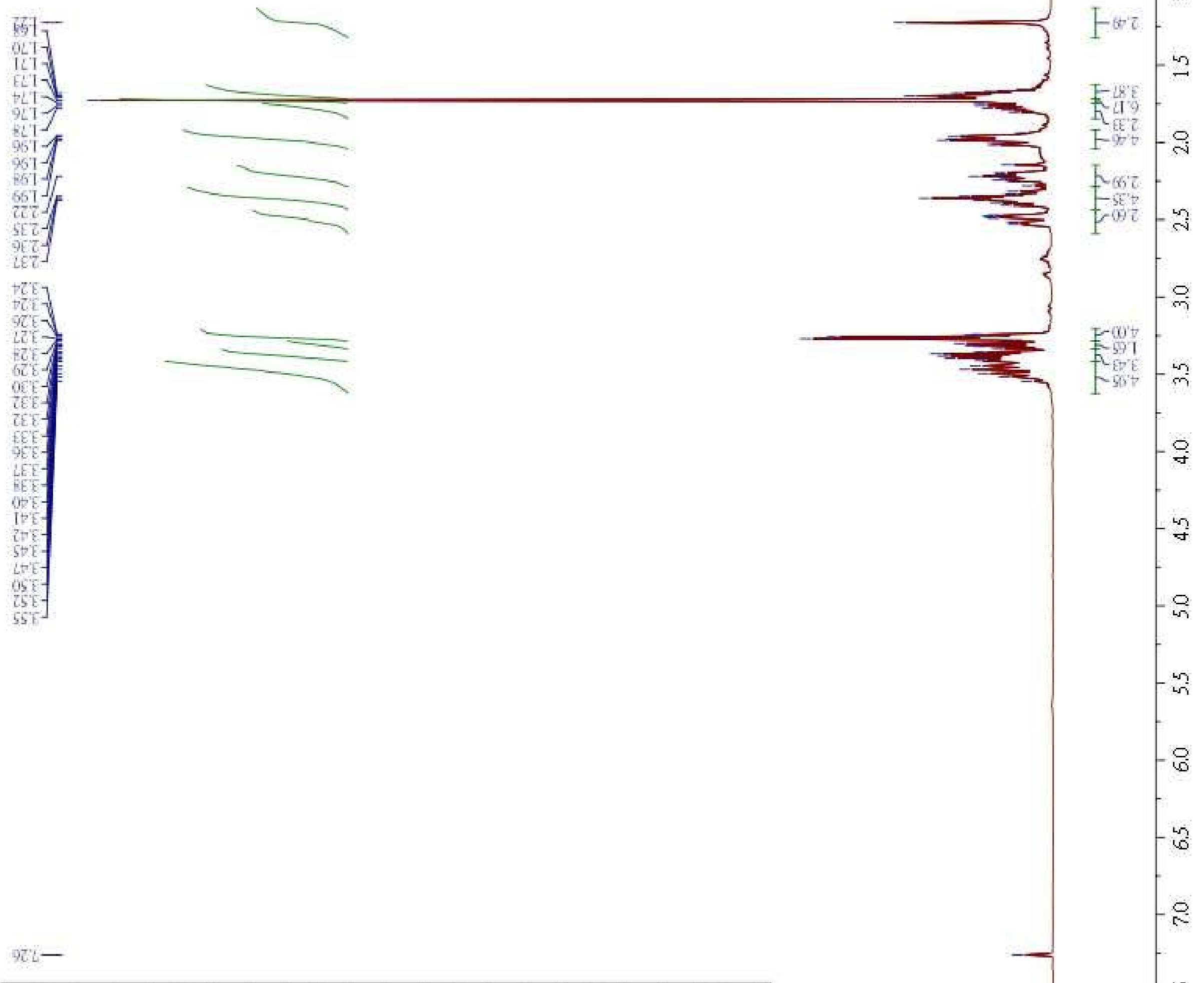

Parameters		
	Parameter	Value
1	Title	wei354p_H_20171025
2	Origin	Varian
3	Spectrometer	vnmrs
4	Solvent	cdcl3
5	Temperature	25.0
6	Pulse Sequence	s2pul
7	Experiment	1D
8	Number of Scans	20
9	Receiver Gain	30
10	Relaxation Delay	1.0000
11	Pulse Width	0.0000
12	Acquisition Time	2.5559
13	Acquisition Date	2017-10-25T13:35:49
14	Modification Date	2019-04-08T22:06:59
15	Spectrometer Frequency	399.76
16	Spectral Width	6410.3
17	Lowest Frequency	-798.6
18	Nucleus	1H
19	Acquired Size	16384
20	Spectral Size	32768

6a, $^{13}C\{^1H\}$ NMR (101 MHz, $CDCl_3$)

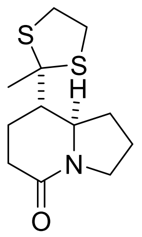

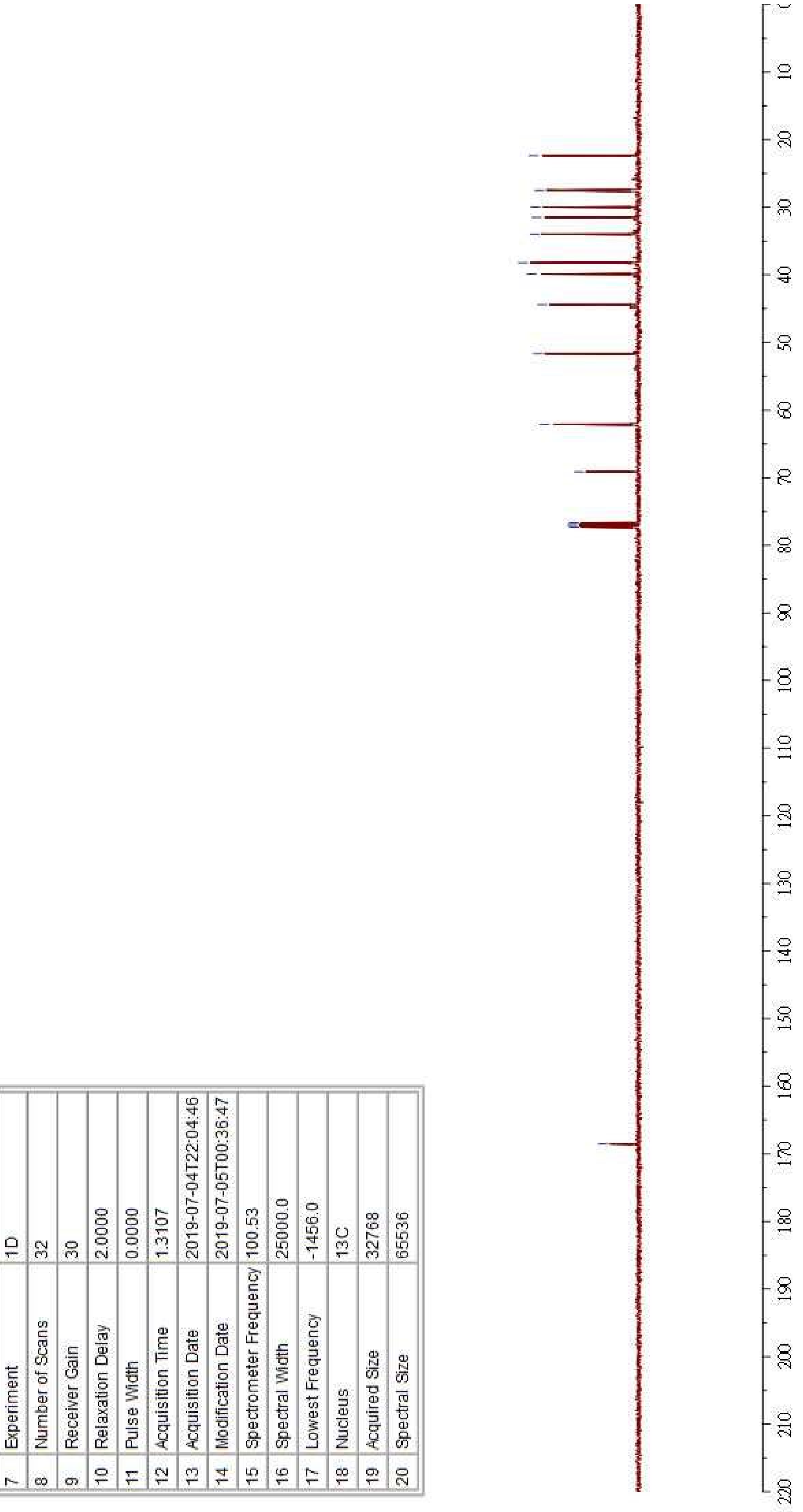

Parameters

	Parameter	Value
1	Title	wei1253p_C
2	Origin	Varian
3	Spectrometer	vnmrs
4	Solvent	cdcl3
5	Temperature	20.0
6	Pulse Sequence	s2pul
7	Experiment	1D
8	Number of Scans	32
9	Receiver Gain	30
10	Relaxation Delay	2.0000
11	Pulse Width	0.0000
12	Acquisition Time	1.3107
13	Acquisition Date	2019-07-04T22:04:46
14	Modification Date	2019-07-05T00:36:47
15	Spectrometer Frequency	100.53
16	Spectral Width	25000.0
17	Lowest Frequency	-1456.0
18	Nucleus	13C
19	Acquired Size	32768
20	Spectral Size	65536

6a, DEPT (101 MHz, $CDCl_3$)

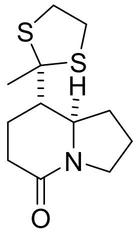

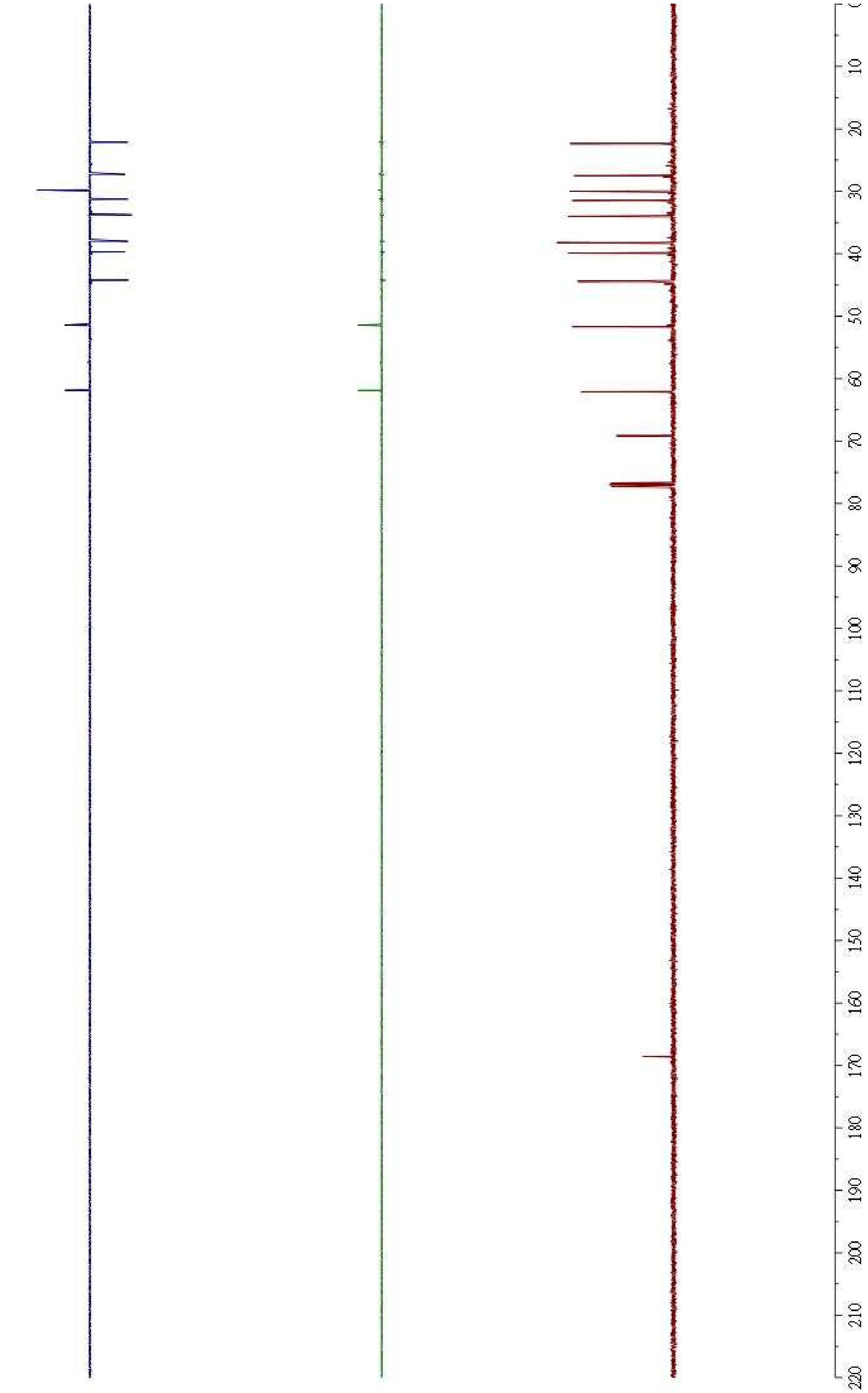

6b, ^{1}H-NMR (400MHz, $CDCl_3$)

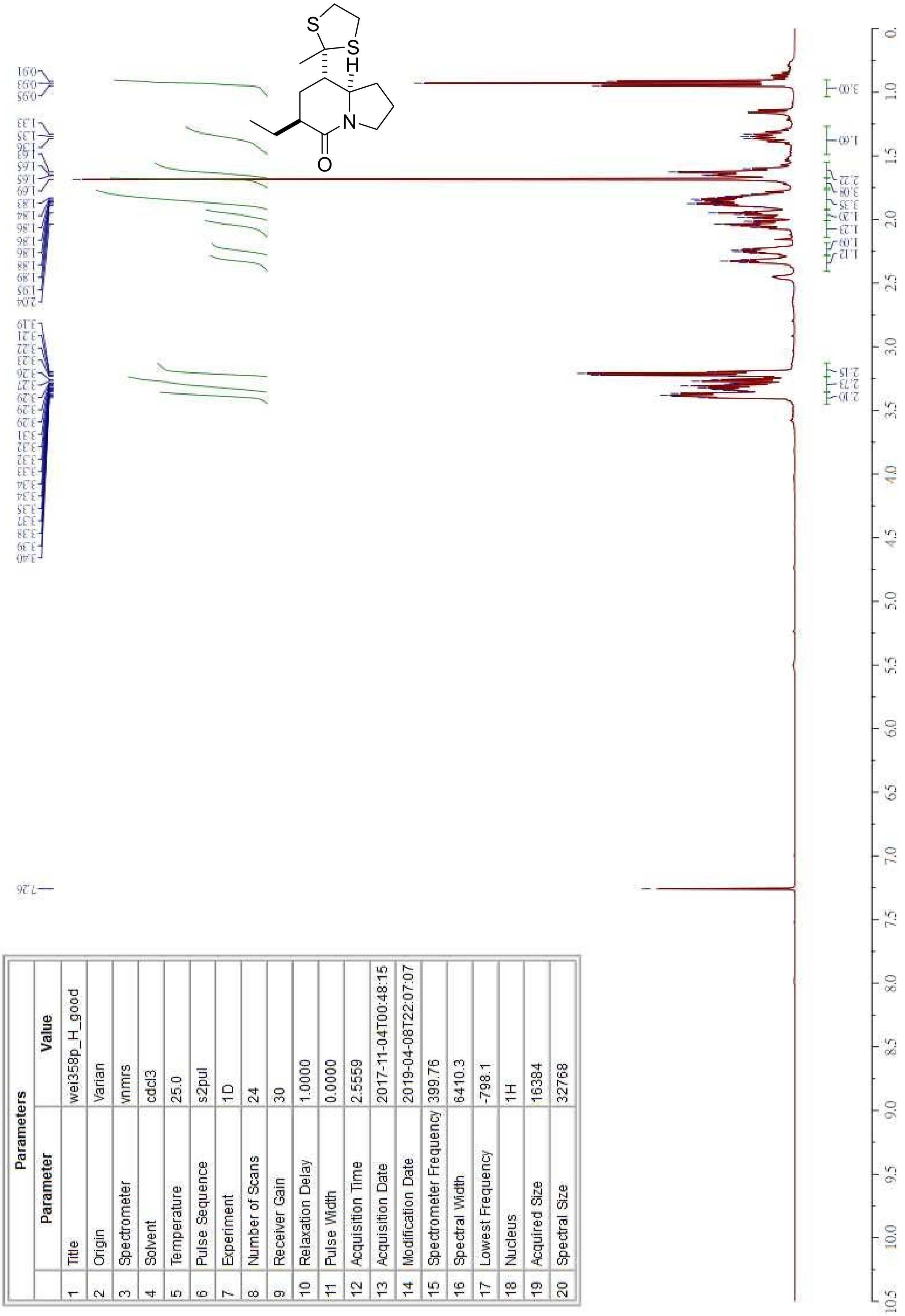

Parameters

	Parameter	Value
1	Title	wei358p_H_good
2	Origin	Varian
3	Spectrometer	vnmrs
4	Solvent	cdcl3
5	Temperature	25.0
6	Pulse Sequence	s2pul
7	Experiment	1D
8	Number of Scans	24
9	Receiver Gain	30
10	Relaxation Delay	1.0000
11	Pulse Width	0.0000
12	Acquisition Time	2.5559
13	Acquisition Date	2017-11-04T00:48:15
14	Modification Date	2019-04-08T22:07:07
15	Spectrometer Frequency	399.76
16	Spectral Width	6410.3
17	Lowest Frequency	-798.1
18	Nucleus	1H
19	Acquired Size	16384
20	Spectral Size	32768

6b, $^{13}C\{^{1}H\}$ NMR (101 MHz, $CDCl_3$)

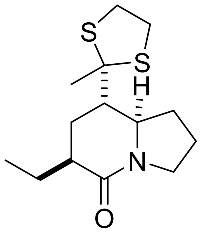

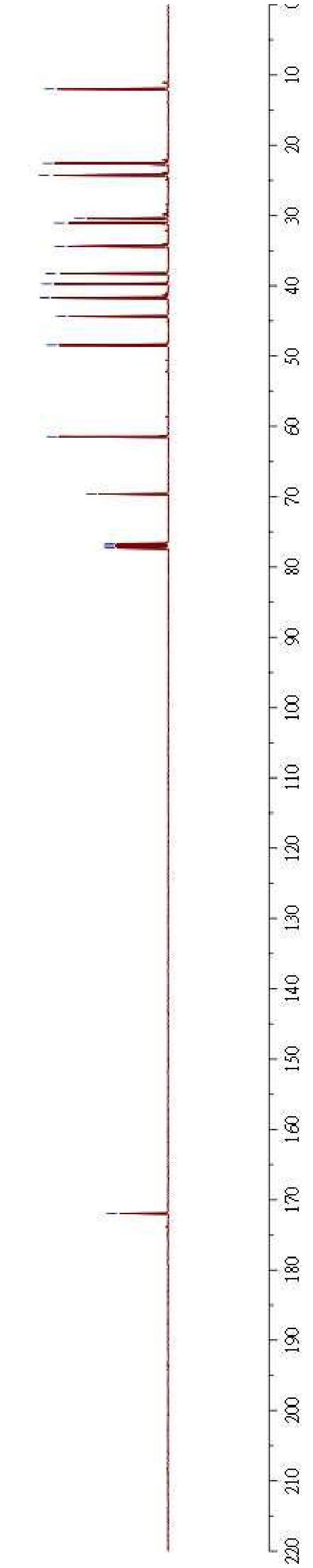

Parameters

	Parameter	Value
1	Title	wei358p_C_good
2	Origin	Varian
3	Spectrometer	vnmrs
4	Solvent	cdcl3
5	Temperature	25.0
6	Pulse Sequence	s2pul
7	Experiment	1D
8	Number of Scans	512
9	Receiver Gain	30
10	Relaxation Delay	2.0000
11	Pulse Width	0.0000
12	Acquisition Time	1.3107
13	Acquisition Date	2017-11-04T07:39:21
14	Modification Date	2019-04-08T22:07:07
15	Spectrometer Frequency	100.53
16	Spectral Width	25000.0
17	Lowest Frequency	-1458.1
18	Nucleus	13C
19	Acquired Size	32768
20	Spectral Size	65536

6b, DEPT (101 MHz, $CDCl_3$)

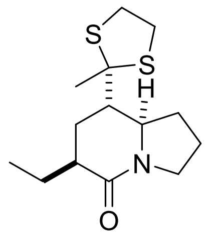

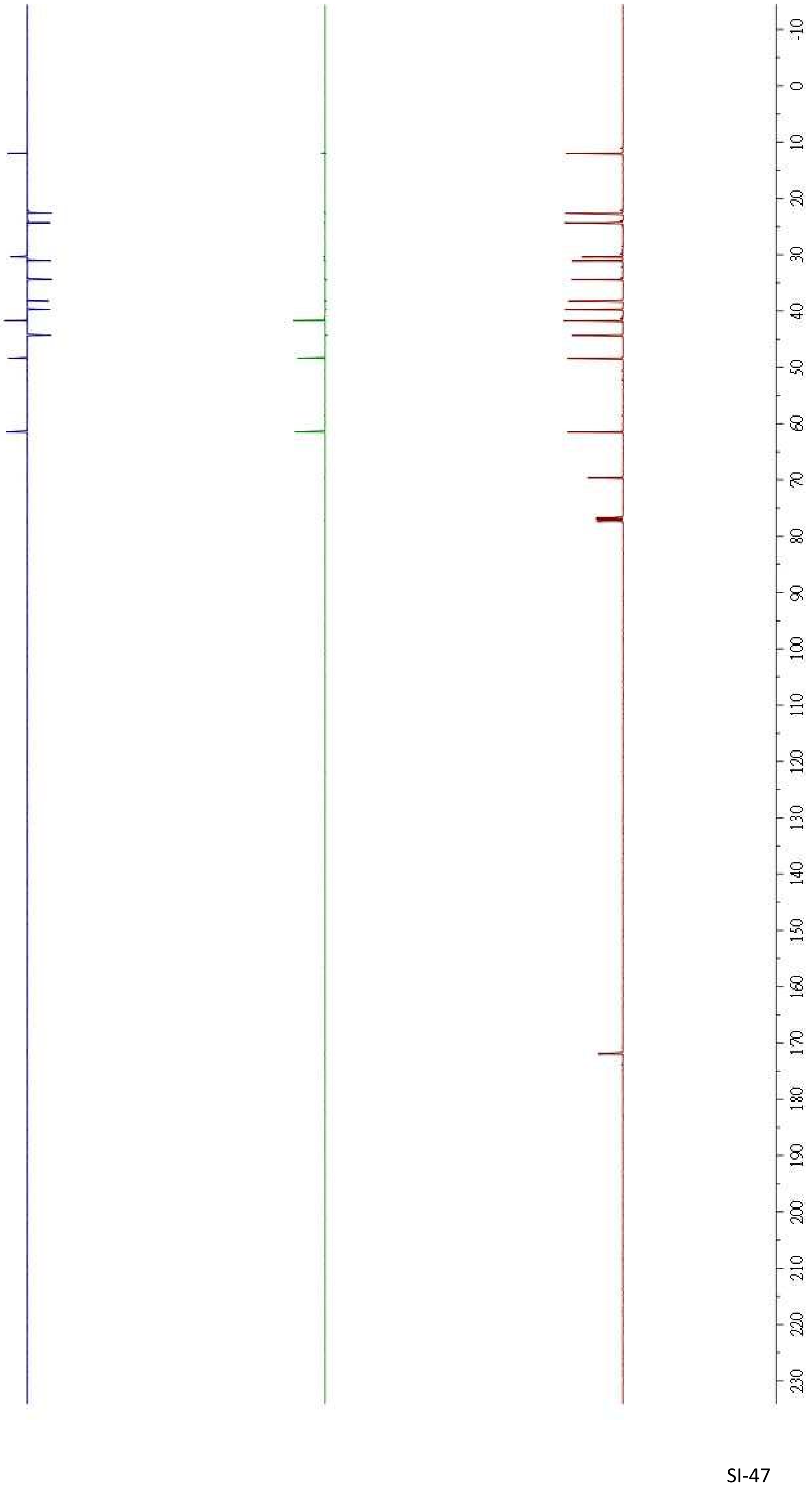

^{13}C NMR (101 MHz, cdcl$_3$) δ 171.94, 77.32, 77.00, 76.68, 69.61, 61.48, 48.44, 44.34, 41.72, 39.75, 38.25, 34.40, 31.10, 30.41, 24.32, 22.61, 12.01.

7a, ^{1}H-NMR (400MHz, $CDCl_3$)

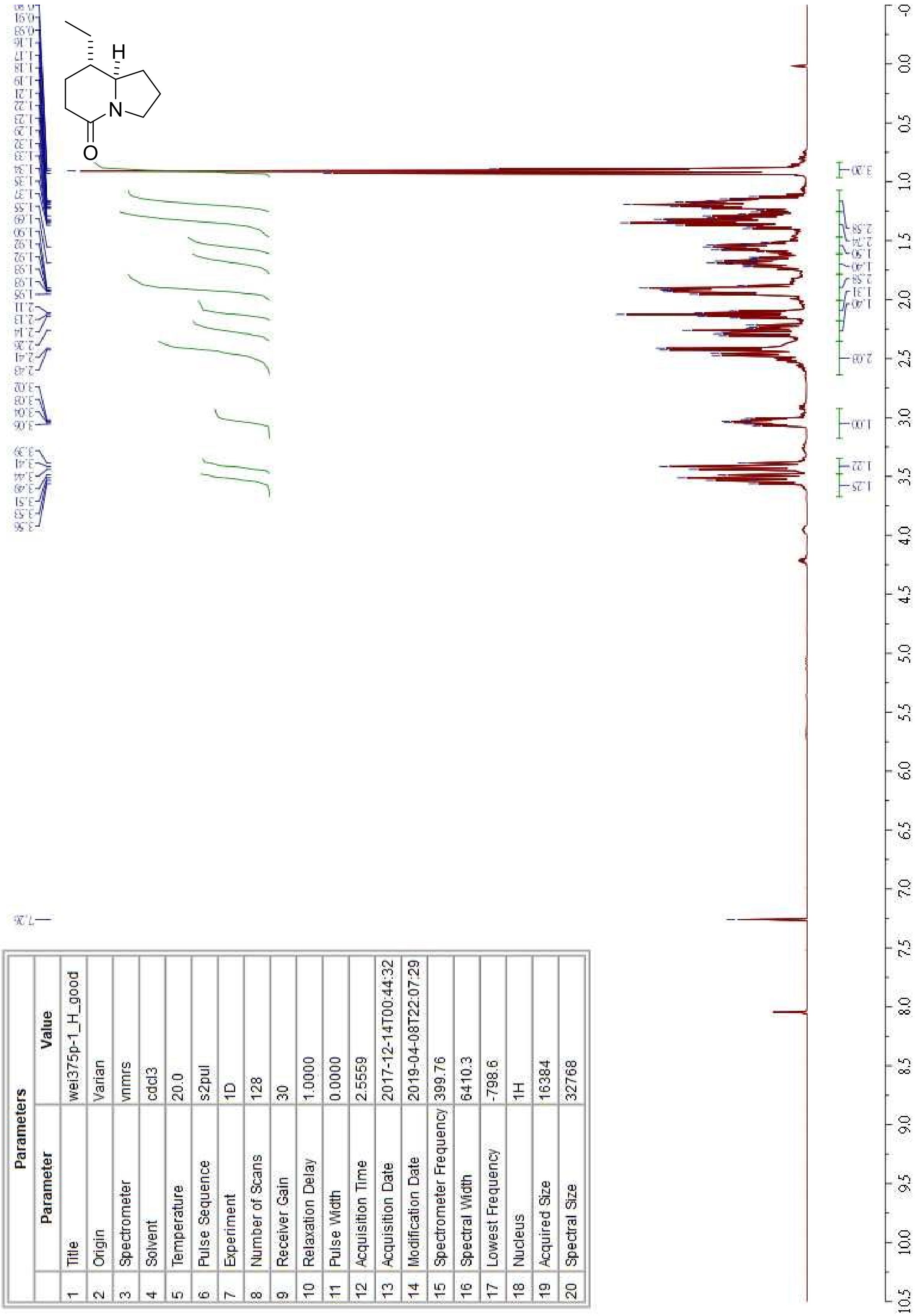

	Parameter	Value
1	Title	wei375p-1_H_good
2	Origin	Varian
3	Spectrometer	vnmrs
4	Solvent	cdcl3
5	Temperature	20.0
6	Pulse Sequence	s2pul
7	Experiment	1D
8	Number of Scans	128
9	Receiver Gain	30
10	Relaxation Delay	1.0000
11	Pulse Width	0.0000
12	Acquisition Time	2.5559
13	Acquisition Date	2017-12-14T00:44:32
14	Modification Date	2019-04-08T22:07:29
15	Spectrometer Frequency	399.76
16	Spectral Width	6410.3
17	Lowest Frequency	-798.6
18	Nucleus	1H
19	Acquired Size	16384
20	Spectral Size	32768

7a, $^{13}C\{^{1}H\}$ NMR (101 MHz, $CDCl_3$)

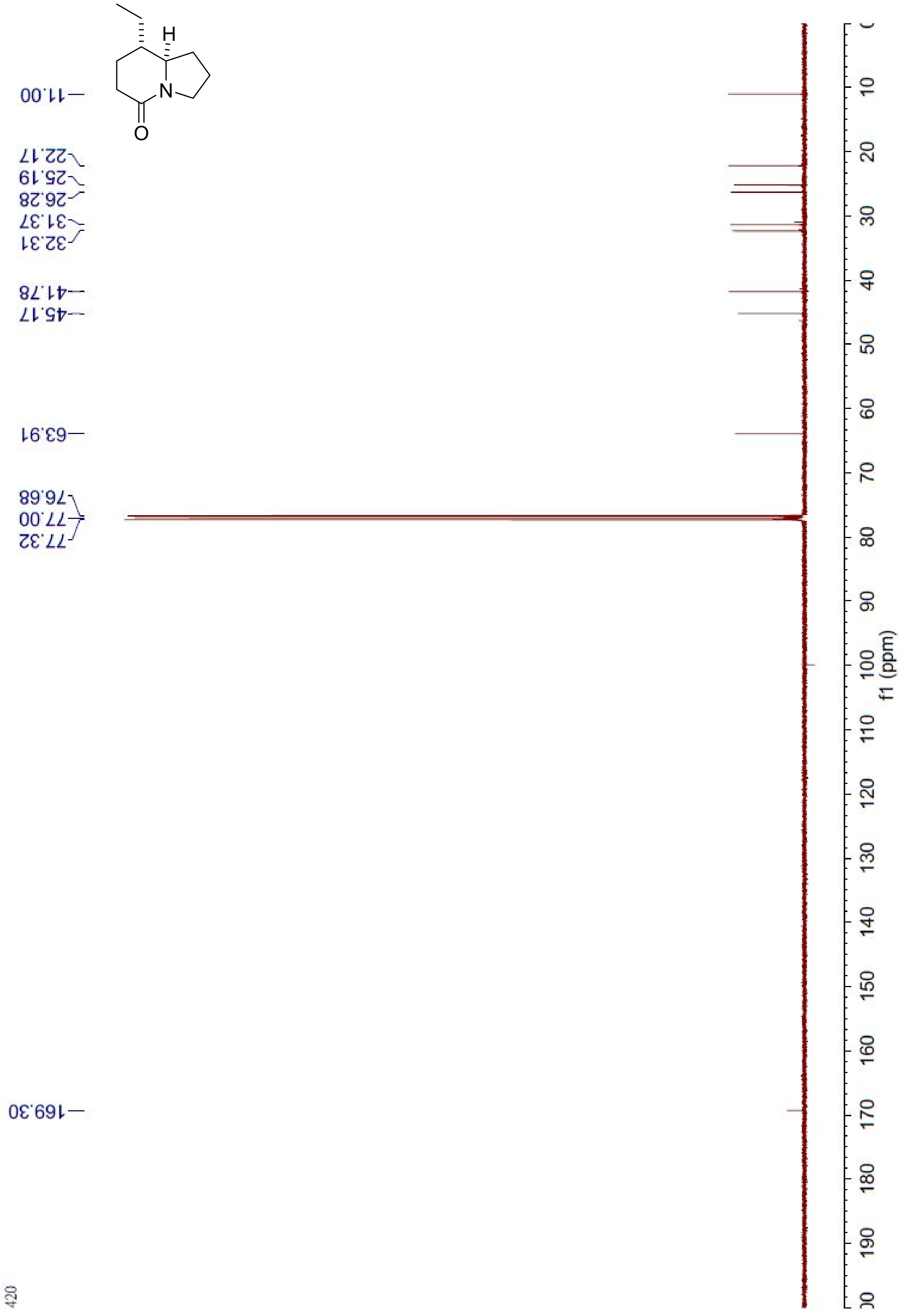

7a, DEPT (101 MHz, $CDCl_3$)

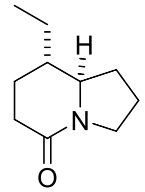

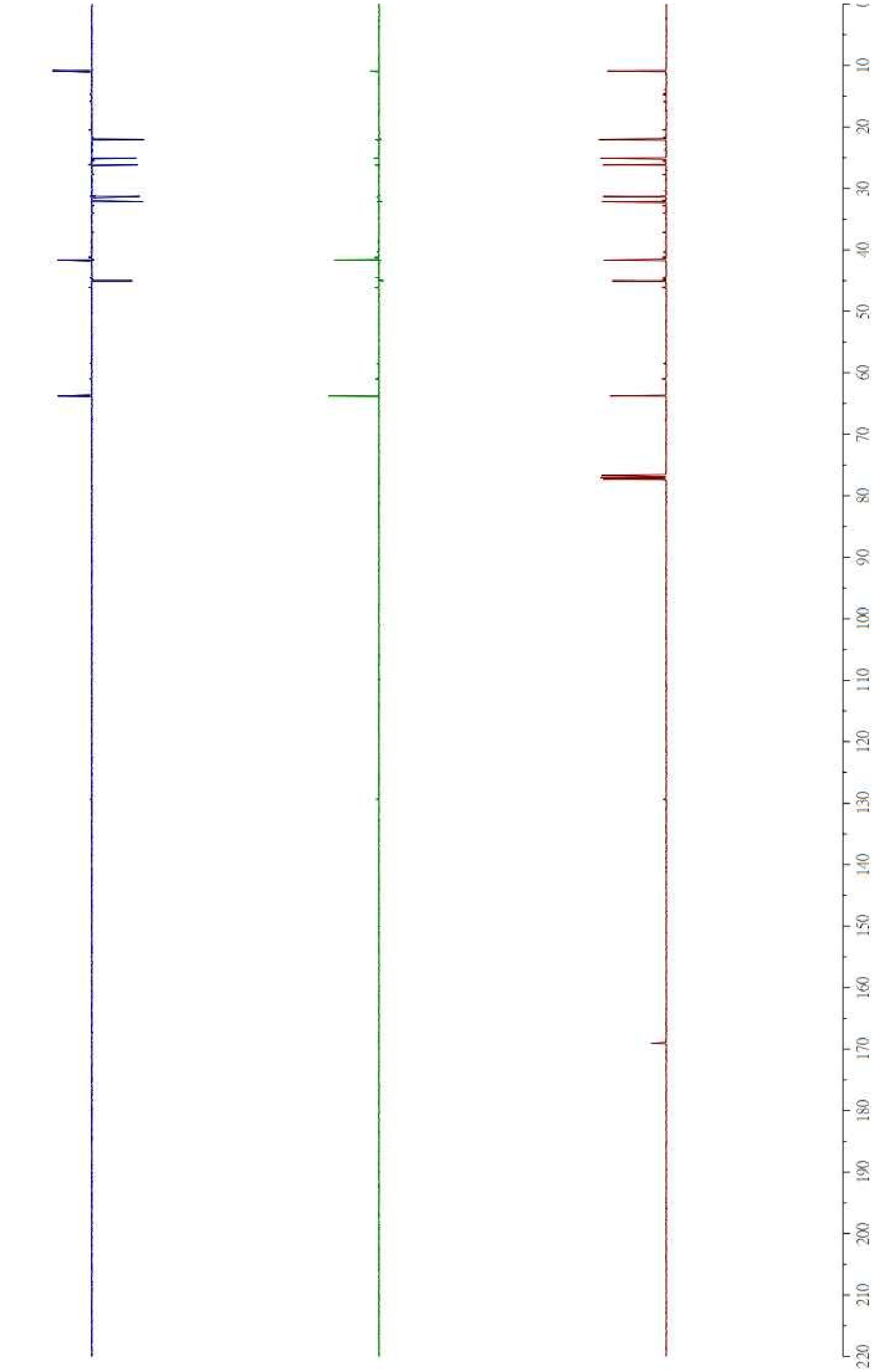

7b, ^{1}H-NMR (400MHz, $CDCl_3$)

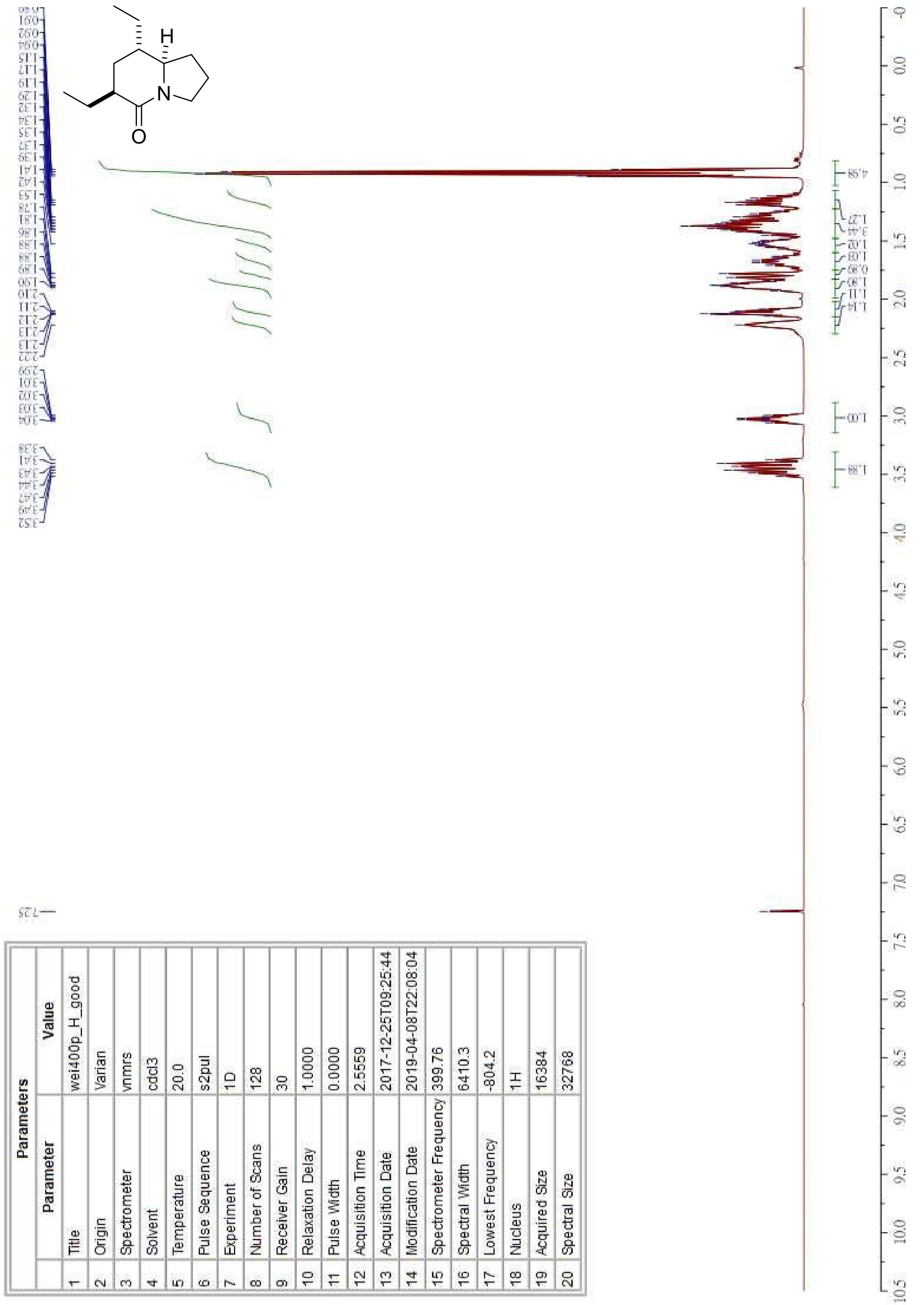

	Parameter	Value
1	Title	wei400p_H_good
2	Origin	Varian
3	Spectrometer	vnmrs
4	Solvent	cdcl3
5	Temperature	20.0
6	Pulse Sequence	s2pul
7	Experiment	1D
8	Number of Scans	128
9	Receiver Gain	30
10	Relaxation Delay	1.0000
11	Pulse Width	0.0000
12	Acquisition Time	2.5559
13	Acquisition Date	2017-12-25T09:25:44
14	Modification Date	2019-04-08T22:08:04
15	Spectrometer Frequency	399.76
16	Spectral Width	6410.3
17	Lowest Frequency	-804.2
18	Nucleus	1H
19	Acquired Size	16384
20	Spectral Size	32768

7b, $^{13}C\{^{1}H\}$ NMR (101 MHz, $CDCl_3$)

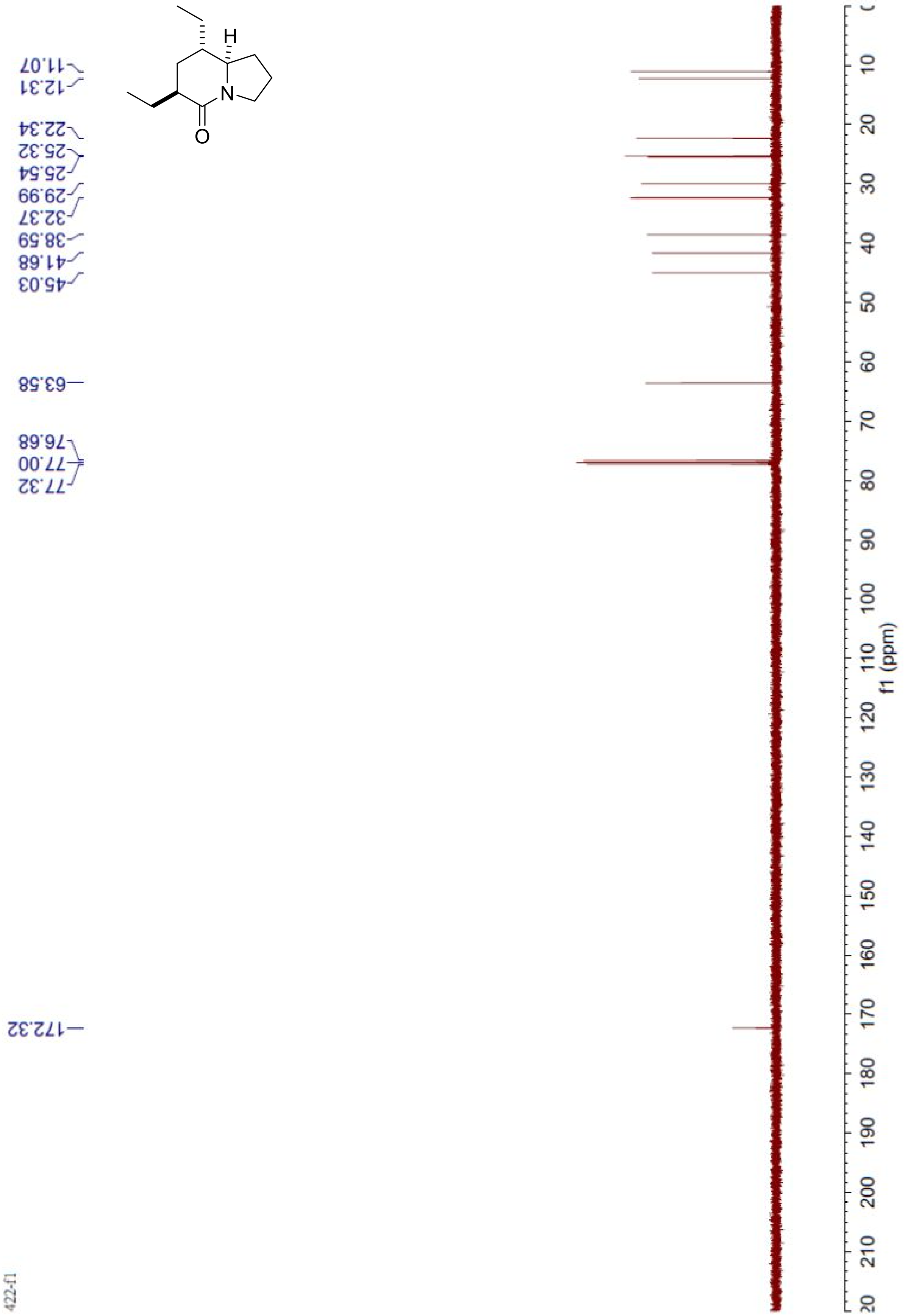

7b, DEPT (101 MHz, $CDCl_3$)

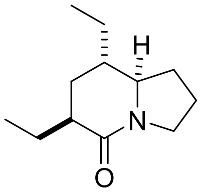

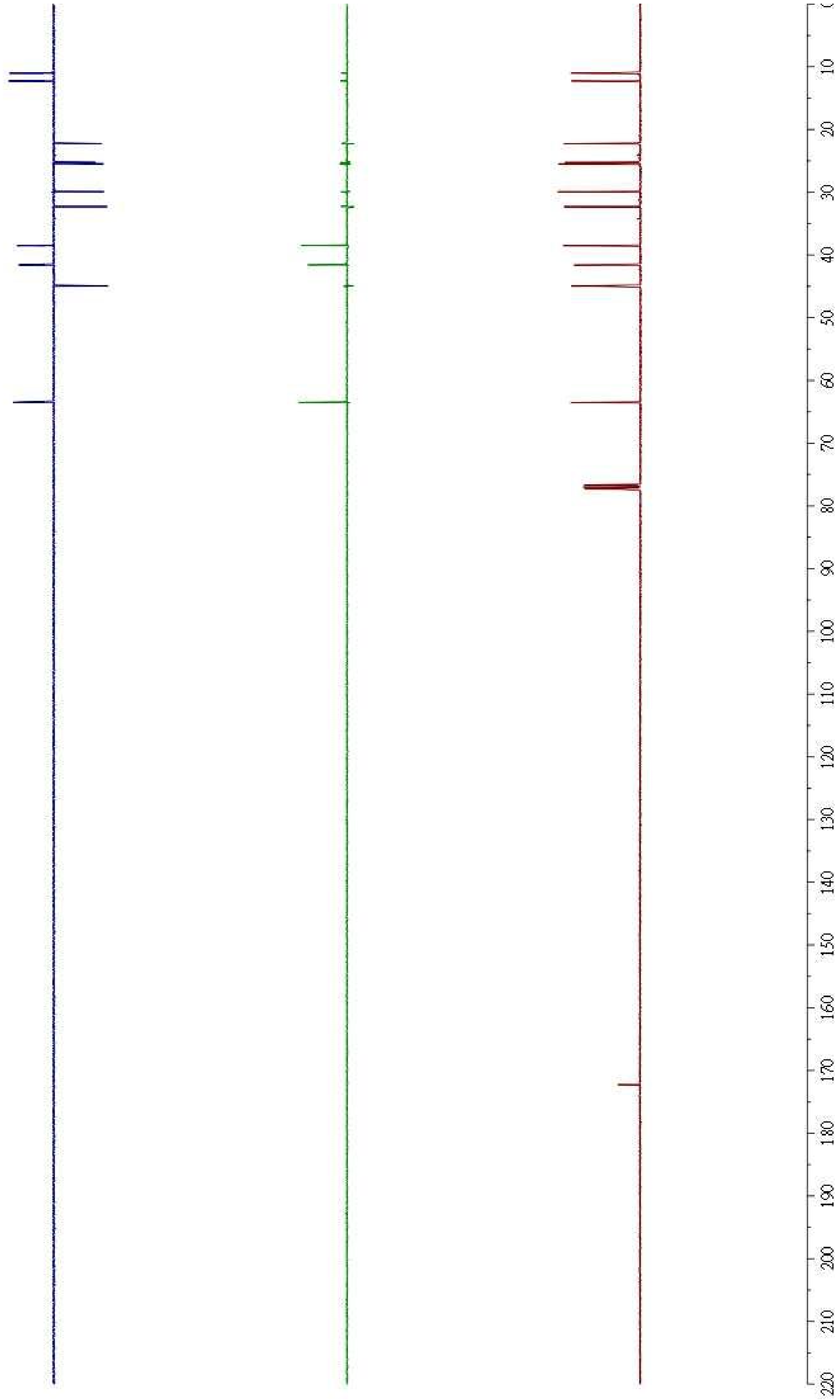

***epi*-7b**, ^{1}H-NMR (400MHz, $CDCl_3$)

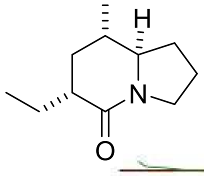

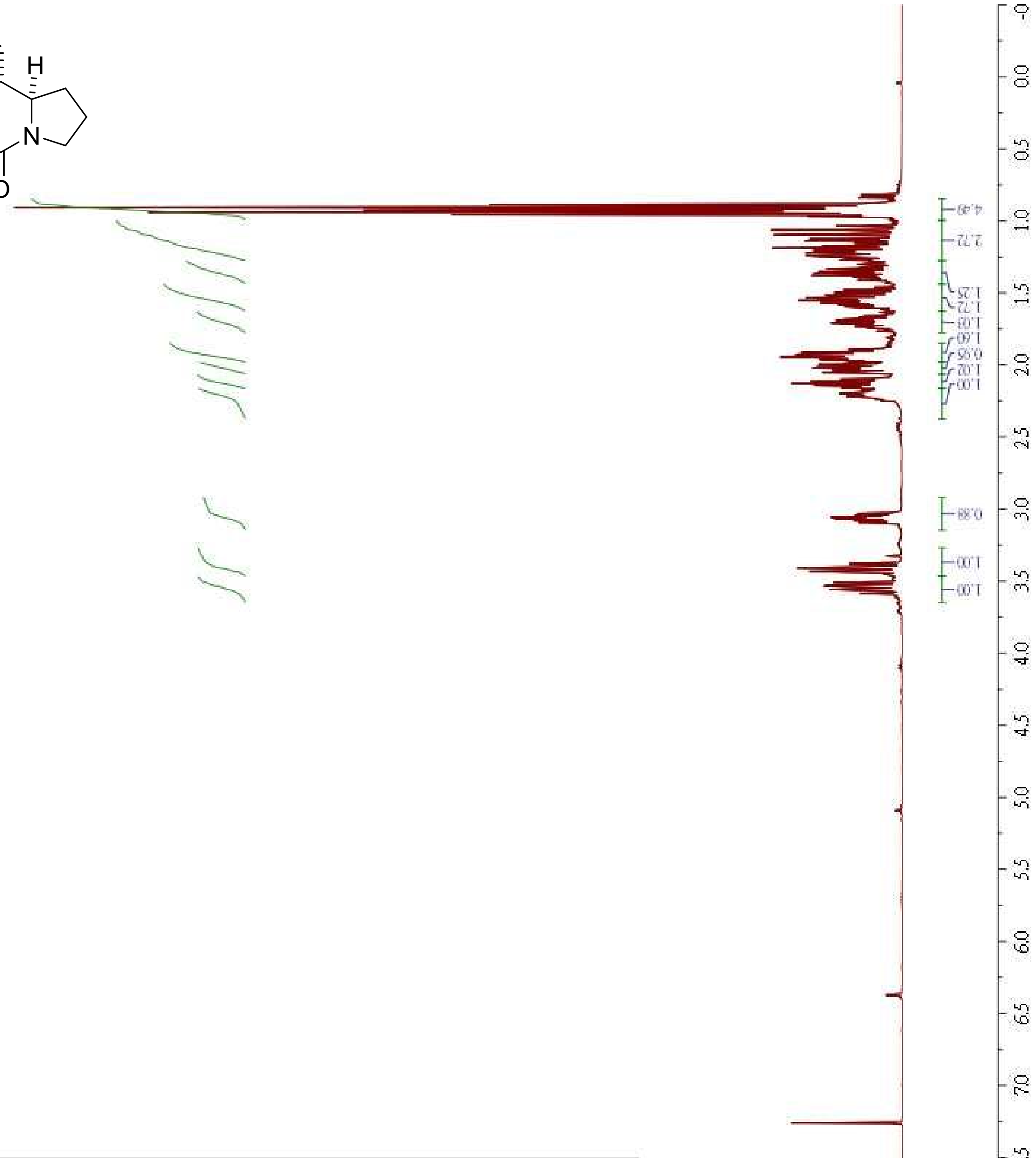

Parameters

	Parameter	Value
1	Title	wei457pf1_H_good
2	Origin	Varian
3	Spectrometer	vnmrs
4	Solvent	cdcl3
5	Temperature	20.0
6	Pulse Sequence	s2pul
7	Experiment	1D
8	Number of Scans	128
9	Receiver Gain	30
10	Relaxation Delay	1.0000
11	Pulse Width	0.0000
12	Acquisition Time	2.5559
13	Acquisition Date	2018-01-16T00:51:52
14	Modification Date	2019-04-08T22:09:16
15	Spectrometer Frequency	399.76
16	Spectral Width	6410.3
17	Lowest Frequency	-798.4
18	Nucleus	1H
19	Acquired Size	16384
20	Spectral Size	32768

***epi*-7b**, $^{13}C\{^{1}H\}$ NMR (101 MHz, $CDCl_3$)

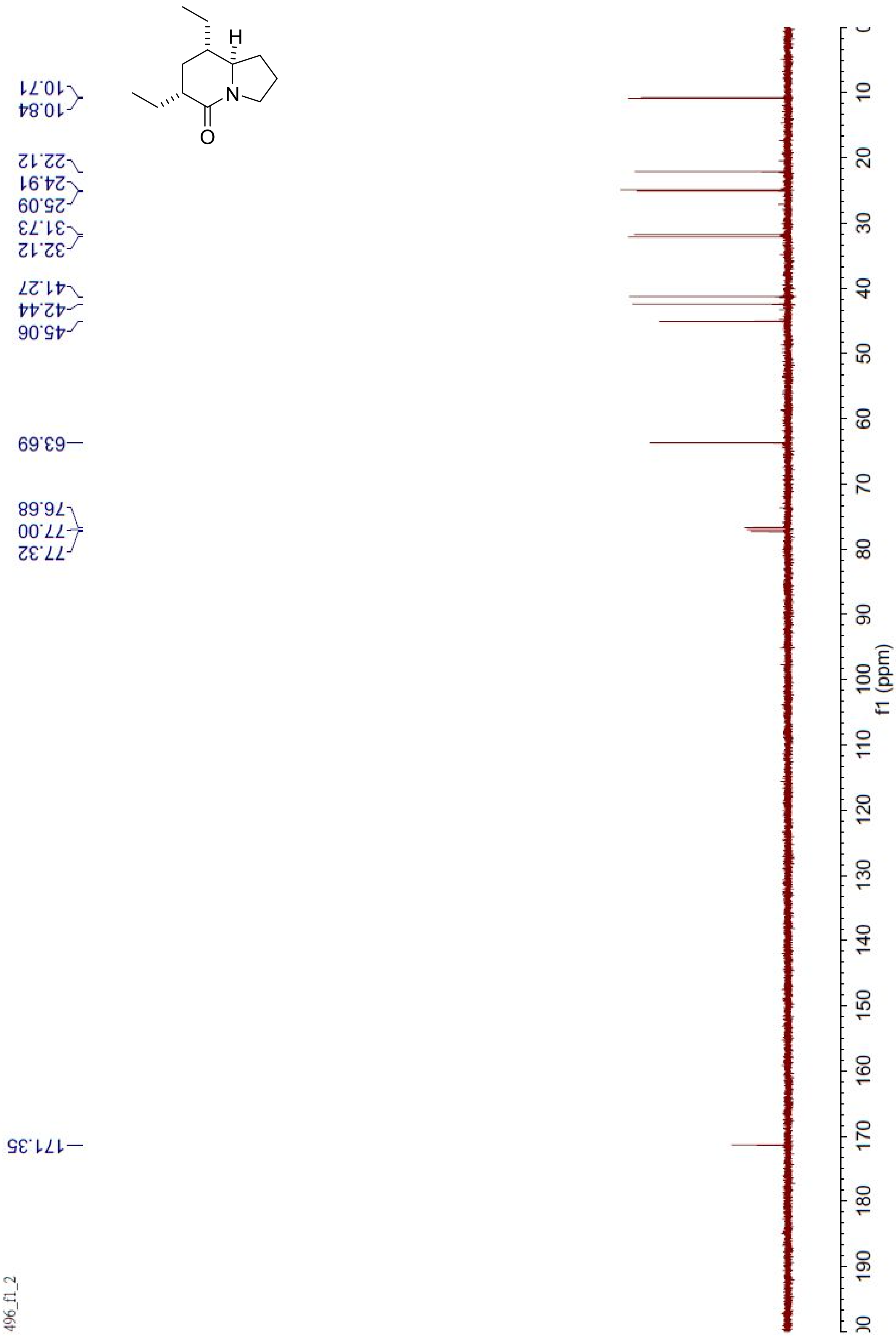

***epi*-7b**, DEPT (101 MHz, $CDCl_3$)

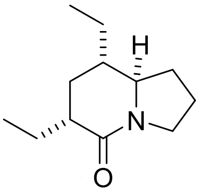

8, ^{1}H-NMR (400MHz, $CDCl_3$)

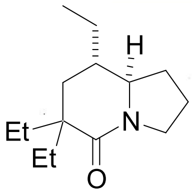

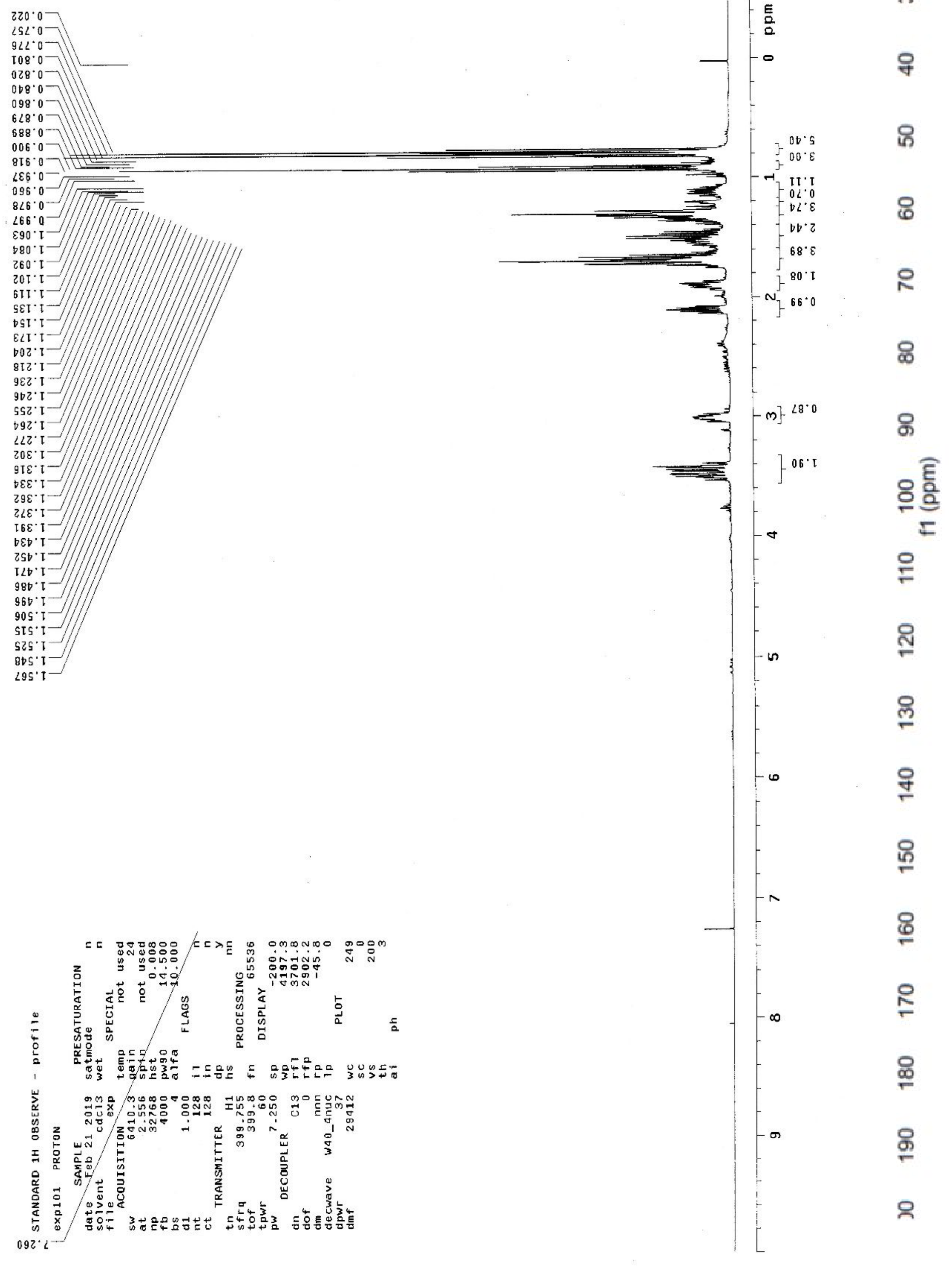

8, $^{13}C\{^{1}H\}$ NMR (101 MHz, $CDCl_3$)

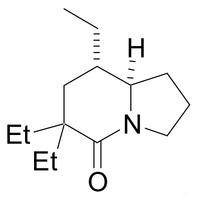

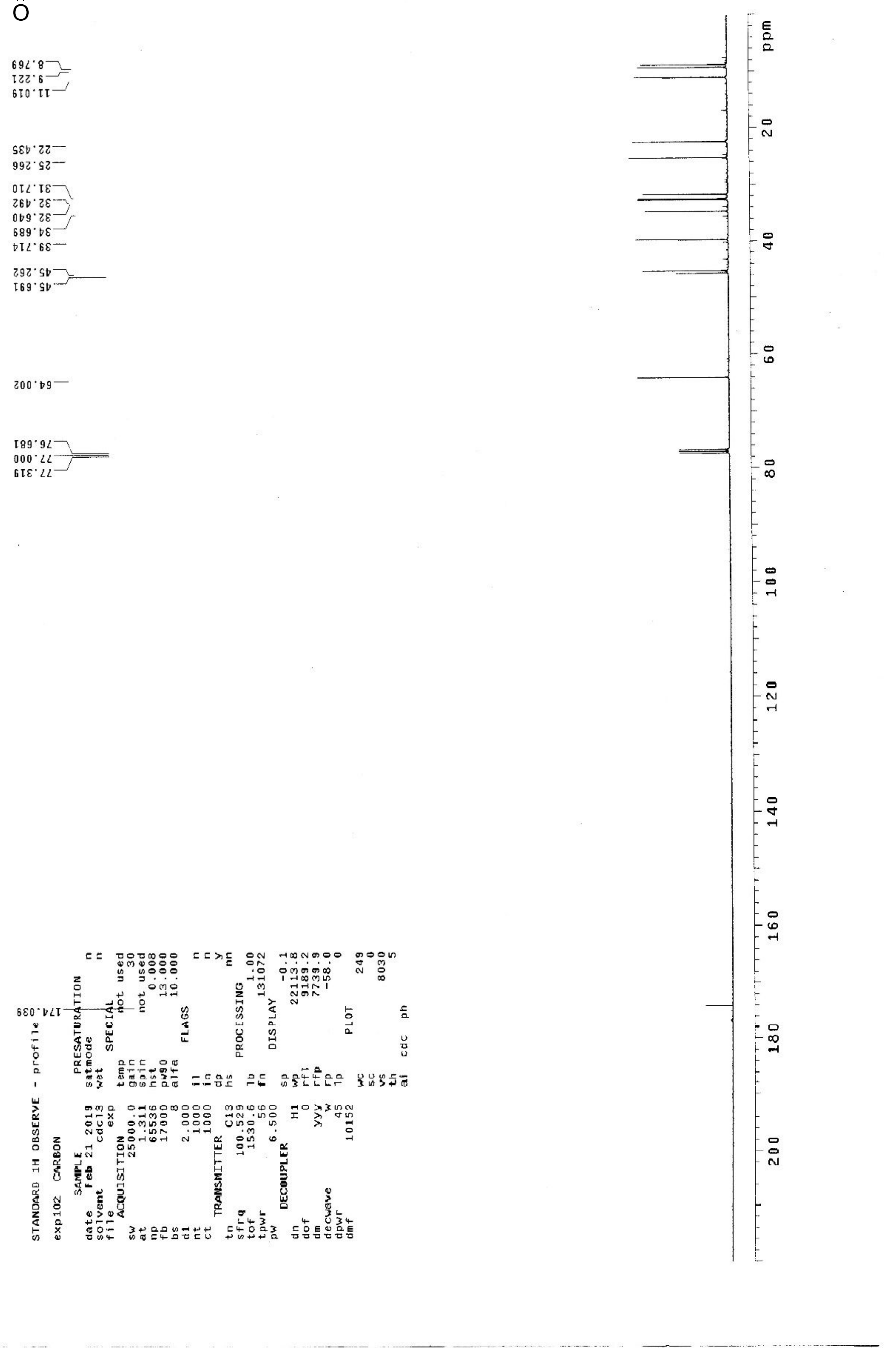

8, DEPT (101 MHz, $CDCl_3$)

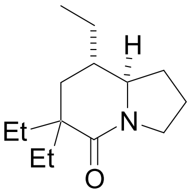

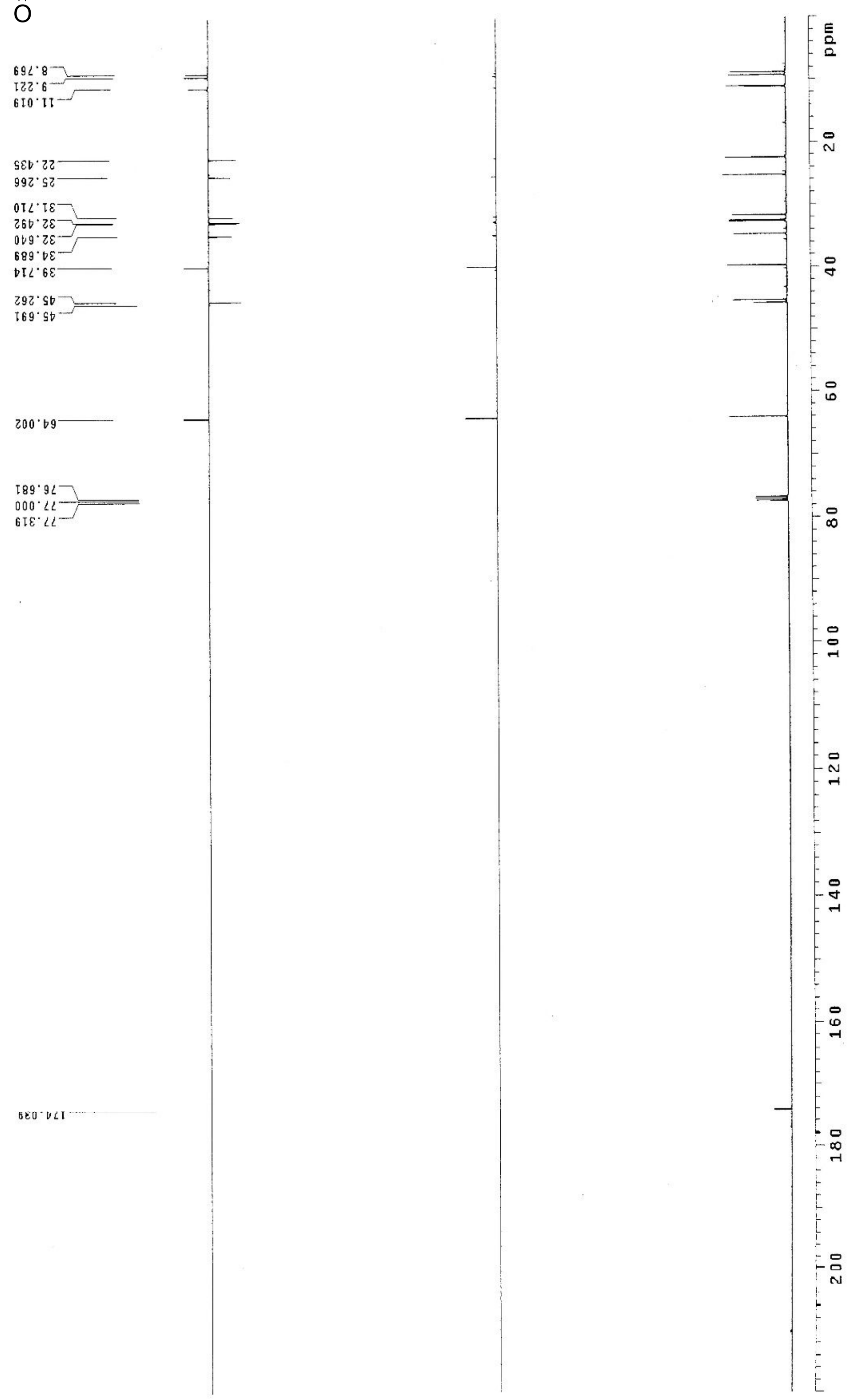

References:

1. Toyooka, N.; Fukutome, A.; Nemoto, H.; Daly, J. W.; Spande, T. F.; Garraffo, H. M.; Kaneko, T. Synthesis of Alkaloid 223A and a Structural Revision. *Org. Lett.* **2002,** *4*, 1715-1717.
2. Harris, J. M.; Padwa, A. A Flexible Approach toward Trisubstituted Piperidines and Indolizidines: Synthesis of 6-*epi*-Indolizidine 223A. *J. Org. Chem.* **2003,** *68*, 4371-4381.
3. Ghosh, P.; Judd, W. R.; Ribelin, T.; Aubé, J. Asymmetric Total Synthesis of Alkaloids 223A and 6- *epi*-223A. *Org. Lett.* **2009,** *11*, 4140-4142.
4. *Gaussian 09*, *Gaussian, Inc*.; 340 Quinnipiac St Bldg 40 Wallingford, CT 06492 USA.
5. (a) Becke, A. D. *J. Chem. Phys.* **1993**, *98*, 5648; (b) Lee C.; Yang, W.; Parr, R. G. *Phys. Rev. B* **1988**, *37*, 785.
